# Plasma proteomic profiling of septic shock and acute pancreatitis identifies shared signatures and disease-specific pathways

**DOI:** 10.1038/s41598-026-70106-3

**Published:** 2026-09-06

**Authors:** Jon Wallner, Asanda Mazubane, Jaap van der Heijden, Egor Vorontsov, Marko Sallisalmi, Michael Marks-Hultström, Jyrki Tenhunen, Annelie Barrueta Tenhunen

**Affiliations:** 1 Anesthesiology and Intensive Care Medicine, Department of Surgical Sciences, Uppsala University, Uppsala, Sweden; 2 Proteomics Core Facility, University of Gothenburg, Gothenburg, Sweden; 3 Perioperative and Intensive Care, Helsinki University Hospital, Helsinki, Finland; 4 Integrative Physiology, Department of Medical Cell Biology, Uppsala University, Uppsala, Sweden

**Keywords:** Septic shock, Pancreatitis, Biomarkers, Proteomics, Pathways, Biomarkers, Diseases, Immunology, Medical research

## Abstract

Septic shock represents the most severe form of infection-driven systemic inflammation, whereas acute pancreatitis induces a sterile inflammatory response. Although clinically similar, their molecular profiles may reveal distinct mechanisms underlying infectious and non-infectious inflammation. We performed plasma proteomic profiling using LC–MS/MS in patients with septic shock (*n* = 13), acute pancreatitis (*n* = 8), and healthy controls (*n* = 8). Among 663 quantified proteins, 231 were differentially expressed in septic shock versus controls, 83 in pancreatitis versus controls, and 29 in septic shock versus pancreatitis. Septic shock was characterized by higher plasma concentrations of MARCKS, HSP90AA1, PSAP, CD163, and GANAB, whereas pancreatitis showed higher levels of CPA1, APOC4, APOC3, BPGM, and APOC2. Cluster analysis demonstrated separation between groups, with overlapping proteomic patterns in sepsis and pancreatitis. Gene Ontology and KEGG analyses revealed shared inflammatory signatures, including upregulation of acute-phase responses and downregulation of coagulation pathways. However, septic shock exhibited more extensive proteomic alterations, with distinct activation of PI3K–Akt signaling and suppression of lipid metabolism. In conclusion, septic shock and pancreatitis share common inflammatory pathways, while proteomic differences highlight divergent regulation of coagulation, lipid metabolism, and anti-inflammatory signaling, offering potential biomarkers to distinguish infectious from sterile systemic inflammation.

**Supplementary Information:**

The online version contains supplementary material available at https://doi.org/10.1038/s41598-026-70106-3.

## Introduction

A dysregulated host response to infection is the hallmark of sepsis^1^. As sepsis progresses to septic shock, disruption of homeostasis across a number of biological pathways becomes increasingly pronounced, resulting in increased morbidity and mortality^2^. Acute pancreatitis also causes a dysregulated inflammatory response and is associated with substantial mortality. In contrast to sepsis, pancreatitis initially constitutes a sterile inflammation with no bacterial component^3,4^. However, secondary infections leading to sepsis compose a serious later-stage complication^3,5^ and is associated with increased mortality^6^. The two conditions exhibit similar clinical and biochemical features, including systemic inflammatory response, organ failure and commonly used laboratory markers, that can make differential diagnosis difficult^7,8^. Whether sterile inflammation mobilizes the same inflammatory pathways that orchestrate the septic response, or relies on fundamentally different signaling systems, remains incompletely understood^1^.

Proteomic profiling using mass spectrometry enables an unbiased assessment of the molecular response to disease by quantifying thousands of proteins simultaneously^9,10^. Because this approach does not rely on predefined targets or assumptions about which pathways are involved, it allows unbiased identification of proteins and the use of high-level analysis to classify them into pathways.^11^ Proteomic profiling has been used to identify biomarkers and underlying molecular mechanisms of sepsis^12–15^ and pancreatitis^16–18^. Proteomic signatures have also been applied to distinguish among sepsis phenotypes^19,20^ as well as to compare it to inflammation caused by other etiologies such as Covid-19^21^, trauma-associated inflammation^22^ and pediatric hemophagocytic lymphohistiocytosis^23^. However, direct comparison of plasma protein profiles in septic shock and acute pancreatitis has not previously been reported.

In the present study, we sought to characterize and compare the plasma proteomes of patients with septic shock, representing a dysregulated host response to infection, and patients with alcohol-induced acute pancreatitis, representing sterile systemic inflammatory response. We hypothesized that although the two conditions would share proteomic signatures, their profiles would also reveal distinct biomarkers and pathways capable of differentiating the infection-driven host response in septic shock from the systemic response of sterile inflammation.

Methods.

### Participants and sample handling

This study constitutes a secondary analysis of previously published results^24,25^. Patients were enrolled consecutively from the intensive care unit (ICU) at Helsinki University Hospital from September 2008 to September 2009, including 13 with septic shock and 8 with acute pancreatitis. Eight healthy adult volunteers working at Helsinki University Hospital served as controls. Septic shock patients met the following inclusion criteria: age ≥ 18 years, ≥ 2 SIRS criteria, and norepinephrine requirement ≥ 0.1 mcg/kg/min after fluid resuscitation for suspected or confirmed infection. Pancreatitis patients were included if ≥ 18 years, CT-confirmed acute pancreatitis, ≥ 2 SIRS criteria, and requiring organ support (vasopressors, renal replacement therapy or mechanical ventilation). Whole blood was collected in K2EDTA tubes within 24 h of ICU admission. Plasma was separated by centrifugation (2500 g, 15 min, 18 °C), aliquoted, and stored at − 80 °C until analysis.

### Proteomic sample preparation and LC-MS/MS analysis

Proteomic sample preparation and combined liquid chromatography with tandem mass spectrometry **(**LC-MS/MS) analysis has been described earlier^24^. In brief, abundant plasma proteins were depleted through the treatment of individual samples with Pierce™ Top 12 Abundant Protein Depletion Spin Columns (Thermo Fisher Scientific), and the eluates were digested with trypsin and labelled with isobaric Tandem Mass Tag (TMT 10plex) reagents (Thermo Fisher Scientific). Pooled peptide samples were analyzed on an Orbitrap Fusion Tribrid or Q Exactive HF mass spectrometer interfaced with an Easy-nLC 1000 or 1200 liquid chromatography system (all from Thermo Fisher Scientific). The resulting LC-MS/MS raw files were processed using Proteome Discoverer version 2.4 (Thermo Fisher Scientific). There were 1503 proteins identified in total. No imputation of missing values was applied. After removing proteins with any missing values, we obtained a dataset of 663 proteins with valid quantification values across all samples.

Proteomic analysis was conducted in two stages using TMT labelling (10/11-plex batches). Batch effects were addressed using a pooled reference bridging strategy. Protein abundances were expressed as median-normalized ratios to the stage 1 reference pool to account for global loading differences, and presented as log2-transformed rations.

### Statistical analyses

Differentially expressed proteins were identified using ANOVA across all three groups, corrected for false discovery rate (FDR) using the Benjamini-Hochberg method. Individual differences between the groups were analyzed using Tukey’s HSD. Analysis was performed using Python v3.10.12. and the pandas library v2.2.3^26^. Proteins were classified as upregulated (log2 fold change > 0.5) or downregulated (<-0.5) (Supplementary Table S1); this threshold was selected to maximize sensitivity in this exploratory study with limited sample size. For differential expression analysis, FDR < 0.05 was considered statistically significant.

A hierarchical clustering heatmap of the 250 proteins significantly altered in at least one comparison was generated using R v4.5.1 with packages limma and pheatmap^27,28^. Proteins with missing values or zero variance were removed; remaining values were log-transformed and z-scored by row.

Principal Component Analysis (PCA) was performed in Python v3.10.12 using scikit-learn. Analysis was restricted to Control, Septic shock, and Pancreatitis groups. Missing values were imputed with per-protein means, and data were z-score standardized. The first two principal components were visualized as a scatter plot with group-specific markers and 95% confidence ellipses. PCA was used as an unsupervised exploratory method without inferential statistical testing.

GO enrichment analysis (Biological Process, Molecular Function, Cellular Component) and KEGG pathway analysis were performed using R v4.5.1 with clusterProfiler, enrichKEGG, and org.Hs.eg.db.^29–31^. For both analyses, *p* < 0.05 and FDR < 0.2 were considered significant.

## Results

### Study participants

The study included patients with septic shock (*n* = 13), acute pancreatitis (*n* = 8), and healthy controls (*n* = 8, Table 1). The proportion of males was higher in the pancreatitis group while patients with septic shock were older on average. Disease severity scores were markedly higher in septic shock compared with acute pancreatitis, yet ICU stays were comparable between these groups. The 28-day mortality was 15.4% among septic shock patients, while no deaths occurred in the pancreatitis group.

**Table 1:** Descriptive data of the groups of septic shock, acute pancreatitis and healthy controls. APACHE2 and SOFA score at admission. APACHE II=Acute Physiology and Chronic Health Evaluation 2. SOFA=Sequential Organ Failure Assessment.

	Septic shock (*N* = 13)	Pancreatitis (*N* = 8)	Control (*N* = 8)
Male – *n* (%)	8 (62%)	7 (88%)	4 (50%)
Age – Yr (mean ± SD)	56.8±12.1	48.1±9.4	45.9±11
APACHE II score (mean ± SD)	24.9±6.0	10.8±3.4	N/A
SOFA score (mean ± SD)	12.9±3.0	8.1±2.4	N/A
Days of ICU (mean ± SD)	9.7 ± 3.9	12.3±8.7	N/A
28-day mortality – n (%)	2 (15%)	0	N/A

### Proteomic analysis

**Fig. 1:**
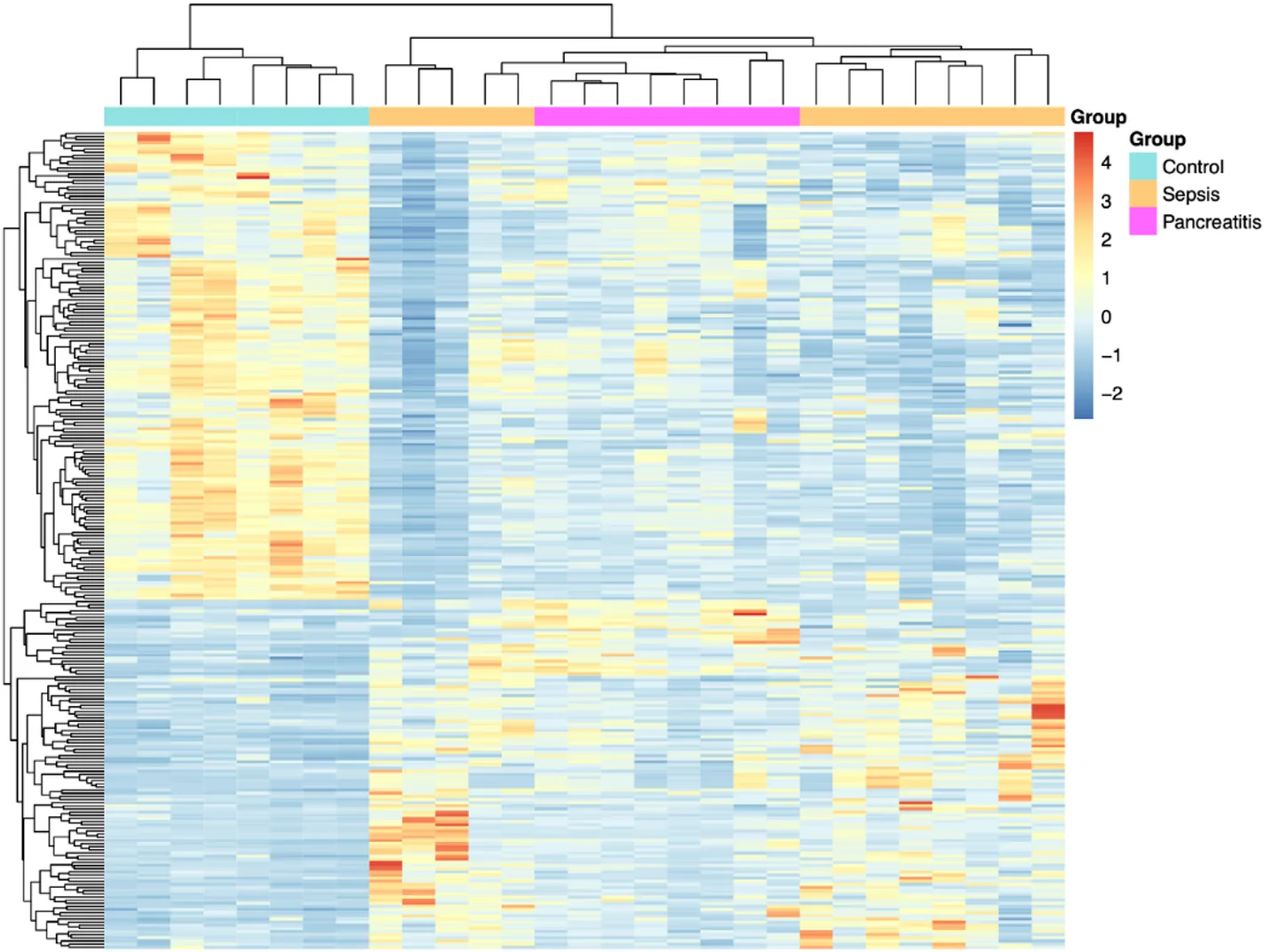
Heatmap generated with R v4.5.1 with limma and pheatmap^27,28^, illustrating the hierarchical clustering of the 250 differentially expressed proteins across controls (*n* = 8), septic shock (*n* = 13) and acute pancreatitis (*n* = 8). Each row represents a protein. Protein expression levels are standardized as Z-scores, where red indicates upregulation and blue indicates downregulation relative to the mean across all groups. The dendrogram on the left indicates hierarchical clustering of proteins based on similarity in expression patterns.

**Fig. 2:**
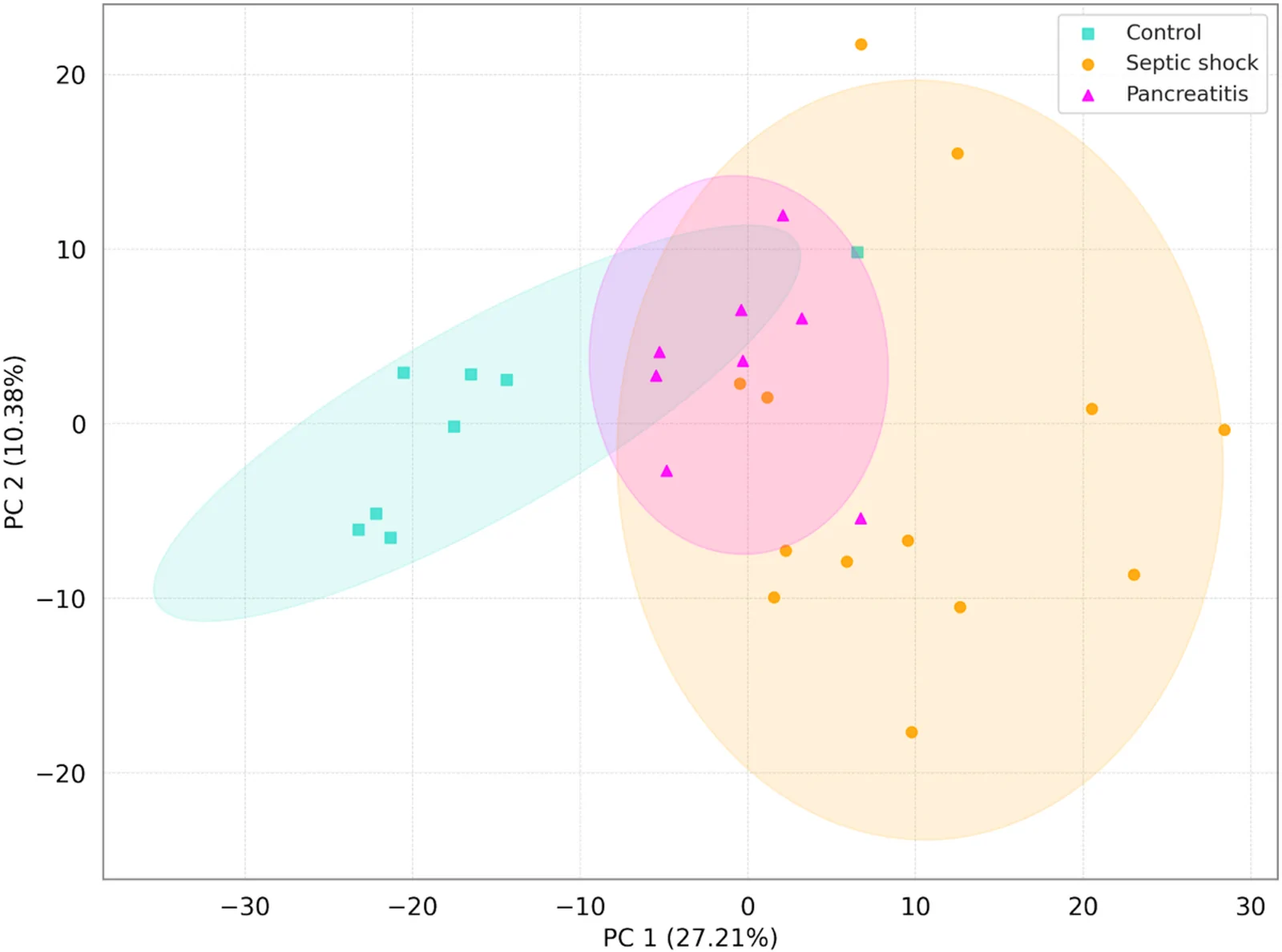
PCA performed on 663 proteins measured in control (*n* = 8), septic shock (*n* = 13), and acute pancreatitis (*n* = 8). Each point represents an individual patient, and the first two principal components (PC1 and PC2) explain the total variance, respectively. Ellipses represent the 95% confidence intervals for each group.

Hierarchical clustering clearly separated the groups based on their protein expression profiles, with clusters corresponding largely to control, septic shock, and pancreatitis samples (Fig. 1). Controls formed a distinct cluster characterized primarily by lower relative expression values, while septic shock samples showed a broader distribution with several proteins displaying marked upregulation. Pancreatitis samples formed a third cohesive cluster exhibiting elevated expression of a separate subset of proteins. These group-level expression patterns were consistent with trends observed in the principal component analysis (PCA, Fig. 2).

**Fig. 3:**
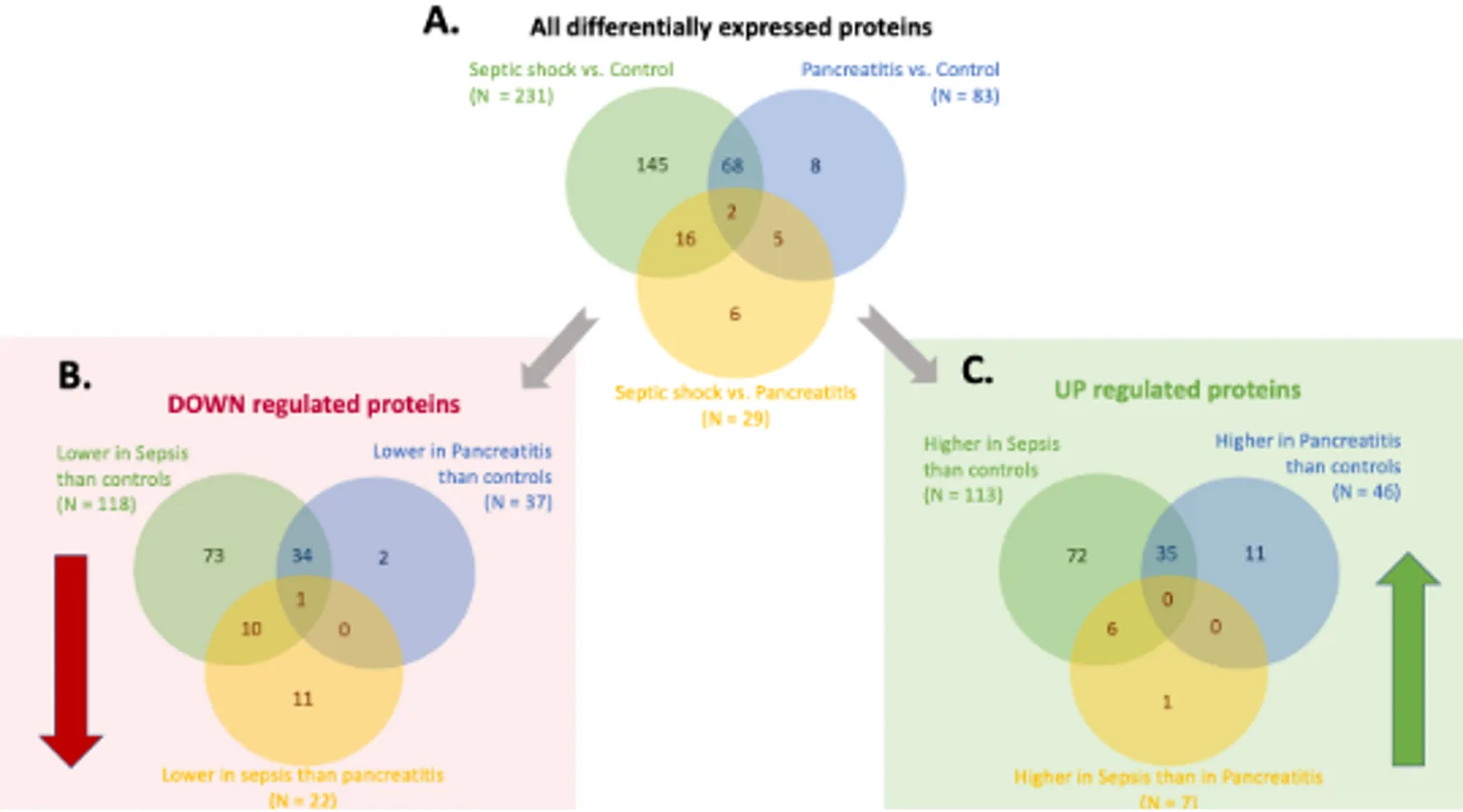
Venn diagrams showing the distribution of significantly differentially expressed proteins (DEPs) as absolute numbers for the comparisons septic shock vs. control (green circles), pancreatitis vs. control (blue circles) and septic shock vs. pancreatitis (yellow circles). Numbers in the overlapping areas represents proteins significantly altered in multiple comparisons. **(A)** All significantly altered proteins. **(B)** Only significantly downregulated proteins. **(C)** Only significantly upregulated proteins.

The plasma proteome analysis of patients with septic shock, acute pancreatitis, and healthy controls identified a total of 663 unique proteins, with 250 differentially expressed in at least one comparison (Supplementary Tables S2-S4). Comparing septic shock to controls identified 231 DEPs (Fig. 3A), of which 113 were upregulated (Fig. 3B) and 118 were downregulated (Fig. 3C). In pancreatitis compared to controls 83 DEPs were found, of which 46 were upregulated and 37 were downregulated. Comparison of septic shock and acute pancreatitis identified 29 DEPs, 7 higher in septic shock and 22 higher in pancreatitis.

**Fig. 4:**
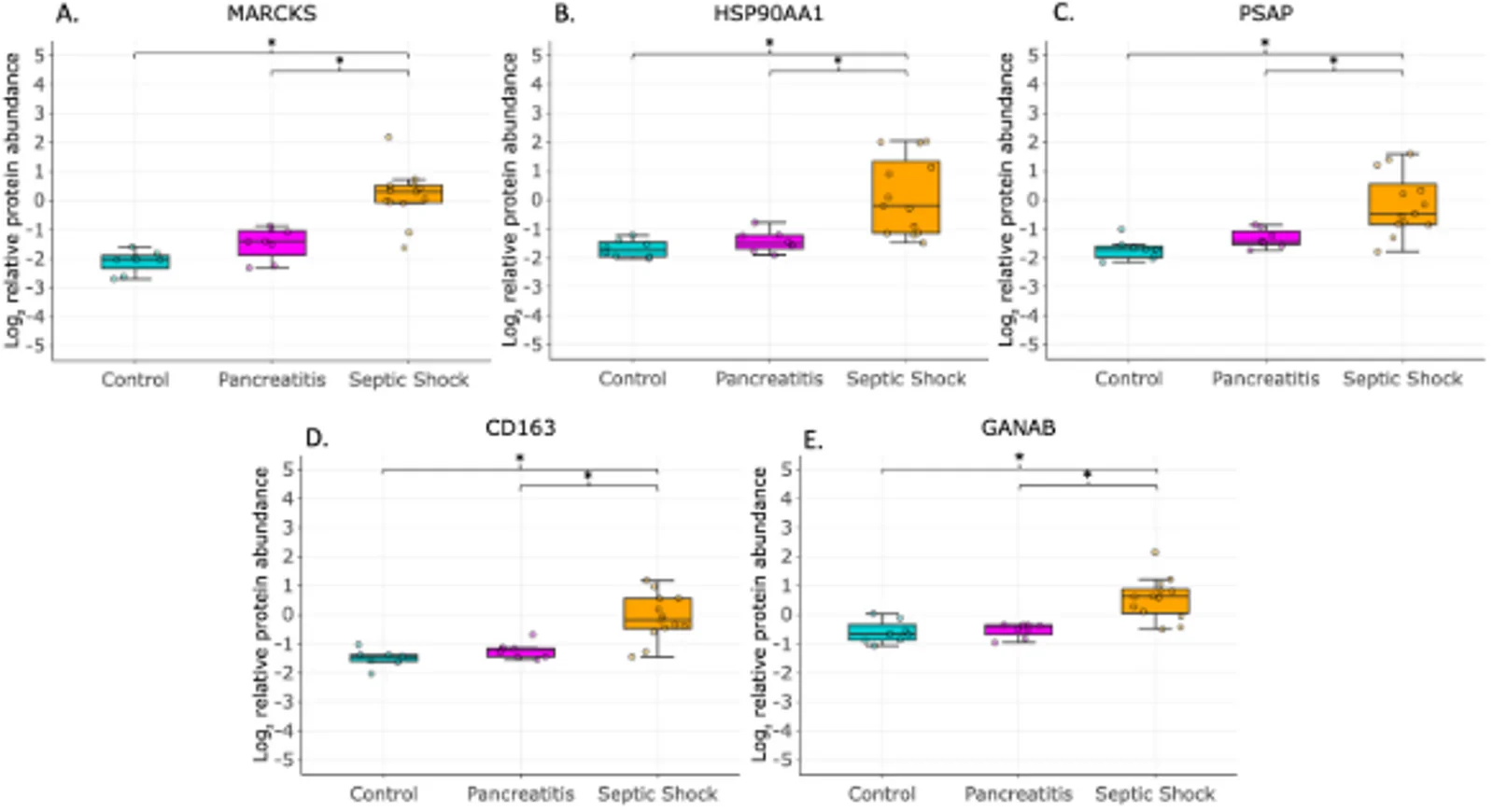
Box plots of the five most highly expressed proteins in septic shock compared to pancreatitis. Boxes show median values and interquartile range (IQR). Each data point is represented by a circle. * indicates significance between groups (adjusted p-value < 0.05).

**Fig. 5:**
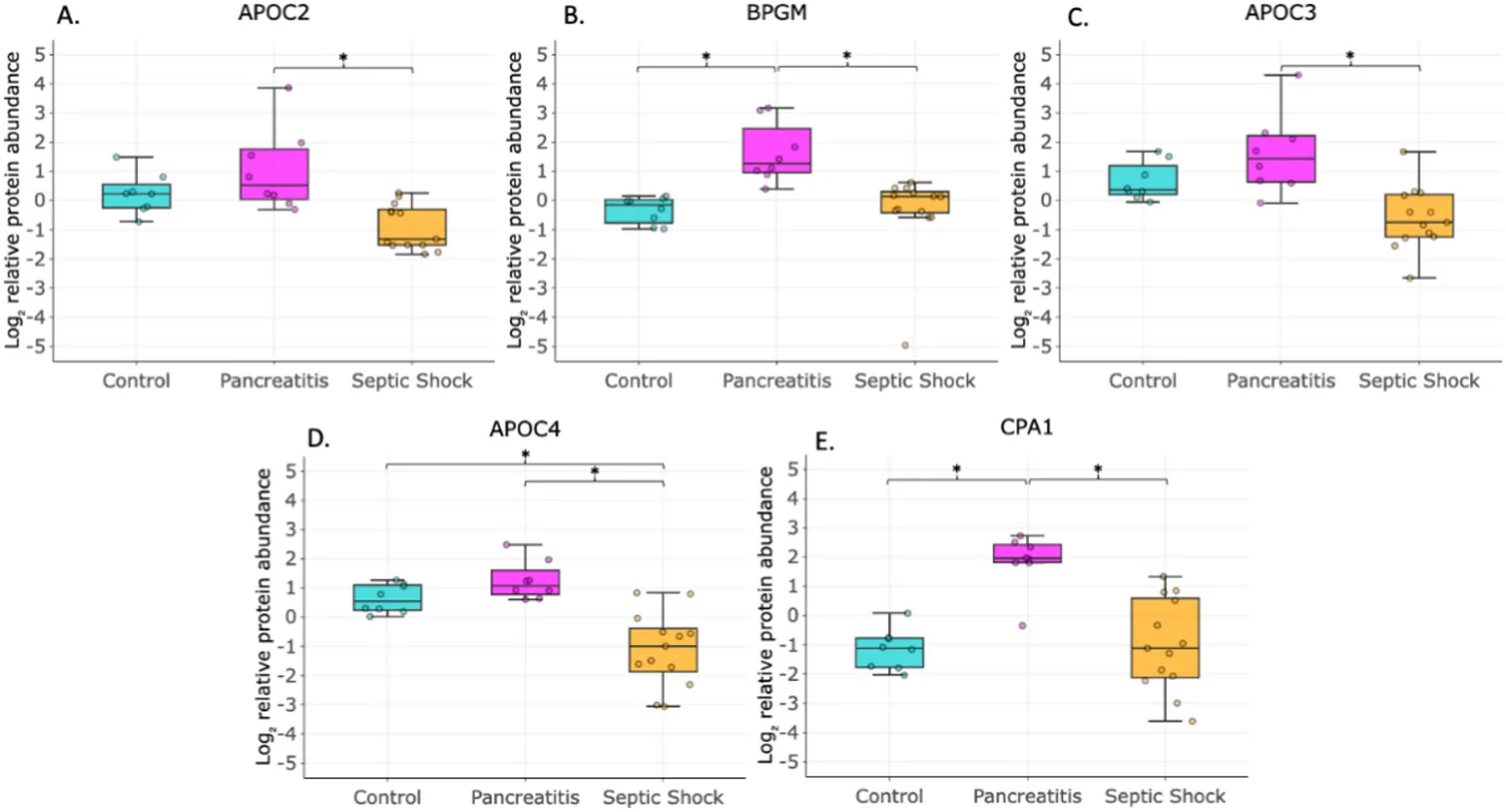
Box plots of the five proteins with the lowest expression in septic shock compared to pancreatitis. Boxes show median values and interquartile range (IQR). Each data point represented by a circle. * indicates significance between groups (adjusted p-value < 0.05).

MARCKS (AUC 0.913), HSP90AA1 (0.913), PSAP (0.855), CD163 (0.894), and GANAB (0.923) exhibited the greatest increases in septic shock relative to pancreatitis and were also significantly elevated in septic shock compared with controls (Fig. 4). In contrast, CPA1 (AUC 0.952), APOC4 (0.962), APOC3 (0.933), BPGM (0.971), and APOC2 (0.933) showed the highest expression in pancreatitis compared to septic shock. Relative to controls, BPGM and CPA1 were specifically increased in pancreatitis, whereas APOC4 was decreased in septic shock (Fig. 5).

### Gene ontology

DEPs were used for Gene Ontology (GO) enrichment analysis to evaluate concerted changes across different biological systems within the domains of biological process, cellular component, and molecular function. Significant enrichment was observed across all three GO categories (Supplementary Fig. S1-S3; Supplementary Tables S5-S7).

### Septic shock vs. control

**Fig. 6:**
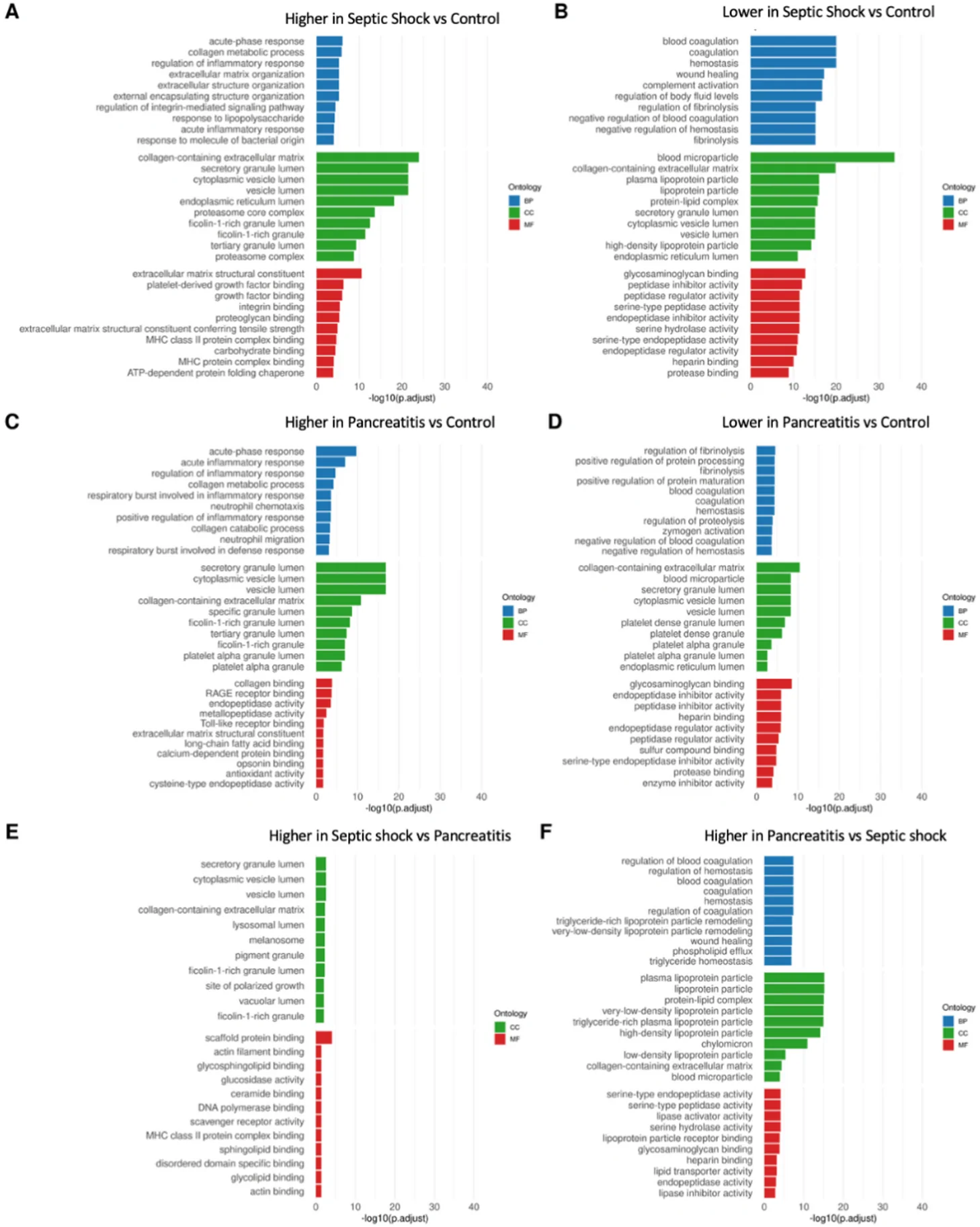
Bar plots displaying Gene Ontology (GO) enrichment analysis of differentially expressed proteins performed across **(A)** Higher in septic shock vs. control, **(B)** Lower in septic shock vs. control, **(C)** Higher in pancreatitis vs. control, **(D)** Lower in pancreatitis vs. control, **(E)** Higher in septic shock vs. pancreatitis and **(F)** Higher in pancreatitis vs. septic shock. The top 10 significantly enriched terms are shown for each category, biological process (BP, blue), cellular component (CC, green) and molecular function (MF, red) respectively along the y-axis. The x-axis represents the log10(adjusted p-value) which indicates the strength of significance (*p* < 0.05; q < 0.2).

Biological processes related to inflammation, immune response, collagen metabolism, and extracellular matrix were enriched among proteins that were more highly expressed in septic shock (Fig. 6A), whereas processes related to coagulation, hemostasis, and wound healing were enriched among proteins decreased in septic shock (Fig. 6B).

### Pancreatitis vs. control

Biological processes related to inflammation, immune response, and collagen metabolism were enriched among proteins that were more highly expressed in pancreatitis compared to control (Fig. 6C). Processes related to coagulation, hemostasis, and protein processing were enriched among proteins that were expressed at lower levels in pancreatitis versus control (Fig. 6D).

### Septic shock vs. pancreatitis

No biological processes were significantly enriched among proteins that were more highly expressed in septic shock compared to acute pancreatitis (Fig. 6E). Terms related to coagulation, hemostasis, wound healing, lipoprotein remodeling, and lipase regulation were significantly enriched among proteins more highly expressed in pancreatitis (Fig. 6F).

### KEGG pathways

Pathway annotation and enrichment analyses based on the Kyoto Encyclopedia of Genes and Genomes (KEGG) database were performed using the DEPs identified across comparisons (Supplementary Fig. S4, Supplementary Tables S8-S10).

### Septic shock vs. control

**Fig. 7:**
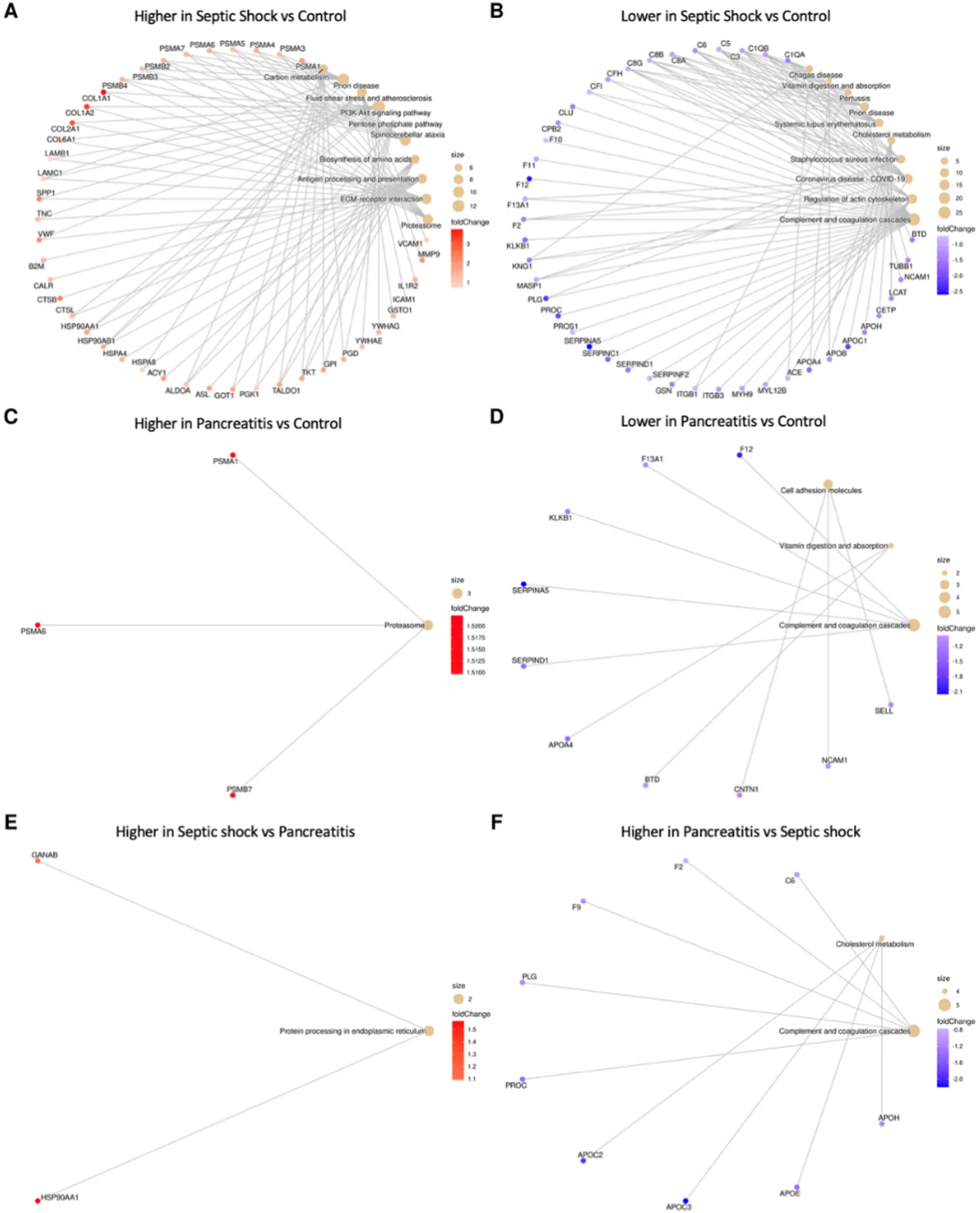
Category network plots displaying KEGG pathway (Kanehisa et al., 2025) enrichment analysis of differentially expressed proteins performed across **(A)** Higher in septic shock vs. control, **(B)** Lower in septic shock vs. control, **(C)** Higher in pancreatitis vs. control, **(D)** Lower in pancreatitis vs. control, **(E)** Higher in septic shock vs. pancreatitis and **(F)** Higher in pancreatitis vs. septic shock. The top 10 significantly enriched pathway nodes (represented by beige circles) are connected to their associated protein via edges (gray lines), indicating shared functional relationships. The color gradient from light red to dark red for upregulated proteins and light blue to dark blue for downregulated proteins indicates gene expression changes measured by log_2_ fold change and corresponds to the pathway-connected associated protein.

Enrichment analysis of the upregulated DEPs in septic shock revealed significant associations with 23 pathways (Supplementary Table S8a), the ten most significant are represented in the figure (Fig. 7A). The pathway with the greatest number of upregulated proteins was identified as the PI3K-Akt signaling pathway, with 13 DEPs. Similarly, twelve pathways were associated with downregulated DEPs, with the ten most significant in the figure (Fig. 7B). The pathway associated with the highest number of proteins being the complement and coagulation cascades, with 27 associated DEPs.

### Pancreatitis vs. control

In the pancreatitis group the proteasome was the only pathway significantly enriched among upregulated DEPs (Fig. 7C). Three pathways were associated with downregulated DEPs, with the pathway of complement and coagulation cascades associated with the highest amount of downregulated proteins (Fig. 7D).

### Septic shock vs. pancreatitis

When comparing septic shock to pancreatitis, the only pathway associated with upregulated proteins was protein processing in the endoplasmic reticulum (Fig. 7E). The downregulated DEPs were enriched in complement and coagulation cascades and cholesterol metabolism (Fig. 7F).

## Discussion

By comparing both conditions to healthy controls and to each other, this three-group design enables differentiation between shared inflammatory responses and disease-specific proteomic signatures. The main finding of this study is that septic shock and acute pancreatitis share similar changes in their plasma proteomic profiles, particularly in the activation of core inflammatory pathways. Whilst this overlap was observed, septic shock also showed suppression of lipid and cholesterol metabolism, dysregulation of complement and coagulation cascades, and activation of PI3K–Akt signaling compared to pancreatitis. Within this context, a subset of differentially expressed proteins emerged as candidate biomarkers for distinguishing infection-driven from sterile systemic inflammation.

MARCKS, HSP90AA1, PSAP, CD163, and GANAB showed the strongest increases in septic shock compared to pancreatitis, with AUC (Area Under the Curve) values ranging from 0.855 to 0.923. These proteins were also significantly elevated in septic shock relative to controls, but showed no significant differences between pancreatitis and controls, indicating they may be specific to septic shock. Among these, HSP90AA1 and CD163 were notable for their established network centrality in prior literature. HSP90AA1 engages in hundreds of protein-protein interactions within eukaryotic cells, functioning as a central molecular chaperone hub^32^. Additionally, it encodes the molecular chaperone HSP90α, which is involved in NLRP3 inflammasome activation^33,34^, reactive oxidative species (ROS) release and ferroptosis^35^. HSP90α has been observed elevated in systemic inflammation and further increased in cases of severe sepsis^36^, consistent with our observations. CD163 has been identified as the top hub gene in sepsis-related PPI network analysis^37^. CD163 is a macrophage- and monocyte-specific receptor that can be shed as a soluble form (sCD163) in response to macrophage activation in inflammation^38^. sCD163 has been observed elevated and proposed as a prognostic marker in sepsis^39–41^, in line with our findings. MARCKS is involved in vascular permeability, leukocyte adhesion and migration^42–44^. Prosaposin (PSAP) is a precursor to saposins, proteins involved in apoptosis and antigen presentation^45^. In sepsis, their role remains unclear, previously reported both elevated^46^ and decreased^47^ compared to controls. GANAB encodes the catalytic α-subunit of glucosidase II, which forms a heterodimer with the β-subunit (PRKCSH) to trim N-glycans from glycoproteins in the endoplasmic reticulum (ER)^48,49^. In our analysis, PRKCSH was likewise upregulated in septic shock compared with controls, further supporting the involvement of this pathway. While glucosidase II upregulation has been documented in other diseases^48,49^, the direct association with septic shock is novel in our analysis. This finding lends credence to the role of ER-stress in sepsis^50^.

Overall, pancreatitis showed milder proteomic disturbance than septic shock with both fewer affected proteins and lower magnitudes of average change. However, CPA1 and BPGM were specifically increased in pancreatitis, whilst APOC2, APOC3 and APOC4 showed expression of being lower in septic shock, all proteins with strong discriminatory capacity between the two conditions (AUC 0.933–0.971). CPA1 encodes a pancreatic digestive exopeptidase secreted by acinar cells, and its mutations have been linked to both acute and chronic pancreatitis^51^. BPGM is involved in regulating hemoglobin’s affinity for oxygen, and has been linked to myocardial injury in sepsis^52^. No additional associations with sepsis or pancreatitis have been reported. APOC2 is a component of chylomicrons, HDL, and VLDL and activates lipoprotein lipase (LPL)^53^. APOC2 deficiency leads to severe hypertriglyceridemia and is associated with an increased risk of pancreatitis^53^. APOC3 acts antagonistically to APOC2 by inhibiting LPL and promoting circulating triglycerides, and has been reported to be decreased in sepsis and septic shock^54,55^. The function of APOC4 is less well characterized, though it has been linked to VLDL metabolism^56^; no clear associations with sepsis or pancreatitis have been reported.

The enrichment analysis showed that acute-phase response and acute inflammatory response were among the most enriched upregulated GO-terms in both sepsis and pancreatitis, consistent with previous reports^12–15^, and the idea of a mostly in-common inflammatory response. The most enriched GO terms and pathways among downregulated DEPs in both septic shock and pancreatitis, relative to controls, were associated with coagulation and hemostasis. This is consistent with previous sepsis studies^12,14,15^, but contrasts with prior studies in pancreatitis, which have reported only modest enrichment^17^ or enrichment in upregulated proteins^16^. This pattern aligns with complement activation and consumption, as commonly described in sepsis^57,58^. The coagulation-related DEPs included proteins with opposing functional effects, reflecting the mixed coagulopathy that characterizes both sepsis and acute pancreatitis^59,60^. When comparing septic shock with pancreatitis, the complement and coagulation cascades pathway, along with GO terms related to coagulation and hemostasis, were enriched among DEPs elevated in pancreatitis, indicating greater consumption of these proteins in septic shock.

Cholesterol metabolism was enriched pathway among proteins more highly expressed in pancreatitis compared to septic shock. Furthermore, several apolipoproteins were found to be suppressed in septic shock compared to both pancreatitis and controls (Supplementary Tables S2 and S4). This aligns with established literature linking hypocholesterolemia to adverse outcomes in sepsis^61,62^. While suppressed HDL is a known feature of systemic inflammation^63,64^, including pancreatitis^65,66^, our data suggest a potentially more pronounced alteration of cholesterol metabolism in septic shock compared to sterile inflammation. The PI3K-Akt signaling pathway was the most enriched KEGG pathway among proteins upregulated in septic shock versus control. This pathway regulates cell survival, cell growth, and metabolism whilst serving as a key regulator of inflammation by moderating activation of inflammatory cells^67,68^.

The lack of pathway enrichment in the comparison between sepsis and pancreatitis reemphasizes the overall similarity in the inflammatory activation on the proteome level. However, the individual differentially expressed proteins (MARCKS, HSP90AA1, PSAP, CD163, GANAB, CPA1, APOC4, APOC3, BPGM and APOC2) may represent candidate biomarkers for differentiating septic shock and acute pancreatitis, although diagnostic sensitivity and specificity must be validated in a larger cohort.

The main strength of the present study is the direct comparison of plasma proteins from patients with septic shock and pancreatitis with healthy controls using mass spectrometry to provide comprehensive, unbiased proteomic profiling. The resource-intensive mass spectrometry-based proteomics also comes with a limited sample size that reduces external validity and statistical power. Technical limitations inherent to mass spectrometry-based proteomics include variable protein detectability, with low-abundance proteins, membrane-associated proteins and highly hydrophobic proteins typically underrepresented. As this study was based on plasma proteomics, intracellular proteomic changes could not be assessed, and it remains difficult to distinguish whether alterations in protein concentration reflect differences in synthesis, degradation, or consumption. Acute pancreatitis and septic shock are also heterogeneous and dynamic syndromes, which may further influence results, particularly in a study of this sample size. However, our findings are broadly consistent with previous proteomic studies of sepsis^12–15^ providing confidence in the findings. Finally, this study demonstrates associations rather than causality. The results should therefore be considered exploratory and hypothesis-generating, warranting validation in larger cohorts and confirmation through complementary experimental approaches.

## Conclusion

Septic shock and acute pancreatitis display a largely similar pattern of proteomic regulation, characterized by shared activation of acute-phase and inflammatory pathways and downregulation of coagulation-related proteins. Despite this overlap, distinct differences emerged between the conditions. Dysregulation of coagulation was more pronounced in septic shock, both in number and magnitude. Septic shock also showed a specific upregulation of PI3K–Akt signaling and a more marked suppression of lipid and cholesterol metabolism, whereas several apolipoproteins were preserved in pancreatitis.

## Supplementary Information

Below is the link to the electronic supplementary material.

Supplementary Material 1

## Data Availability

The mass spectrometry proteomics data generated and analyzed during this study have been deposited in the PRIDE repository, a member of the ProteomeXchange Consortium^69^, and are publicly available under the dataset identifier PXD080484 or using the following link: https://www.ebi.ac.uk/pride/archive/projects/PXD080484. During the review process the dataset is available using the dataset identifier PXD080484 and the reviewer token eX6JRw3JoLjG, which can be entered at the following link: https://www.ebi.ac.uk/pride/login.

## Abbreviations

SIRS: Systemic Inflammatory Response Syndrome
ICU: Intensive Care Unit
LC-MS/MS: Liquid Chromatography-Tandem Mass Spectrometry
DEP: Differentially Expressed Protein
FDR: False Discovery Rate
PCA: Principal Component Analysis
GO: Gene Ontology
KEGG: Kyoto Encyclopedia of Genes and Genomes
AUC: Area Under the Curve
ER: Endoplasmic Reticulum
HDL: High Density Lipoprotein
VLDL: Very Low-Density Lipoprotein
LPL: Lipoprotein Lipase
