# Supplementary material for: Plasma proteomic profiling of septic shock and acute pancreatitis identifies shared signatures and disease-specific pathways
Source: Sci Rep. 2026 Sep 6;16:27826. doi: 10.1038/s41598-026-70106-3 (PMC13547407; doi:10.1038/s41598-026-70106-3)
Supplement: Supplementary file 1 [file 41598_2026_70106_MOESM1_ESM.pdf]

Supplementary Material to:

Plasma Proteomic Profiling of Septic Shock and Acute Pancreatitis Identifies Shared Signatures and Disease-specific Pathways

Jon Wallner, Asanda Mazubane, Jaap van der Heijden, Egor Vorontsov, Marko Sallisalmi, Michael Marks-Hultström, Jyrki Tenhunen, Annelie Barrueta Tenhunen

Table S1a. Counts of proteins meeting differential expression conditions for significant upregulation across three condition groups.

	Count		
Significance conditions	Septic shock vs. Control	Pancreatitis vs. Control	Septic shock vs. Pancreatitis
Log2FC > 0.5	227	189	155
Adjusted p-value < 0.05	231	83	29
Significantly upregulated	113	46	7

Septic shock vs. Control: ACY1', 'ADAMTSL2', 'ALDOA', 'ARHGAP1', 'ASL', 'B2M', 'BASP1', 'CALR', 'CAST', 'CCN2', 'CD163', 'CD177', 'CHI3L1', 'COL11A2', 'COL1A1', 'COL1A2', 'COL2A1', 'COL5A1', 'COL6A1', 'CRP', 'CST3', 'CTSB', 'CTSD', 'CTSL', 'CTSZ', 'DDT', 'DPP3', 'EPHB4', 'F8', 'FAH', 'FGL2', 'FSTL1', 'GALNT2', 'GANAB', 'GOLM1', 'GOT1', 'GPI', 'GRN', 'GSTO1', 'H3-4', 'H4C1; H4C2; H4C3; H4C4; H4C5; H4C6; H4C8; H4C9; H4C11; H4C12; H4C13; H4C14; H4C15; H4-16', 'HSP90AA1', 'HSP90AB1', 'HSPA4', 'HSPA8', 'ICAM1', 'IGFBP4', 'IL18BP', 'IL1R2', 'IL1RL1', 'ITIH3', 'LAMB1', 'LAMC1', 'LBP', 'LCN2', 'LCP1', 'LGALS3BP', 'LMNB1', 'LRG1', 'LTA4H', 'LTBP2', 'MARCKS', 'MMP8', 'MMP9', 'MPO', 'MRC1', 'NID1', 'NPC2', 'NUCB1', 'ORM1', 'PDIA4', 'PGD', 'PGK1', 'PLA2G2A', 'PLIN3', 'PRKCSH', 'PSAP', 'PSMA1', 'PSMA3', 'PSMA4', 'PSMA5', 'PSMA6', 'PSMA7', 'PSMB2', 'PSMB3', 'PSMB4', 'REG1A', 'REG1B', 'RRBP1', 'S100A12', 'S100A8', 'S100A9', 'SAA1', 'SAA2', 'SEMA4B', 'SERPINA3', 'SERPINB1', 'SIRPA', 'SIRPB1', 'SOD2', 'SPP1', 'TALDO1', 'TIMP1', 'TKT', 'TNC', 'TRAP1', 'VCAM1', 'VCAN', 'VIM', 'VSIG4', 'VWF', 'YWHAE', 'YWHAG'

Pancreatitis vs. Control: ‘APOA5', 'BPGM', 'CA1', 'CAT', 'CDHR2', 'CHI3L1', 'COL6A1', 'CPA1', 'CRP', 'CTSB', 'CTSD', 'CTSZ', 'EPHB4', 'F8', 'GALNT2', 'IGFBP4', 'IL1RL1', 'LBP', 'LCP1', 'LRG1', 'LTA4H', 'MAN1A1', 'MMP8', 'MMP9', 'NID1', 'NUCB1', 'ORM1', 'ORM2', 'PRDX2', 'PRSS2', 'PSMA1', 'PSMA6', 'PSMB7', 'REG1B', 'S100A12', 'S100A8', 'S100A9', 'SAA1', 'SAA2', 'SERPINA3', 'TALDO1', 'TIMP1', 'TKT', 'VIM', 'VSIG4', 'VWF'

Septic shock vs. Pancreatitis: 'CD163', 'COTL1', 'GANAB', 'HSP90AA1', 'MARCKS', 'PSAP', 'SERPINB1'

Table S1b. Counts of proteins meeting differential expression conditions for significant downregulation across three condition groups.

	Count		
Significance conditions	Septic shock vs. Control	Pancreatitis vs. Control	Septic shock vs. Pancreatitis
Log2FC < -0.5	222	169	152
Adjusted p-value < 0.05	231	83	29
Significantly downregulated	118	37	22

Septic shock vs. Control: 'A1BG', 'ACE', 'ADAMTS13', 'ADGRG6', 'ADIPOQ', 'AFM', 'AHSG', 'AOC3', 'APOA4', 'APOB', 'APOC1', 'APOC4', 'APOD', 'APOF', 'APOH', 'APOM', 'ATRN', 'AZGP1', 'B3GNT2', 'B4GAT1', 'BCHE', 'BIN2', 'BTD', 'C1QA', 'C1QB', 'C1RL', 'C3', 'C5', 'C6', 'C8A', 'C8B', 'C8G', 'CACNA2D1', 'CDH13', 'CETP', 'CFH', 'CFI', 'CHL1', 'CLEC3B', 'CLU', 'CNDP1', 'CNTN1', 'CNTN3', 'COLEC10', 'CPB2', 'CRISP3', 'CRTAC1', 'DBH', 'DPP4', 'ECM1', 'ENG', 'ENPEP', 'F10', 'F11', 'F12', 'F13A1', 'F2', 'FAP', 'FCN3', 'FETUB', 'GC', 'GPLD1', 'GPNMB', 'GSN', 'HABP2', 'HGFAC', 'HRG', 'IGFALS', 'IGFBP3', 'IGHV3-7', 'IL1RAP', 'ITGB1', 'ITGB3', 'ITIH1', 'ITIH2', 'ITIH4', 'KIT', 'KLKB1', 'KNG1', 'LAMP1', 'LCAT', 'LUM', 'MASP1', 'MCAM', 'MEGF8', 'MINPP1', 'MYH9', 'MYL12B', 'NCAM1', 'PCOLCE', 'PCYOX1', 'PGLYRP2', 'PI16', 'PLEK', 'PLG', 'PON1', 'PON3', 'PROC', 'PROS1', 'PROZ', 'PTPRJ', 'RAB10', 'RBP4', 'SELENOP', 'SELL', 'SERPINA4', 'SERPINA5', 'SERPINA6', 'SERPINA7', 'SERPINC1', 'SERPIND1', 'SERPINF2', 'SHBG', 'SPP2', 'SUN3', 'TTR', 'TUBB1', 'UMOD'

Pancreatitis vs. Control: 'ABI3BP', 'ACE', 'ADA2', 'AFM', 'AHSG', 'APOA4', 'APOD', 'ATRN', 'BTD', 'CDH13', 'CHL1', 'CLEC3B', 'CNDP1', 'CNTN1', 'CRISP3', 'CRTAC1', 'ECM1', 'F12', 'F13A1', 'FAP', 'FETUB', 'GPLD1', 'GPNMB', 'GSN', 'HGFAC', 'HRG', 'IGFBP3', 'ITIH1', 'ITIH2', 'KLKB1', 'NCAM1', 'PGLYRP2', 'SELENOP', 'SELL', 'SERPINA4', 'SERPINA5', 'SERPIND1']

Septic shock vs. Pancreatitis: 'AFM', 'APOA5', 'APOC2', 'APOC3', 'APOC4', 'APOE', 'APOF', 'APOH', 'BPGM', 'C6', 'CPA1', 'F2', 'F9', 'HABP2', 'LRG1', 'MAN1A1', 'PCYOX1', 'PLG', 'PRDX2', 'PROC', 'PTPRJ', 'SNCA'

Table S2 - Septic Shock versus Control

GENE SYMBOL	GENE NAME	NCBI GENE ID	UNIPROT ID	KEGG PATHWAYS	LOG2FC	-LOG10 P-VALUE	ADJUSTED P-VALUE
**CRP**	C-reactive protein	1401	P02741	-	4,40	13,76	0,00e+00
**MMP8**	Matrix metallopeptidase 8	4317	P22894	-	4,30	9,04	6,02e-08
**CHI3L1**	Chitinase-3-like protein 1 (YKL-40)	1116	P36222	-	4,15	8,22	2,23e-07
**IL1RL1**	Interleukin 1 receptor-like 1 (ST2)	9173	Q01638	Cytokine-cytokine receptor interaction (hsa04060)	4,02	9,80	1,54e-08
**COL1A1**	Collagen type I alpha 1 chain	1277	P02452	PI3K-Akt signaling pathway (hsa04151)<br>L interaction (hsa04512)<br>Focal adhesion (hsa04510)	3,75	3,45	1,92e-03
**COL1A2**	Collagen type I alpha 2 chain	1278	P08123	PI3K-Akt signaling pathway (hsa04151)<br>ECM-receptor interaction (hsa04512)<br>Focal adhesion (hsa04510)	3,37	3,72	1,12e-03

**COL2A1**	Collagen type II alpha 1 chain	1280	P02458	PI3K-Akt signaling pathway (hsa04151)<br>ECM-receptor interaction (hsa04512)<br>Focal adhesion (hsa04510)	3,11	2,60	9,94e-03
**SAA2**	Serum amyloid A2	6289	P0DJI9	-	3,03	4,06	5,72e-04
**SAA1**	Serum amyloid A1	6288	P0DJI8	-	2,90	4,83	1,31e-04
**REG1B**	Regenerating family member 1 beta	5968	P48304	-	2,86	5,73	2,55e-05
**REG1A**	Regenerating family member 1 alpha	5967	P05451	-	2,71	3,32	2,39e-03
**H4C1; H4C2; H4C3; H4C4; H4C5; H4C6; H4C8; H4C9; H4C11; H4C12; H4C13; H4C14; H4C15; H4-16**	Histone cluster 4, H4 variants (H4C1–H4C16)	Various	Various (e.g., P62805)	Neutrophil extracellular trap formation (hsa04613)<br>Systemic lupus erythematosus (hsa05322)<br>Alcoholism (hsa05034)	2,67	2,19	2,23e-02
**H3-4**	Histone H3.4 (H3 family member 4)	8290	Q16695	Neutrophil extracellular trap formation (hsa04613)<br>Systemic lupus erythematosus (hsa05322)<br>Alcoholism (hsa05034)	2,66	2,09	2,61e-02

**LCN2**	Lipocalin 2	3934	P80188	IL-17 signaling pathway (hsa04657)	2,66	5,48	3,92e-05
**MPO**	Myeloperoxidase	4353	P05164	Neutrophil extracellular trap formation (hsa04613)<br>Drug metabolism - other enzymes (hsa00983)<br>Phagosome (hsa04145)	2,55	5,27	5,91e-05
**COL11A2**	Collagen type XI alpha 2 chain	1302	P13942	Protein digestion and absorption (hsa04974)<br>Cytoskeleton in muscle cells (hsa04820)	2,52	2,81	6,44e-03
**VSIG4**	V-set and immunoglobulin domain-containing protein 4	11326	Q9H7M9	Complement and coagulation cascades (hsa04610)	2,48	5,24	6,20e-05
**S100A12**	S100 calcium-binding protein A12	6283	P80511	Cornified envelope formation (hsa04382)	2,46	2,17	2,27e-02
**TIMP1**	TIMP metallopeptidase inhibitor 1	7076	P01033	HIF-1 signaling pathway (hsa04066)	2,45	9,12	5,65e-08
**CTSB**	Cathepsin B	1508	P07858	Lysosome (hsa04142)<br>Antigen processing and presentation (hsa04612)<br>Apoptosis (hsa04210)	2,40	8,48	1,45e-07
**SPP1**	Secreted phosphoprotein 1	6696	P10451	Focal adhesion (hsa04510)	2,35	3,87	8,36e-04

				Toll-like receptor signaling pathway (hsa04620)<br>PI3K-Akt signaling pathway (hsa04151)			
**MARCKS**	Myristoylated alanine-rich C-kinase substrate	4082	P29966	Fc gamma R-mediated phagocytosis (hsa04666)<br>MicroRNAs in cancer (hsa05206)	2,27	5,68	2,68e-05
**NUCB1**	Nucleobindin 1	4924	Q02818	-	2,25	3,92	7,66e-04
**LBP**	Lipopolysaccharide-binding protein	3929	P18428	NF-kappa B signaling pathway (hsa04064)<br>Toll-like receptor signaling pathway (hsa04620)<br>Alcoholic liver disease (hsa04936)	2,24	3,89	8,10e-04
**IL18BP**	Interleukin 18-binding protein	10068	O95998	-	2,20	3,76	1,03e-03
**VCAN**	versican	1462	P13611	Cell adhesion molecules (hsa04514)<br>Cytoskeleton in muscle cells (hsa04820)	2,17	5,24	6,20e-05
**PLA2G2A**	Phospholipase A2 group IIA	5320	P14555	Arachidonic acid metabolism (hsa00590)<br>Glycerophospholipid	2,14	2,52	1,15e-02

				metabolism (hsa00564)<br>Linoleic acid metabolism (hsa00591)			
**TRAP1**	TNF receptor-associated protein 1	10131	Q12931	Parkinson disease (hsa05012)<br>Pathways of neurodegeneration - multiple diseases (hsa05022)	2,13	2,23	2,08e-02
**MMP9**	Matrix metallopeptidase 9	4318	P14780	IL-17 signaling pathway (hsa04657)<br>TNF signaling pathway (hsa04668)<br>Leukocyte transendothelial migration (hsa04670)	2,12	4,23	4,13e-04
**VWF**	von Willebrand factor	7450	P04275	PI3K-Akt signaling pathway (hsa04151)<br>Complement and coagulation cascades (hsa04610)<br>Platelet activation (hsa04611)	2,09	7,28	1,24e-06
**S100A8**	S100 calcium-binding protein A8 (Calgranulin A)	6279	P05109	IL-17 signaling pathway (hsa04657)<br>Cornified envelope formation (hsa04382)	2,09	2,55	1,09e-02
**SOD2**	Superoxide dismutase 2	6648	P04179	FoxO signaling pathway (hsa04068)	2,02	1,95	3,43e-02

				Peroxisome (hsa04146)<br>Longevity regulating pathway (hsa04211)			
**COL5A1**	Collagen type V alpha 1 chain	1291	P20908	Cytoskeleton in muscle cells (hsa04820)<br>Protein digestion and absorption (hsa04974)	1,95	2,62	9,50e-03
**DDT**	D-dopachrome tautomerase	1652	P30046	-	1,94	3,16	3,25e-03
**VIM**	Vimentin	7431	P08670	Cytoskeleton in muscle cells (hsa04820)<br>Epstein-Barr virus infection (hsa05169)<br>MicroRNAs in cancer (hsa05206)	1,90	4,08	5,54e-04
**PSMA1**	Proteasome 20s subunit alpha 1	5682	P25786	Proteasome (hsa03050)<br>Prion disease (hsa05020)<br>Pathways of neurodegeneration - multiple diseases (hsa05022)	1,88	4,12	5,18e-04
**ACY1**	Aminoacylase 1	95	Q03154	Arginine biosynthesis (hsa00220)<br>Metabolic pathways (hsa01100)<br>Biosynthesis of amino acids (hsa01230)	1,88	2,15	2,37e-02

**COL6A1**	Collagen type VI alpha 1	1291	P12109	PI3K-Akt signaling pathway (hsa04151)<br>ECM-receptor interaction (hsa04512)<br>Focal adhesion (hsa04510)	1,86	7,40	9,86e-07
**NID1**	Nidogen 1 (Entactin)	4811	P14543	Cytoskeleton in muscle cells (hsa04820)	1,84	11,14	1,20e-09
**HSP90AA1**	heat shock protein 90 alpha family class A member 1	3320	P07900	Protein processing in endoplasmic reticulum (hsa04141)<br>PI3K-Akt signaling pathway (hsa04217)<br>Antigen processing and presentation (hsa04612)	1,83	3,72	1,12e-03
**DPP3**	Dipeptidyl peptidase 3	10072	Q9NY33	-	1,82	5,02	8,92e-05
**GPI**	Glucose-6-phosphate isomerase	2821	P06744	Glycolysis/Gluconeogenesis (hsa00010)<br>Pentose phosphate pathway (hsa00030)<br>Starch and sucrose metabolism (hsa00500)	1,82	3,74	1,08e-03
**S100A9**	S100 calcium-binding protein A9	6280	P06702	IL-17 signaling pathway (hsa04657)	1,81	2,23	2,08e-02

				Cornified envelope formation (hsa04382)			
**ASL**	Argininosuccinate lyase	435	P04424	Arginine biosynthesis (hsa00220)<br>Biosynthesis of amino acids (hsa01230)<br>Metabolic pathways (hsa01100)	1,78	2,18	2,27e-02
**TKT**	Transketolase	7086	P29401	Pentose phosphate pathway (hsa00030)<br>Carbon metabolism (hsa01200)<br>Biosynthesis of amino acids (hsa01230)	1,75	5,33	5,20e-05
**GOT1**	Glutamic-oxaloacetic transaminase 1	2805	P17174	Alanine, aspartate and glutamate metabolism (hsa00250)<br>Phenylalanine, tyrosine and tryptophan biosynthesis (hsa00400)<br>Carbon metabolism (hsa01200)	1,73	2,65	9,03e-03
**CTSD**	Cathepsin D	1509	P07339	Sphingolipid signaling pathway (hsa04071)<br>Lysosome (hsa04142)<br>Apoptosis (hsa04210)	1,72	7,67	5,90e-07
**GOLM1**	Golgi membrane protein 1	51280	Q8NBJ4	-	1,67	5,70	2,62e-05

**TALDO1**	Transaldolase 1	6888	P37837	Pentose phosphate pathway (hsa00030)<br>Carbon metabolism (hsa01200)<br>Biosynthesis of amino acids (hsa01230)	1,66	4,99	9,36e-05
**LTBP2**	Latent TGF-beta binding protein 2	4053	Q14767	-	1,66	5,56	3,42e-05
**ORM1**	Orosomucoid 1	5004	P02763	-	1,65	2,66	8,88e-03
**CCN2**	Cellular communication network factor 2	1490	P29279	Apelin signaling pathway (hsa04371)<br>Hippo signaling pathway (hsa04390)	1,63	2,74	7,54e-03
**HSP90AB1**	Heat shock protein 90 alpha family class B member 1	3326	P08238	Protein processing in endoplasmic reticulum (hsa04141)<br>PI3K-Akt signaling pathway (hsa04151)<br>Antigen processing and presentation (hsa04612)	1,58	3,20	3,08e-03
**PSMA4**	Proteasome 20S subunit alpha 4	5685	P25789	Proteasome (hsa03050)<br>Prion disease (hsa05020)<br>Pathways of neurodegeneration - multiple diseases (hsa05022)	1,58	2,54	1,11e-02

**PSAP**	Prosaposin	5660	P07602	Lysosome (hsa04142)<br>Sphingolipid metabolism (hsa00600)	1,57	4,50	2,41e-04
**CTSZ**	Cathepsin Z	1522	Q9UBR2	Lysosome (hsa04142)<br>Apoptosis (hsa04210)	1,56	5,84	2,09e-05
**MRC1**	Mannose receptor C-type 1	4360	P22897	Phagosome (hsa04145)<br>Tuberculosis (hsa05152)	1,56	4,76	1,51e-04
**LMNB1**	Lamin B1	4001	P20700	Apoptosis (hsa04210)<br>Cytoskeleton in muscle cells (hsa04820)	1,55	2,45	1,31e-02
**PSMB2**	Proteasome 20S subunit beta 2	5690	P49721	Proteasome (hsa03050)<br>Prion disease (hsa05020)<br>Pathways of neurodegeneration - multiple diseases (hsa05022)	1,54	2,09	2,61e-02
**PLIN3**	Perilipin 3	10226	O60664	-	1,54	2,80	6,60e-03
**PGD**	Phosphogluconate dehydrogenase	5226	P52209	Pentose phosphate pathway (hsa00030)<br>Glutathione metabolism (hsa00480)<br>Carbon metabolism (hsa01200)	1,53	3,34	2,36e-03
**PSMA6**	Proteasome 20S subunit alpha 6	5687	P60900	Proteasome (hsa03050)<br>Prion disease (hsa05020)	1,51	2,72	7,79e-03

				Pathways of neurodegeneration - multiple diseases (hsa05022)			
**PSMA7**	Proteasome 20S subunit alpha 7	5688	O14818	Proteasome (hsa03050)<br>Prion disease (hsa05020)<br>Pathways of neurodegeneration - multiple diseases (hsa05022)	1,50	2,20	2,20e-02
**IL1R2**	Interleukin 1 receptor type 2	7850	P27930	Cytokine-cytokine receptor interaction (hsa04060)<br>Hematopoietic cell lineage (hsa04640)<br>Human T-cell leukemia virus 1 infection (hsa05166)	1,50	3,33	2,39e-03
**B2M**	Beta-2-microglobulin	567	P61769	Antigen processing and presentation (hsa04612)<br>Human cytomegalovirus infection (hsa05163)<br>Human T-cell leukemia virus 1 infection (hsa05166)	1,50	3,01	4,42e-03
**HSPA4**	Heat shock protein family A member 4	3308	P34932	Tight junction (hsa04530)<br>Antigen processing and presentation (hsa04612)<br>Lipid and atherosclerosis (hsa05417)	1,49	4,26	3,84e-04

**YWHAG**	Tyrosine 3-monooxygenase/tryptophan 5-monooxygenase activation protein gamma	7532	P61981	Cell cycle (hsa04110)<br>PI3K-Akt signaling pathway (hsa04151)<br>Hippo signaling pathway (hsa04390)	1,46	4,28	3,78e-04
**PSMB4**	Proteasome 20S subunit beta 4	5692	P28070	Proteasome (hsa03050)<br>Prion disease (hsa05020)<br>Pathways of neurodegeneration - multiple diseases (hsa05022)	1,43	2,21	2,16e-02
**CD177**	CD177 molecule	57126	Q8N6Q3	-	1,42	3,16	3,25e-03
**CD163**	CD163 molecule	9332	Q86VB7	-	1,40	4,61	2,03e-04
**GSTO1**	Glutathione S-transferase omega 1	9446	P78417	Glutathione metabolism (hsa00480)<br>Drug metabolism by cytochrome P450 (hsa00982)<br>Drug metabolism - other enzymes (hsa00983)	1,40	3,45	1,90e-03
**PRKCSH**	PRKCSH beta subunit glucosidase II	5589	P14314	Protein processing in endoplasmic reticulum (hsa04141)	1,39	4,17	4,68e-04
**PSMA3**	Proteasome 20S subunit alpha 3	5684	P25788	Proteasome (hsa03050)<br>Prion disease (hsa05020)	1,39	2,13	2,46e-02

				Pathways of neurodegeneration - multiple diseases (hsa05022)			
**CTSL**	Cathepsin L	1514	P07711	Lysosome (hsa04142)<br>Antigen processing and presentation (hsa04612)<br>Apoptosis (hsa04210)	1,37	3,28	2,60e-03
**FSTL1**	Follistatin like 1	11167	Q12841	-	1,37	3,50	1,75e-03
**PDIA4**	Protein disulfide isomerase family A member 4	9601	P13667	Protein processing in endoplasmic reticulum (hsa04141)<br>Thyroid hormone synthesis (hsa04918)<br>Vibrio cholerae infection (hsa05110)	1,37	2,52	1,15e-02
**YWHAE**	Tyrosine 3-monooxygenase/tryptophan 5-monooxygenase activation protein epsilon	7531	P62258	PI3K-Akt signaling pathway (hsa04151)<br>Cell cycle (hsa04110)<br>Hippo signaling pathway (hsa04390)	1,36	3,16	3,25e-03
**RRBP1**	Ribosome binding protein 1	6238	Q9P2E9	Protein processing in endoplasmic reticulum (hsa04141)	1,35	2,10	2,59e-02

**FAH**	Fumarylacetoacetate hydrolase	2184	P16930	Tyrosine metabolism (hsa00350)<br>Metabolic pathways (hsa01100)	1,31	2,32	1,73e-02
**LTA4H**	Leukotriene A4 hydrolase	4048	P09960	Arachidonic acid metabolism (hsa00590)<br>Metabolic pathways (hsa01100)	1,31	5,18	7,01e-05
**PSMB3**	Proteasome 20S subunit beta 3	5691	P49720	Proteasome (hsa03050)<br>Prion disease (hsa05020)<br>Pathways of neurodegeneration - multiple diseases (hsa05022)	1,29	1,82	4,40e-02
**LCP1**	Lymphocyte cytosolic protein 1	3936	P13796	-	1,29	4,32	3,50e-04
**CALR**	Calreticulin	811	P27797	Protein processing in endoplasmic reticulum (hsa04141)<br>Antigen processing and presentation (hsa04612)<br>Phagosome (hsa04145)	1,29	2,89	5,53e-03
**TNC**	Tenascin C	3371	P24821	PI3K-Akt signaling pathway (hsa04151)<br>Focal adhesion (hsa04510)<br>ECM-receptor interaction (hsa04512)	1,26	4,05	5,88e-04
**PSMA5**	Proteasome 20S subunit alpha 5	5686	P28066	Proteasome (hsa03050)<br>Prion disease (hsa05020)	1,23	1,97	3,32e-02

				Pathways of neurodegeneration - multiple diseases (hsa05022)			
**FGL2**	Fibrinogen like 2	10875	Q14314	-	1,21	3,19	3,08e-03
**CAST**	Calpastatin	831	P20810	Shigellosis (hsa05131)	1,21	2,52	1,15e-02
**ICAM1**	Intercellular adhesion molecule 1	3383	P05362	NF-kappa B signaling pathway (hsa04064)<br>Leukocyte transendothelial migration (hsa04670)<br>Cell adhesion molecules (hsa04514)	1,20	1,99	3,16e-02
**BASP1**	Brain abundant membrane attached signal protein 1	10409	P80723	-	1,20	3,37	2,23e-03
**GALNT2**	Polypeptide N-acetylgalactosaminyltransferase 2	2590	Q10471	Mucin type O-glycan biosynthesis (hsa00512)<br>Other types of O-glycan biosynthesis (hsa00514)<br>Metabolic pathways (hsa01100)	1,19	4,27	3,84e-04
**SIRPB1**	Signal regulatory protein beta 1	10326	O00241	Efferocytosis (hsa04148)<br>Osteoclast differentiation (hsa04380I	1,17	3,37	2,23e-03
**GANAB**	Glucosidase II alpha subunit	23193	Q14697	Protein processing in endoplasmic reticulum (hsa04141)	1,16	3,44	1,95e-03

				N-Glycan biosynthesis (hsa00510)<br>Metabolic pathways (hsa01100)			
**EPHB4**	EPH receptor B4	2050	P54760	Axon guidance (hsa04360)	1,16	4,97	9,59e-05
**IGFBP4**	Insulin like growth factor binding protein 4	3487	P22692	-	1,13	4,58	2,11e-04
**ALDOA**	Aldolase, fructose-bisphosphate A	226	P04075	Glycolysis/Gluconeogenesis (hsa00010)<br>Fructose and mannose metabolism (hsa00051)<br>Carbon metabolism (hsa01200)	1,12	2,07	2,70e-02
**SERPINA3**	Serpin family A member 3	12	P01011	-	1,11	4,13	5,12e-04
**ARHGAP1**	Rho GTPase activating protein 1	392	Q07960	-	1,11	2,09	2,61e-02
**F8**	Coagulation factor VIII	2157	P00451	Complement and coagulation cascades (hsa04610)	1,07	3,90	7,90e-04
**SERPINB1**	Serpin family B member 1	1992	P30740	-	1,00	4,72	1,63e-04
**NPC2**	NPC intracellular cholesterol transporter 2	10577	P61916	Lysosome (hsa04142)<br>Cholesterol metabolism (hsa04979)	1,00	2,44	1,36e-02
**LAMC1**	Laminin subunit gamma 1	3915	P11047	PI3K-Akt signaling pathway (hsa04151)	0,97	4,41	2,87e-04

				Focal adhesion (hsa04510)<br>ECM-receptor interaction (hsa04512)			
**VCAM1**	Vascular cell adhesion molecule 1	7412	P19320	NF-kappa B signaling pathway (hsa04064)<br>Cell adhesion molecules (hsa04514)<br>TNF signaling pathway (hsa04668)	0,96	3,12	3,50e-03
**LGALS3BP**	Galectin 3 binding protein	3959	Q08380	-	0,93	2,33	1,71e-02
**PGK1**	Phosphoglycerate kinase 1	5230	P00558	Glycolysis/Gluconeogenesis (hsa00010)<br>Carbon metabolism (hsa01200)<br>Biosynthesis of amino acids (hsa01230)	0,91	1,90	3,76e-02
**SIRPA**	Signal regulatory protein alpha	140885	P78324	Efferocytosis (hsa04148)<br>Osteoclast differentiation (hsa04380I	0,90	2,54	1,11e-02
**CST3**	Cystatin C	1471	P01034	Salivary secretion (hsa04970)	0,90	2,59	1,01e-02
**GRN**	Granulin precursor	2896	P28799	-	0,89	1,92	3,62e-02
**HSPA8**	Heat shock protein family A (Hsp70) member 8	3312	P11142	Spliceosome (hsa03040)<br>MAPK signaling pathway (hsa04010)	0,87	1,94	3,52e-02

				Protein processing in endoplasmic reticulum (hsa04141)			
**LRG1**	Leucine rich alpha-2-glycoprotein 1	116844	P02750	-	0,85	2,99	4,53e-03
**ADAMTSL2**	ADAMTS like 2	9719	Q86TH1	-	0,82	1,92	3,60e-02
**SEMA4B**	Semaphorin 4B	10509	Q9NPR2	Axon guidance (hsa04360)	0,74	2,26	1,97e-02
**LAMB1**	Laminin subunit beta 1	3912	P07942	PI3K-Akt signaling pathway (hsa04151)<br>Focal adhesion (hsa04510)<br>ECM-receptor interaction (hsa04512)	0,72	1,93	3,54e-02
**ITIH3**	Inter-alpha-trypsin inhibitor heavy chain 3	3699	Q06033	-	0,50	1,79	4,72e-02
**MINPP1**	Multiple inositol-polyphosphate phosphatase 1	9562	Q9UNW1	Glycolysis/Gluconeogenesis (hsa00010)<br>Inositol phosphate metabolism (hsa00562)<br>Metabolic pathways (hsa01100)	−0,55	2,64	9,24e-03
**C1RL**	Complement C1r subcomponent like	51279	Q9NZP8	-	−0,59	2,13	2,46e-02
**BIN2**	Bridging integrator 2	51411	Q9UBW5	-	−0,61	1,92	3,60e-02
**MCAM**	Melanoma cell adhesion molecule	4162	P43121	-	−0,64	2,01	3,07e-02

**MEGF8**	Multiple EGF like domains 8	1954	Q7Z7M0	Hedgehog signalling pathway (hsa04340)	−0,65	3,37	2,23e-03
**ITIH4**	Inter-alpha-trypsin inhibitor heavy chain 4	3700	Q14624	-	−0,67	2,18	2,26e-02
**LAMP1**	Lysosomal associated membrane protein 1	3916	P11279	Lysosome (hsa04142)<br>Phagosome (hsa04145)<br>Autophagy (hsa04140)	−0,69	3,46	1,90e-03
**F10**	Coagulation factor X	2159	P00742	Complement and coagulation cascades (hsa04610)	−0,74	1,77	4,87e-02
**RAB10**	RAB10, member RAS oncogene family	10890	P61026	Endocytosis (hsa04144)<br>AMPK signalling pathway (hsa04152)	−0,76	2,29	1,82e-02
**CFI**	Complement factor I	3426	P05156	Complement and coagulation cascades (hsa04610)<br>Staphylococcus aureus infection (hsa05150)	−0,81	2,72	7,79e-03
**GPNMB**	Glycoprotein nmb	10457	Q14956	-	−0,81	2,13	2,48e-02
**FCN3**	Ficolin 3	8547	O75636	-	−0,81	1,80	4,63e-02
**ITGB1**	Integrin subunit beta 1	3688	P05556	PI3K-Akt signaling pathway (hsa04151)<br>Focal adhesion (hsa04510)<br>ECM-receptor interaction (hsa04512)	−0,81	3,07	3,87e-03

**ACE**	Angiotensin I converting enzyme	1636	P12821	Renin-angiotensin system (hsa04614)<br>Renin secretion (hsa04924)<br>Coronavirus disease - COVID-19 (hsa05171)	−0,83	3,66	1,25e-03
**LUM**	Lumican	4060	P51884	Proteoglycans in cancer (hsa05205)	−0,84	2,41	1,42e-02
**ADGRG6**	Adhesion G protein-coupled receptor G6	57211	Q86SQ4	-	−0,86	2,35	1,63e-02
**PROS1**	Protein S	5627	P07225	Complement and coagulation cascades (hsa04610)<br>Efferocytosis (hsa04148)	−0,87	2,97	4,74e-03
**C5**	Complement C5	727	P01031	Complement and coagulation cascades (hsa04610)<br>Neutrophil extracellular trap formation (hsa04613)<br>Regulation of actin cytoskeleton (hsa04810)	−0,88	2,91	5,37e-03
**SELL**	Selectin L	6402	P14151	Cell adhesion molecules (hsa04514)	−0,89	2,29	1,85e-02
**SERPINF2**	Serpin family F member 2	5345	P08697	Complement and coagulation cascades (hsa04610)	−0,90	3,69	1,19e-03

**PTPRJ**	Protein tyrosine phosphatase receptor type J	5795	Q12913	Adherens junction (hsa04520)<br>Chemical carcinogenesis - reactive oxygen species (hsa05208)	−0,90	6,08	1,32e-05
**AOC3**	Amine oxidase copper containing 3	8639	Q16853	Glycine, serine and threonine metabolism (hsa00260)<br>Tyrosine metabolism (hsa00350)<br>Phenylalanine metabolism (hsa00360)	−0,90	2,49	1,21e-02
**AZGP1**	Alpha-2-glycoprotein 1, zinc-binding	563	P25311	-	−0,93	3,12	3,53e-03
**C8B**	Complement C8 beta chain	732	P07358	Complement and coagulation cascades (hsa04610)<br>Regulation of actin cytoskeleton (hsa04810)<br>Coronavirus disease – COVID-19 (hsa05171)	−0,93	1,95	3,43e-02
**MASP1**	MBL associated serine protease 1	5648	P48740	Complement and coagulation cascades (hsa04610)<br>Staphylococcus aureus infection (hsa05150)<br>Coronavirus disease – COVID-19 (hsa05171)	−0,93	3,02	4,30e-03

**ENPEP**	Glutamyl aminopeptidase	2028	Q07075	Renin-angiotensin system (hsa04614)	−0,94	1,76	4,94e-02
**CNTN3**	Contactin 3	5067	Q9P232	-	−0,95	3,07	3,87e-03
**C8G**	Complement C8 gamma chain	733	P07360	Complement and coagulation cascades (hsa04610)<br>Regulation of actin cytoskeleton (hsa04810)<br>Coronavirus disease – COVID-19 (hsa05171)	−0,96	3,33	2,38e-03
**COLEC10**	Collectin subfamily member 10	10584	Q9Y6Z7	-	−0,97	1,87	3,97e-02
**SERPINA7**	Serpin family A member 7	6906	P05543	Thyroid hormone synthesis (hsa04918)	−0,98	4,65	1,87e-04
**B4GAT1**	Beta-1,4-glucuronyltransferase 1	11041	O43505	Mannose type O-glycan biosynthesis (hsa00515)<br>Metabolic pathways (hsa01100)	−0,99	5,16	7,10e-05
**F13A1**	Coagulation factor XIII A chain	2162	P00488	Complement and coagulation cascades (hsa04610)<br>Coronavirus disease – COVID-19 (hsa05171)	−0,99	2,88	5,57e-03
**F11**	Coagulation factor XI	2160	P03951	Complement and coagulation cascades (hsa04610)	−1,00	2,12	2,49e-02
**C8A**	Complement C8 alpha chain	731	P07357	Complement and coagulation cascades (hsa04610)	−1,02	3,51	1,69e-03

				Regulation of actin cytoskeleton (hsa04810)<br>Coronavirus disease – COVID-19 (hsa05171)			
**DPP4**	Dipeptidyl peptidase 4	1803	P27487	Virion – Hepatitis viruses (hsa03272)<br>Protein digestion and absorption (hsa04974)	−1,03	5,77	2,41e-05
**MYL12B**	Myosin light chain 12B	103910	O14950	Focal adhesion (hsa04510)<br>Platelet activation (hsa04611)<br>Leukocyte transendothelial migration (hsa04670)	−1,05	2,02	3,00e-02
**B3GNT2**	UDP-GlcNAc:betaGal beta-1,3-N-acetylglucosaminyl-transferase 2	10678	Q9NY97	Glycosaminoglycan biosynthesis – keratan sulfate (hsa00533)<br>Glycosaminoglycan biosynthesis – lacto and neolacto series (hsa00601)<br>Metabolic pathways (hsa01100)	−1,06	3,84	8,93e-04
**MYH9**	Myosin heavy chain 9	4627	P35579	Regulation of actin cytoskeleton (hsa04810)<br>Tight junction (hsa04530)<br>Motor proteins (hsa04814)	−1,07	2,10	2,60e-02
**ENG**	Endoglin	2022	P17813	-	−1,09	3,19	3,08e-03

**PCOLCE**	Procollagen C-endopeptidase enhancer	5118	Q15113	-	−1,10	4,01	6,33e-04
**C3**	Complement C3	718	P01024	Phagosome (hsa04145)<br>Complement and coagulation cascades (hsa04610)<br>Neutrophil extracellular trap formation (hsa04613)	−1,11	4,55	2,25e-04
**C1QB**	Complement C1q B chain	713	P02746	Complement and coagulation cascades (hsa04610)<br>Staphylococcus aureus infection (hsa05150)<br>Efforcytosis (hsa04148)	−1,11	2,68	8,49e-03
**ITGB3**	Integrin subunit beta 3	3690	P05106	PI3K-Akt signaling pathway (hsa04151)<br>Platelet activation (hsa04611)<br>Neutrophil extracellular trap formation (hsa04613)	−1,14	1,83	4,38e-02
**ADAMTS13**	ADAM metallopeptidase with thrombospondin type 1 motif 13	11093	Q76LX8	Platelet activation (hsa04611)	−1,14	3,63	1,32e-03
**CFH**	Complement factor H	3075	P08603	Complement and coagulation cascades (hsa04610)<br>Staphylococcus aureus infection (hsa05150)	−1,16	2,51	1,15e-02

**CHL1**	Cell adhesion molecule L1 like	10752	O00533	-	−1,20	5,05	8,49e-05
**ADIPOQ**	Adiponectin, C1Q and collagen domain containing	9370	Q15848	Adipocytokine signaling pathway (hsa04920)<br>PPAR signaling pathway (hsa03320)<br>AMPK signaling pathway (hsa04152)	−1,20	1,81	4,52e-02
**CDH13**	Cadherin 13	1012	P55290	-	−1,21	8,46	1,45e-07
**CRISP3**	Cysteine rich secretory protein 3	10321	P54108	-	−1,21	2,94	5,05e-03
**ECM1**	Extracellular matrix protein 1	1893	Q16610	-	−1,21	6,12	1,22e-05
**SUN3**	Sad1 and UNC84 domain containing 3	256979	Q8TAQ9	-	−1,25	4,41	2,87e-04
**PI16**	Peptidase inhibitor 16	221476	Q6UXB8	-	−1,25	3,24	2,84e-03
**GC**	GC vitamin D binding protein	2638	P02774	-	−1,26	3,97	6,90e-04
**CPB2**	Carboxypeptidase B2	1361	Q96IY4	Complement and coagulation cascades (hsa04610)<br>Pancreatic secretion (hsa04972)<br>Protein digestion and absorption (hsa04974)	−1,27	5,03	8,76e-05
**SHBG**	Sex hormone binding globulin	6462	P04278	-	−1,27	2,02	3,00e-02
**A1BG**	Alpha-1-B glycoprotein	1	P04217	-	−1,27	5,40	4,53e-05
**CETP**	Cholesteryl ester transfer protein	1071	P11597	Cholesterol metabolism (hsa04979)	−1,27	2,14	2,42e-02

**CNTN1**	Contactin 1	1272	Q12860	Cell adhesion molecules (hsa04514)	−1,28	5,44	4,18e-05
**APOB**	Apolipoprotein B	338	P04114	Fat digestion and absorption (hsa04975)<br>Vitamin digestion and absorption (hsa04977)<br>Cholesterol metabolism (hsa04979)	−1,28	3,82	9,16e-04
**IL1RAP**	Interleukin 1 receptor accessory protein	3556	Q9NPH3	MAPK signaling pathway (hsa04010)<br>Cytokine-cytokine receptor interaction (hsa04060)<br>Th17 cell differentiation (hsa04659)	−1,28	5,12	7,43e-05
**C6**	Complement C6	729	P13671	Complement and coagulation cascades (hsa04610)<br>Regulation of actin cytoskeleton (hsa04810)<br>Coronavirus disease – COVID-19 (hsa05171)	−1,29	5,12	7,43e-05
**ATRN**	Attractin	8455	O75882	-	−1,31	8,10	2,76e-07
**LCAT**	Lecithin-cholesterol acyltransferase	3931	P04180	Glycerophospholipid metabolism (hsa00564)	−1,32	5,71	2,61e-05

				Cholesterol metabolism (hsa04979)			
**TUBB1**	Tubulin beta 1 class VI	81027	Q9H4B7	Phagosome (hsa04145)<br>Motor proteins (hsa04814)<br>Pathways of neurodegeneration - multiple diseases (hsa05022)	−1,33	3,26	2,71e-03
**UMOD**	Uromodulin	7369	P07911	-	−1,33	3,83	8,93e-04
**IGHV3-7**	Immunoglobulin heavy variable 3-7	28452	P01780	-	−1,34	1,94	3,52e-02
**FAP**	Fibroblast activation protein alpha	2191	Q12884	-	−1,35	5,15	7,17e-05
**NCAM1**	Neural cell adhesion molecule 1	4684	P13591	Cell adhesion molecules (hsa04514)<br>Prion disease (hsa05020)<br>Virion - Ebolavirus, Lyssavirus and Morbillivirus (hsa03265)	−1,36	7,42	9,78e-07
**HABP2**	Hyaluronan binding protein 2	3026	Q14520	-	−1,40	3,53	1,64e-03
**APOH**	Apolipoprotein H	350	P02749	Cholesterol metabolism (hsa04979)	−1,40	4,46	2,64e-04
**APOF**	Apolipoprotein F	319	Q13790	-	−1,41	4,53	2,30e-04
**CRTAC1**	Cartilage acidic protein 1	55118	Q9NQ79	-	−1,41	5,67	2,68e-05
**SERPINA6**	Serpin family A member 6	866	P08185	-	−1,44	4,58	2,12e-04

**F2**	Coagulation factor II, prothrombin	2147	P00734	Complement and coagulation cascades (hsa04610)<br>Platelet activation (hsa04611)<br>Phospholipase D signaling pathway (hsa04072)	−1,44	8,56	1,31e-07
**C1QA**	Complement C1q A chain	712	P02745	Complement and coagulation cascades (hsa04610)<br>Efferocytosis (hsa04148)<br>Staphylococcus aureus infection (hsa05150)	−1,46	3,56	1,55e-03
**CLU**	Clusterin	1191	P10909	Complement and coagulation cascades (hsa04610)	−1,46	5,72	2,57e-05
**KNG1**	Kininogen 1	3827	P01042	Complement and coagulation cascades (hsa04610)<br>cGMP-PKG signaling pathway (hsa04022)<br>Sphingolipid signaling pathway (hsa04071)	−1,47	6,93	2,61e-06
**BTD**	Biotinidase	686	P43251	Biotin metabolism (hsa00780)<br>Vitamin digestion and absorption (hsa04977)<br>Metabolic pathways (hsa01100)	−1,48	7,97	3,51e-07
**PLEK**	Pleckstrin	5341	P08567	-	−1,49	2,84	6,10e-03

**GSN**	Gelsolin	2934	P06396	Fc gamma R-mediated phagocytosis (hsa04666)<br>Regulation of actin cytoskeleton (hsa04810)<br>Viral carcinogenesis (hsa05203)	−1,50	8,58	1,31e-07
**CACNA2D1**	Calcium voltage-gated channel auxiliary subunit alpha2delta 1	781	P54289	MAPK signalling pathway (hsa04010)<br>Cardiac muscle contraction (hsa04260)<br>Adrenergic signalling in cardiomyocytes (hsa04261)	−1,56	2,02	3,03e-02
**PON1**	Paraoxonase 1	5444	P27169	-	−1,57	2,89	5,53e-03
**CLEC3B**	C-type lectin domain family 3 member B	7123	P05452	-	−1,58	7,16	1,59e-06
**SERPIND1**	Serpin family D member 1	3053	P05546	Complement and coagulation cascades (hsa04610)	−1,59	5,99	1,55e-05
**ITIH1**	Inter-alpha-trypsin inhibitor heavy chain 1	3697	P19827	-	−1,59	8,93	7,11e-08
**KIT**	KIT proto-oncogene, receptor tyrosine kinase	3815	P10721	PI3K-Akt signaling pathway (hsa04151)<br>MAPK signaling pathway (hsa04010)	−1,64	2,30	1,79e-02

				Phospholipase D signaling pathway (hsa04072)			
**BCHE**	Butyrylcholinesterase	590	P06276	-	−1,72	6,02	1,49e-05
**APOA4**	Apolipoprotein A4	337	P06727	Fat digestion and absorption (hsa04975)<br>Vitamin digestion and absorption (hsa04977)<br>Cholesterol metabolism (hsa04979)	−1,73	5,01	8,92e-05
**KLKB1**	Kallikrein B1	3818	P03952	Complement and coagulation cascades (hsa04610)	−1,73	7,93	3,73e-07
**APOC4**	Apolipoprotein C4	346	P55056	-	−1,73	3,18	3,18e-03
**FETUB**	Fetuin B	26998	Q9UGM5	-	−1,76	5,51	3,72e-05
**SERPINC1**	Serpin family C member 1	462	P01008	Complement and coagulation cascades (hsa04610)	−1,81	6,40	7,27e-06
**PROZ**	Protein Z, vitamin K dependent plasma glycoprotein	8858	P22891	-	−1,82	4,51	2,40e-04
**APOD**	Apolipoprotein D	347	P05090	-	−1,84	7,84	4,21e-07
**RBP4**	Retinol binding protein 4	5950	P02753	-	−1,86	5,13	7,43e-05
**HGFAC**	HGF activator	3083	Q04756	-	−1,88	6,42	7,27e-06
**SELENOP**	Selenoprotein P	6414	P49908	-	−1,90	8,41	1,52e-07
**CNDP1**	Carnosine dipeptidase 1	84735	Q96KN2	Histidine metabolism (hsa00340)	−1,90	4,67	1,81e-04

				Beta-alanine metabolism (hsa00410)<br>Metabolic pathways (hsa01100)			
**APOM**	Apolipoprotein M	55937	O95445	-	−1,91	6,37	7,41e-06
**AHSG**	Alpha-2-HS-glycoprotein	197	P02765	-	−1,92	7,90	3,79e-07
**ITIH2**	Inter-alpha-trypsin inhibitor heavy chain 2	3698	P19823	-	−1,93	10,39	5,43e-09
**DBH**	Dopamine beta-hydroxylase	1621	P09172	Tyrosine metabolism (hsa00350)<br>Metabolic pathways (hsa01100)	−1,97	2,02	3,00e-02
**PCYOX1**	Prenylcysteine oxidase 1	51449	Q9UHG3	Terpenoid backbone biosynthesis (hsa00900)	−2,01	7,56	7,31e-07
**PGLYRP2**	Peptidoglycan recognition protein 2	114770	Q96PD5	-	−2,09	9,42	3,17e-08
**PROC**	Protein C, inactivator of coagulation factors Va and VIIIa	5624	P04070	Complement and coagulation cascades (hsa04610)	−2,10	6,85	2,92e-06
**AFM**	Afamin	173	P43652	-	−2,13	9,79	1,54e-08
**PON3**	Paraoxonase 3	5446	Q15166	-	−2,14	6,25	9,25e-06
**APOC1**	Apolipoprotein C1	341	P02654	Cholesterol metabolism (hsa04979)	−2,16	2,95	4,98e-03
**HRG**	Histidine rich glycoprotein	3273	P04196	-	−2,17	11,18	1,20e-09
**GPLD1**	Glycosylphosphatidylinositol specific phospholipase D1	2822	P80108	Glycosylphosphatidylinositol-anchor biosynthesis (hsa00563)<br>Metabolic pathways (hsa01100)	−2,19	6,87	2,88e-06

**PLG**	plasminogen	5340	P00747	Complement and coagulation cascades (hsa04610)<br>Neuroactive ligand-receptor interaction (hsa04080)<br>Staphylococcus aureus infection (hsa05150)	−2,23	8,64	1,25e-07
**SERPINA4**	Serpin family A member 4	5267	P29622	-	−2,26	12,60	0,00e+00
**IGFBP3**	Insulin like growth factor binding protein 3	3486	P17936	p53 signaling pathway (hsa04115)<br>Cellular senescence (hsa04218)<br>Growth hormone synthesis, secretion and action (hsa04935)	−2,31	6,77	3,42e-06
**IGFALS**	insulin like growth factor binding protein acid labile subunit	3483	P35858	Growth hormone synthesis, secretion and action (hsa04935)	−2,45	6,39	7,27e-06
**F12**	Coagulation factor XII	2161	P00748	Complement and coagulation cascades (hsa04610)	−2,46	5,97	1,56e-05
**SERPINA5**	Serpin family A member 5	5104	P05154	Complement and coagulation cascades (hsa04610)	−2,60	6,40	7,27e-06
**TTR**	Transthyretin	7276	P02766	Thyroid hormone synthesis (hsa04918)	−2,91	6,31	8,39e-06
**SPP2**	Secreted Phosphoprotein 2	6694	Q13103	-	−2,95	4,61	2,03e-04

Table S2. Showing significantly altered protein in the comparison of Septic shock vs. Control. Proteins classified as significantly altered if exhibiting a of log2 fold change > 0.5 or < -0.5 and an FDR adjusted p-value < 0.05. Tables showing gene symbol, gene name, NCBI gene ID, Uniprot ID, related KEGG pathways, log2 fold change, -log10 P-value and adjusted p-value. If more than 3 KEGG pathways associated to the protein the three most relevant were selected for the table.

Table S3 – Acute Pancreatitis versus Control

GENE SYMBOL	GENE NAME	NCBI GENE ID	UNIPROT ID	KEGG PATHWAY	LOG2FC	-LOG10 P-VALUE	ADJUSTED P-VALUE
**CRP**	C-reactive protein	1401	P02741	-	5,09	14,09	0,00e+00
**SAA1**	Serum amyloid A1	6288	P0DJI8	-	4,00	6,77	1,86e-05
**S100A8**	S100 calcium binding protein A8	6279	P05109	IL-17 signaling pathway (hsa04657)<br>Cornified envelope formation (hsa04382)	3,78	6,04	6,70e-05
**S100A12**	S100 calcium binding protein A12	6283	P80511	Cornified envelope formation (hsa04382)	3,64	3,77	3,13e-03
**CHI3L1**	Chitinase 3 like 1	1116	P36222	-	3,61	5,69	1,35e-04
**MMP8**	Matrix metallopeptidase 8	4317	P22894	-	3,31	5,24	2,52e-04
**S100A9**	S100 calcium binding protein A9	6280	P06702	IL-17 signaling pathway (hsa04657)<br>Cornified envelope formation (hsa04382)	3,20	5,22	2,52e-04
**SAA2**	Serum amyloid A2	6289	P0DJI9	-	3,02	3,35	6,37e-03
**CPA1**	Carboxypeptidase A1	1357	P15085	Protein digestion and absorption (hsa04974)<br>Pancreatic secretion (hsa04972)	3,00	4,02	2,42e-03
**LBP**	Lipopolysaccharide binding protein	3929	P18428	NF-kappa B signaling pathway (hsa04064)	2,94	5,17	2,63e-04

				Toll-like receptor signaling pathway (hsa04620)<br>Alcoholic liver disease (hsa04936)			
**REG1B**	Regenerating family member 1 beta	5968	P48304	-	2,88	4,87	4,99e-04
**ORM1**	Orosomucoid 1	5004	P02763	-	2,72	5,40	2,04e-04
**PRSS2**	Serine protease 2	5645	P07478	Protein digestion and absorption (hsa04974)<br>Pancreatic secretion (hsa04972)<br>Neuroactive ligand-receptor interaction (hsa04080)	2,50	2,84	1,59e-02
**IL1RL1**	Interleukin 1 receptor like 1	9173	Q01638	Cytokine-cytokine receptor interaction (hsa04060)	2,44	3,86	2,85e-03
**CAT**	Catalase	847	P04040	Glyoxylate and dicarboxylate metabolism (hsa00630)<br>Carbon metabolism (hsa01200)<br>Peroxisome (hsa04146)	2,32	5,30	2,35e-04
**NUCB1**	Nucleobindin 1	4924	Q02818	-	2,30	3,36	6,34e-03
**MMP9**	Matrix metallopeptidase 9	4318	P14780	IL-17 signaling pathway (hsa04657)<br>TNF signaling pathway (hsa04668)<br>Leukocyte transendothelial migration (hsa04670)	2,22	3,80	3,08e-03

**BPGM**	Bisphosphoglycerate mutase	669	P07738	Glycolysis/Gluconeogenesis (hsa00010)<br>Glycine, serine and threonine metabolism (hsa00260)<br>Metabolic pathways (hsa01100)	1,94	2,60	2,56e-02
**VSIG4**	V-set and immunoglobulin domain containing 4	11326	Q9Y279	Complement and coagulation cascades (hsa04610)	1,93	2,76	1,87e-02
**CDHR2**	Cadherin related family member 2	54825	Q9BYE9	-	1,86	2,58	2,63e-02
**TKT**	Transketolase	7086	P29401	Pentose phosphate pathway (hsa00030)<br>Carbon metabolism (hsa01200)<br>Biosynthesis of amino acids (hsa01230)	1,79	4,66	7,63e-04
**SERPINA3**	Serpin family A member 3	12	P01011	-	1,76	7,26	9,21e-06
**LRG1**	Leucine rich alpha-2-glycoprotein 1	116844	P02750	-	1,72	8,10	2,61e-06
**ORM2**	Orosomucoid 2	5005	P19652	-	1,71	2,52	2,84e-02
**CA1**	Carbonic anhydrase 1	759	P00915	Metabolic pathways (hsa01100)<br>Nitrogen metabolism (hsa00910)	1,65	2,43	3,27e-02
**VWF**	Von Willebrand factor	7450	P04275	PI3K-Akt signaling pathway (hsa04151) Complement and	1,59	3,91	2,82e-03

				coagulation cascades (hsa04610)<br>Platelet activation (hsa04611)			
**APOA5**	Apolipoprotein A5	116519	Q6Q788	PPAR signaling pathway (hsa03320)	1,57	2,43	3,27e-02
**VIM**	Vimentin	7431	P08670	Cytoskeleton in muscle cells (hsa04820)<br>Epstein-Barr virus infection (hsa05169)<br>MicroRNAs in cancer (hsa05206)	1,55	2,28	4,45e-02
**PSMA6**	Proteasome 20S subunit alpha 6	5687	P60900	Proteasome (hsa03050)<br>Prion disease (hsa05020)<br>Pathways of neurodegeneration - multiple diseases (hsa05022)	1,52	2,24	4,73e-02
**LTA4H**	Leukotriene A4 hydrolase	4048	P09960	Arachidonic acid metabolism (hsa00590)<br>Metabolic pathways (hsa01100)	1,52	5,54	1,59e-04
**PSMB7**	Proteasome 20S subunit beta 7	5695	Q99436	Proteasome (hsa03050)<br>Prion disease (hsa05020)<br>Pathways of neurodegeneration - multiple diseases (hsa05022)	1,51	2,21	4,94e-02
**PSMA1**	Proteasome 20S subunit alpha 1	5682	P25786	Proteasome (hsa03050)<br>Prion disease (hsa05020)<br>Pathways of neurodegeneration - multiple diseases (hsa05022)	1,51	2,21	4,94e-02

**NID1**	Nidogen 1	4811	P14543	Cytoskeleton in muscle cells (hsa04820)	1,49	7,30	9,21e-06
**PRDX2**	Peroxiredoxin 2	7001	P32119	-	1,46	2,70	2,10e-02
**TALDO1**	Transaldolase 1	6888	P37837	Pentose phosphate pathway (hsa00030)<br>Carbon metabolism (hsa01200)<br>Biosynthesis of amino acids (hsa01230)	1,41	3,10	1,06e-02
**TIMP1**	TIMP metallopeptidase inhibitor 1	7076	P01033	HIF-1 signaling pathway (hsa04066)	1,41	3,17	9,19e-03
**CTSB**	Cathepsin B	1508	P07858	Lysosome (hsa04142)<br>Apoptosis (hsa04210)<br>Antigen processing and presentation (hsa04612)	1,40	2,95	1,35e-02
**F8**	Coagulation factor VIII	2157	P00451	Complement and coagulation cascades (hsa04610)	1,25	4,26	1,53e-03
**COL6A1**	Collagen type VI alpha 1 chain	1291	P12109	PI3K-Akt signaling pathway (hsa04151)<br>Protein digestion and absorption (hsa04974)<br>Focal adhesion (hsa04510)	1,22	3,04	1,17e-02

**GALNT2**	Polypeptide N-acetylgalactosaminyl-transferase 2	2590	Q10471	Mucin type O-glycan biosynthesis (hsa00512)<br>Other O-glycan biosynthesis (hsa00514)<br>Metabolic pathways (hsa01100)	1,20	3,59	4,46e-03
**EPHB4**	EPH receptor B4	2050	P54760	Axon guidance (hsa04360)	1,12	3,89	2,85e-03
**CTSZ**	Cathepsin Z	1522	Q9UBR2	Lysosome (hsa04142)<br>Apoptosis (hsa04210)	1,11	2,63	2,45e-02
**MAN1A1**	Mannosidase alpha class 1A member 1	4121	P33908	Protein processing in ER (hsa04141)<br>N-glycan biosynthesis (hsa00510)<br>Various types of N-glycan biosynthesis (hsa00531)	1,08	5,56	1,59e-04
**LCP1**	Lymphocyte cytosolic protein 1	3936	P13796	-	1,06	2,45	3,22e-02
**IGFBP4**	Insulin like growth factor binding protein 4	3487	P22692	-	0,97	2,84	1,59e-02
**CTSD**	Cathepsin D	1509	P07339	Sphingolipid signaling pathway (hsa04071)<br>Lysosome (hsa04142)<br>Apoptosis (hsa04210)	0,94	2,24	4,74e-02
**ATRN**	Attractin	8455	O75882	-	−0,71	2,43	3,27e-02
**ABI3BP**	ABI family member 3 binding protein	25890	Q7Z7G0	-	−0,76	2,27	4,46e-02
**ECM1**	Extracellular matrix protein 1	1893	Q16610	-	−0,88	2,92	1,44e-02

**CDH13**	Cadherin 13	1012	P55290	-	−0,90	4,59	8,60e-04
**ITIH1**	Inter-alpha-trypsin inhibitor heavy chain 1	3697	P19827	-	−0,98	3,50	4,84e-03
**GPNMB**	Glycoprotein nmb	10457	Q14956	-	−0,99	2,56	2,66e-02
**BTD**	Biotinidase	686	P43251	Biotin metabolism (hsa00780)<br>Vitamin digestion and absorption (hsa04977)<br>Metabolic pathways (hsa01100)	−0,99	3,49	4,86e-03
**ACE**	Angiotensin I converting enzyme	1636	P12821	Renin-angiotensin system (hsa04614)<br>Renin secretion (hsa04924)<br>Coronavirus disease - COVID-19 (hsa05171)	−1,04	4,48	1,05e-03
**F13A1**	Coagulation factor XIII A chain	2162	P00488	Complement and coagulation cascades (hsa04610)<br>Coronavirus disease - COVID-19 (hsa05171)	−1,04	2,57	2,64e-02
**NCAM1**	Neural cell adhesion molecule 1	4684	P13591	Virion - Ebolavirus, Lyssavirus and Morbillivirus (hsa03265)<br>Cell adhesion molecules (hsa04514)<br>Prion disease (hsa05020)	−1,06	4,18	1,75e-03
**CHL1**	Cell adhesion molecule L1 like	10752	O00533	-	−1,09	3,53	4,78e-03
**SELENOP**	Selenoprotein P	6414	P49908	-	−1,09	2,86	1,58e-02
**CNTN1**	Contactin 1	1272	Q12860	Cell adhesion molecules (hsa04514)	−1,10	3,52	4,80e-03

**SELL**	Selectin L	6402	P14151	Cell adhesion molecules (hsa04514)	−1,11	2,89	1,51e-02
**FAP**	Fibroblast activation protein alpha	2191	Q12884	-	−1,12	3,09	1,06e-02
**AFM**	Afamin	173	P43652	-	−1,12	2,98	1,30e-02
**ITIH2**	Inter-alpha-trypsin inhibitor heavy chain 2	3698	P19823	-	−1,18	4,26	1,53e-03
**AHSG**	Alpha-2-HS-glycoprotein	197	P02765	-	−1,18	2,97	1,32e-02
**CRTAC1**	Cartilage acidic protein 1	55118	Q9NQ79	-	−1,20	3,58	4,46e-03
**APOD**	Apolipoprotein D	347	P05090	-	−1,20	3,29	7,04e-03
**CRISP3**	Cysteine rich secretory protein 3	10321	P54108	-	−1,20	2,36	3,72e-02
**KLKB1**	Kallikrein B1	3818	P03952	Complement and coagulation cascades (hsa04610)	−1,21	3,76	3,13e-03
**CLEC3B**	C-type lectin domain family 3 member B	7123	P05452	-	−1,23	3,99	2,49e-03
**SERPIND1**	Serpin family D member 1	3053	P05546	Complement and coagulation cascades (hsa04610)	−1,36	3,87	2,85e-03
**GPLD1**	Glycosylphosphatidylinositol specific phospholipase D1	2822	P80108	Glycosylphosphatidylinositol-anchor biosynthesis (hsa00563)<br>Metabolic pathways (hsa01100)	−1,37	2,52	2,84e-02
**APOA4**	Apolipoprotein A4	337	P06727	Fat digestion and absorption (hsa04975)	−1,38	2,77	1,85e-02

				Vitamin digestion and absorption (hsa04977)<br>Cholesterol metabolism (hsa04979)			
**PGLYRP2**	Peptidoglycan recognition protein 2	114770	Q96PD5	-	−1,39	4,30	1,52e-03
**GSN**	Gelsolin	2934	P06396	Fc gamma R-mediated phagocytosis (hsa04666)<br>Regulation of actin cytoskeleton (hsa04810)<br>Viral carcinogenesis (hsa05203)	−1,43	6,91	1,63e-05
**IGFBP3**	Insulin like growth factor binding protein 3	3486	P17936	p53 signaling pathway (hsa04115)<br>Cellular senescence (hsa04218)<br>Growth hormone synthesis, secretion and action (hsa04935)	−1,44	2,45	3,22e-02
**ADA2**	Adenosine deaminase 2	51816	Q9NZK5	Purine metabolism (hsa00230)<br>Metabolic pathways (hsa01100)<br>Nucleotide metabolism (hsa01232)	−1,48	2,56	2,67e-02
**HGFAC**	HGF activator	3083	Q04756	-	−1,53	3,81	3,08e-03
**SERPINA4**	Serpin family A member 4	5267	P29622	-	−1,53	6,57	2,53e-05
**FETUB**	Fetuin B	26998	Q9UGM5	-	−1,57	3,78	3,13e-03
**HRG**	Histidine rich glycoprotein	3273	P04196	-	−1,57	6,18	5,50e-05

**CNDP1**	Carnosine dipeptidase 1	84735	Q96KN2	Histidine metabolism (hsa00340)<br>Beta-alanine metabolism (hsa00410)<br>Metabolic pathways (hsa01100)	−1,77	3,43	5,45e-03
**F12**	Coagulation factor XII	2161	P00748	Complement and coagulation cascades (hsa04610)	−2,02	3,56	4,60e-03
**SERPINA5**	Serpin family A member 5	5104	P05154	Complement and coagulation cascades (hsa04610)	−2,15	3,91	2,82e-03

Table S3. Showing significantly altered protein in the comparison of Acute pancreatitis vs. Control. Proteins classified as significantly altered if exhibiting a of log2 fold change > 0.5 or < -0.5 and an FDR adjusted p-value < 0.05. Tables showing gene symbol, gene name, NCBI gene ID, Uniprot ID, related KEGG pathways, log2 fold change, -log10 P-value and adjusted p-value. If more than 3 KEGG pathways associated to the protein the three most relevant were selected for the table.

Table S4 - Septic shock versus Acute pancreatitis

GENE SYMBOL	GENE NAME	NCBI GENE ID	UNIPROT ID	KEGG	LOG2FC	-LOG10 P-VALUE	ADJUSTED P-VALUE
**MARCKS**	Myristoylated alanine rich protein kinase C substrate	4082	P29966	Fc gamma R-mediated phagocytosis (hsa04666)<br>MicroRNAs in cancer (hsa05206)	1,66	3,28	2,30e-02
**HSP90AA1**	heat shock protein 90 alpha family class A member 1	3320	P07900	Protein processing in endoplasmic reticulum (hsa04141)<br>PI3K-Akt signaling pathway (hsa04217)<br>Antigen processing and presentation (hsa04612)	1,57	2,79	3,82e-02
**PSAP**	Prosaposin	5660	P07602	Lysosome (hsa04142)<br>Sphingolipid metabolism (hsa00600)	1,18	2,69	4,64e-02
**CD163**	CD163 molecule	9332	Q86VB7	-	1,14	3,18	2,30e-02
**GANAB**	Glucosidase II alpha subunit	23193	Q14697	Protein processing in endoplasmic reticulum (hsa04141)<br>N-Glycan biosynthesis (hsa00510)<br>Metabolic pathways (hsa01100)	1,09	3,05	2,59e-02
**SERPINB1**	Serpin family B member 1	1992	P30740	-	0,89	3,85	1,32e-02

**COTL1**	Coactosin like protein 1	23406	Q14019	-	0,89	3,26	2,30e-02
**PTPRJ**	Protein tyrosine phosphatase receptor type J	5795	Q12913	Adherens junction (hsa04520)<br>Chemical carcinogenesis - reactive oxygen species (hsa05208)	-0,62	3,19	2,30e-02
**MAN1A1**	Mannosidase alpha class 1A member 1	4121	P33908	Protein processing in endoplasmic reticulum (hsa04141)<br>N-glycan biosynthesis (hsa00510)<br>Various types of N-glycan biosynthesis (hsa00531)	-0,66	2,81	3,77e-02
**F2**	Coagulation factor II, prothrombin	2147	P00734	Complement and coagulation cascades (hsa04610)<br>Platelet activation (hsa04611)<br>Phospholipase D signaling pathway (hsa04072)	-0,77	3,10	2,49e-02
**LRG1**	Leucine rich alpha-2-glycoprotein 1	116844	P02750	-	-0,88	3,21	2,30e-02
**C6**	Complement C6	729	P13671	Complement and coagulation cascades (hsa04610)<br>Regulation of actin cytoskeleton (hsa04810)<br>Coronavirus disease - COVID-19 (hsa05171)	-0,99	3,25	2,30e-02
**AFM**	Afamin	173	P43652	-	-1,01	2,97	2,81e-02

**APOF**	Apolipoprotein F	319	Q13790	-	-1,11	2,92	3,08e-02
**F9**	Coagulation factor IX	2158	P00740	Complement and coagulation cascades (hsa04610)	-1,11	4,43	6,51e-03
**APOH**	Apolipoprotein H	350	P02749	Cholesterol metabolism (hsa04979)	-1,16	3,17	2,30e-02
**PLG**	Plasminogen	5340	P00747	Complement and coagulation cascades (hsa04610)<br>Neuroactive ligand-receptor interaction (hsa04080)<br>Staphylococcus aureus infection (hsa05150)	-1,16	3,00	2,79e-02
**PCYOX1**	Prenylcysteine oxidase 1	51449	Q9UHG3	Terpenoid backbone biosynthesis (hsa00900)	-1,22	3,36	2,30e-02
**SNCA**	Synuclein alpha	6622	P37840	Alzheimer disease (hsa05010)<br>Parkinson disease (hsa05012)<br>Pathways of neurodegeneration - multiple diseases (hsa05022)	-1,28	3,06	2,59e-02
**APOE**	Apolipoprotein E	348	P02649	Cholesterol metabolism (hsa04979)<br>Alzheimer's disease (hsa05010)	-1,37	4,44	6,51e-03
**PROC**	protein C, inactivator of coagulation factors Va and VIIIa	5624	P04070	Complement and coagulation cascades (hsa04610)	-1,41	3,53	2,30e-02
**PRDX2**	Peroxiredoxin 2	7001	P32119	-	-1,42	3,16	2,30e-02
**HABP2**	Hyaluronan binding protein 2	3026	Q14520	-	-1,48	3,91	1,32e-02

**APOA5**	Apolipoprotein A5	116519	Q6Q788	PPAR signaling pathway (hsa03320)	-1,67	3,35	2,30e-02
**APOC2**	Apolipoprotein C2	344	P02655	Cholesterol metabolism (hsa04979)	-1,94	3,42	2,30e-02
**BPGM**	Bisphosphoglycerate mutase	669	P07738	Glycolysis/Gluconeogenesis (hsa00010)<br>Glycine, serine and threonine metabolism (hsa00260)<br>Metabolic pathways (hsa01100)	-2,00	3,36	2,30e-02
**APOC3**	Apolipoprotein C3	345	P02656	PPAR signaling pathway (hsa03320)<br>Cholesterol metabolism (hsa04979)	-2,20	4,12	1,01e-02
**APOC4**	Apolipoprotein C4	346	P55056	-	-2,35	5,41	2,56e-03
**CPA1**	Carboxypeptidase A1	1357	P15085	Protein digestion and absorption (hsa04974)<br>Pancreatic secretion (hsa04972)	-2,85	4,41	6,51e-03

Table S4. Showing significantly altered protein in the comparison of Septic shock vs. Acute pancreatitis. Proteins classified as significantly altered if exhibiting a of log2 fold change > 0.5 or < -0.5 and an FDR adjusted p-value < 0.05. Tables showing gene symbol, gene name, NCBI gene ID, Uniprot ID, related KEGG pathways, log2 fold change, -log10 P-value and adjusted p-value. If more than 3 KEGG pathways associated to the protein the three most relevant were selected for the table.

Figure S1 – Gene Ontology Biological Process

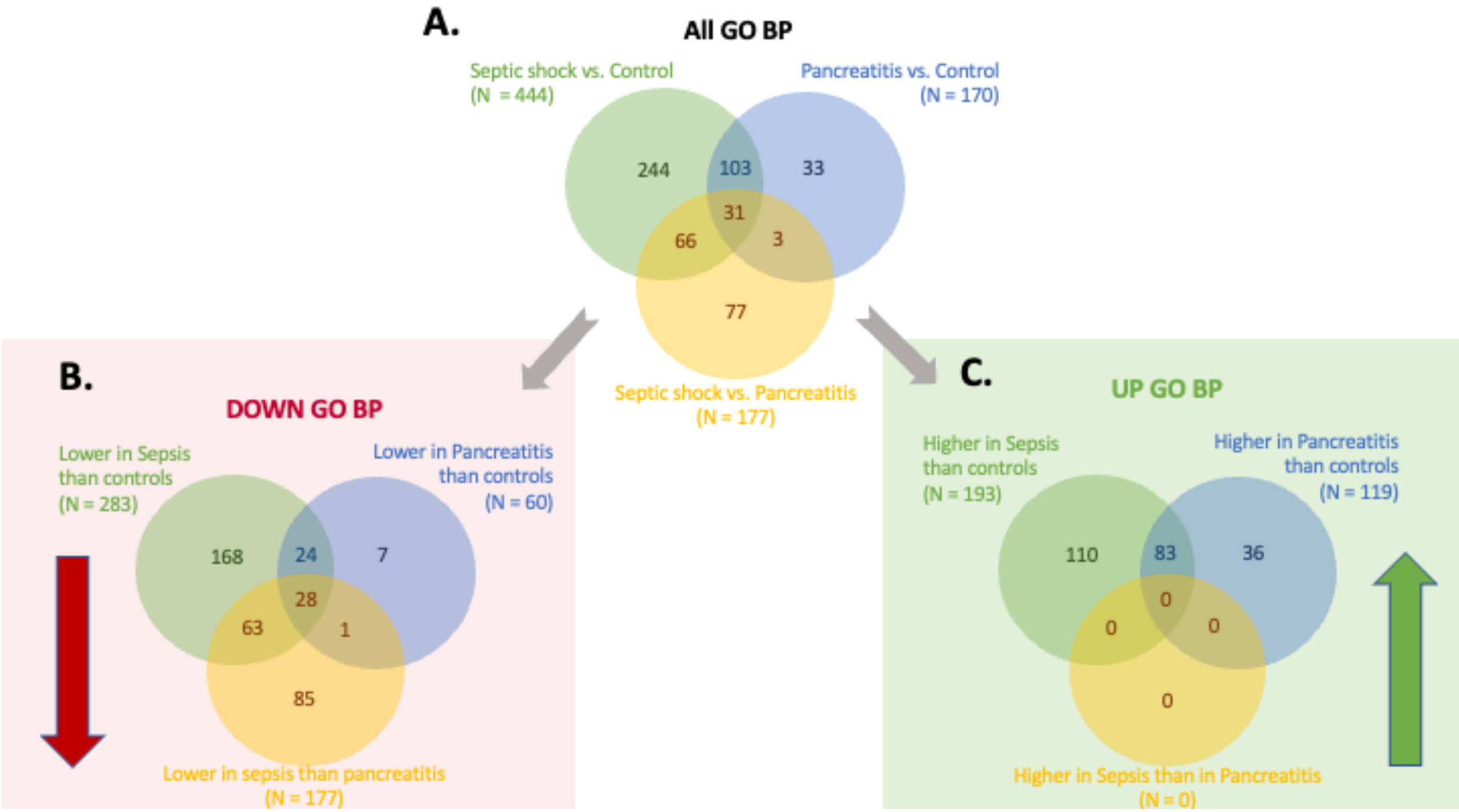

Figure S1. Venn diagrams showing the distribution of Gene Ontology (GO) domain Biological Process (BP) in plain numbers for the comparisons Septic shock vs. Control (green circles), Pancreatitis vs. Control (blue circles) and Septic shock vs. Pancreatitis (yellow circles). Numbers in the overlapping areas representing terms significantly altered in multiple comparisons. **A.** Terms enriched from all significantly altered proteins. **B.** Terms enriched from significantly downregulated proteins. **C.** Terms enriched from significantly upregulated proteins.

Figure S2 – Gene Ontology Cellular Component

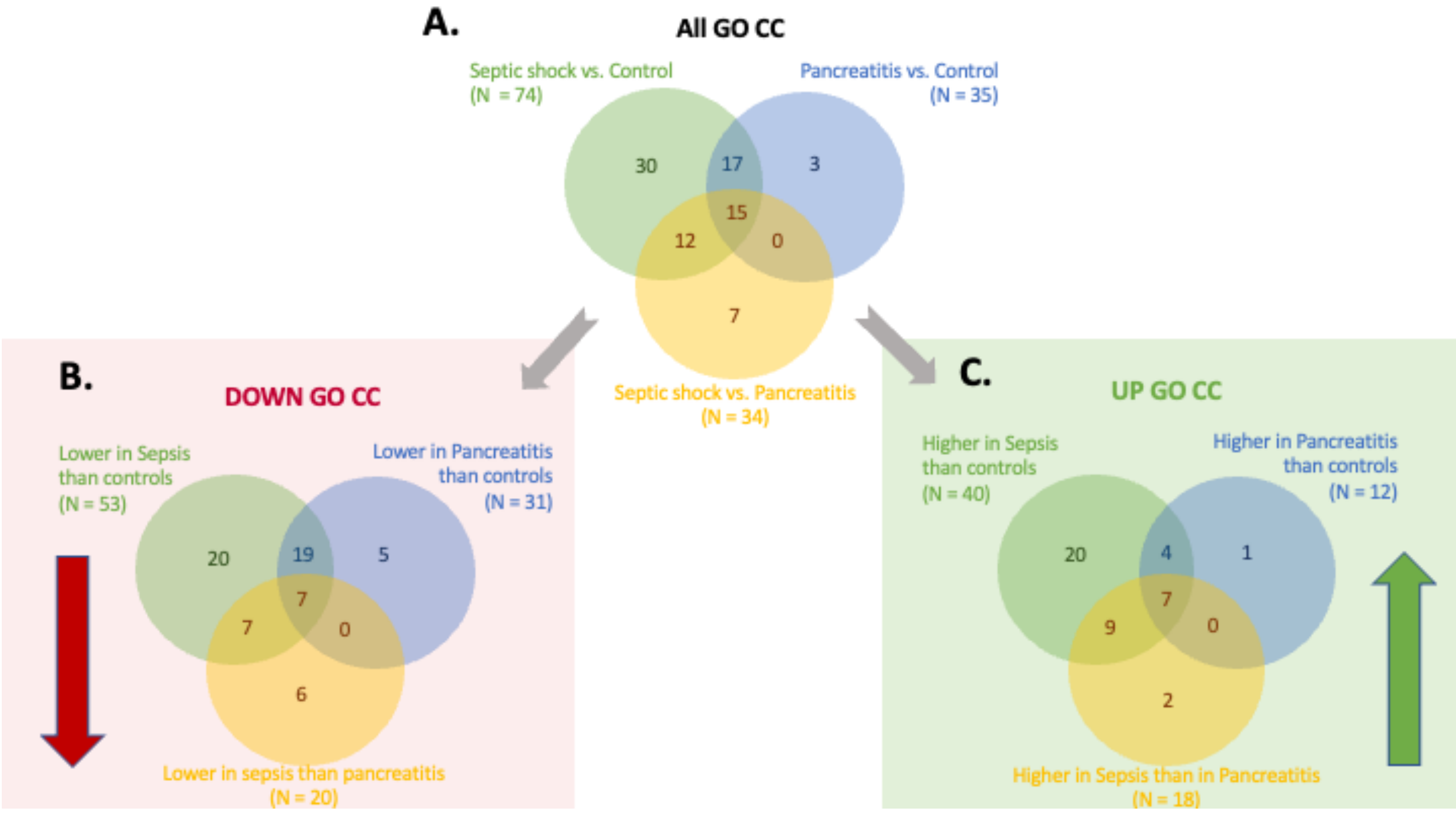

Figure S2. Venn diagrams showing the distribution of Gene Ontology (GO) domain Cellular Component (CC) in plain numbers for the comparisons Septic shock vs. Control (green circles), Pancreatitis vs. Control (blue circles) and Septic shock vs. Pancreatitis (yellow circles). Numbers in the overlapping areas representing terms significantly altered in multiple comparisons. **A.** Terms enriched from all significantly altered proteins. **B.** Terms enriched from significantly downregulated proteins. **C.** Terms enriched from significantly upregulated proteins.

Figure S3 – Gene Ontology Molecular Function

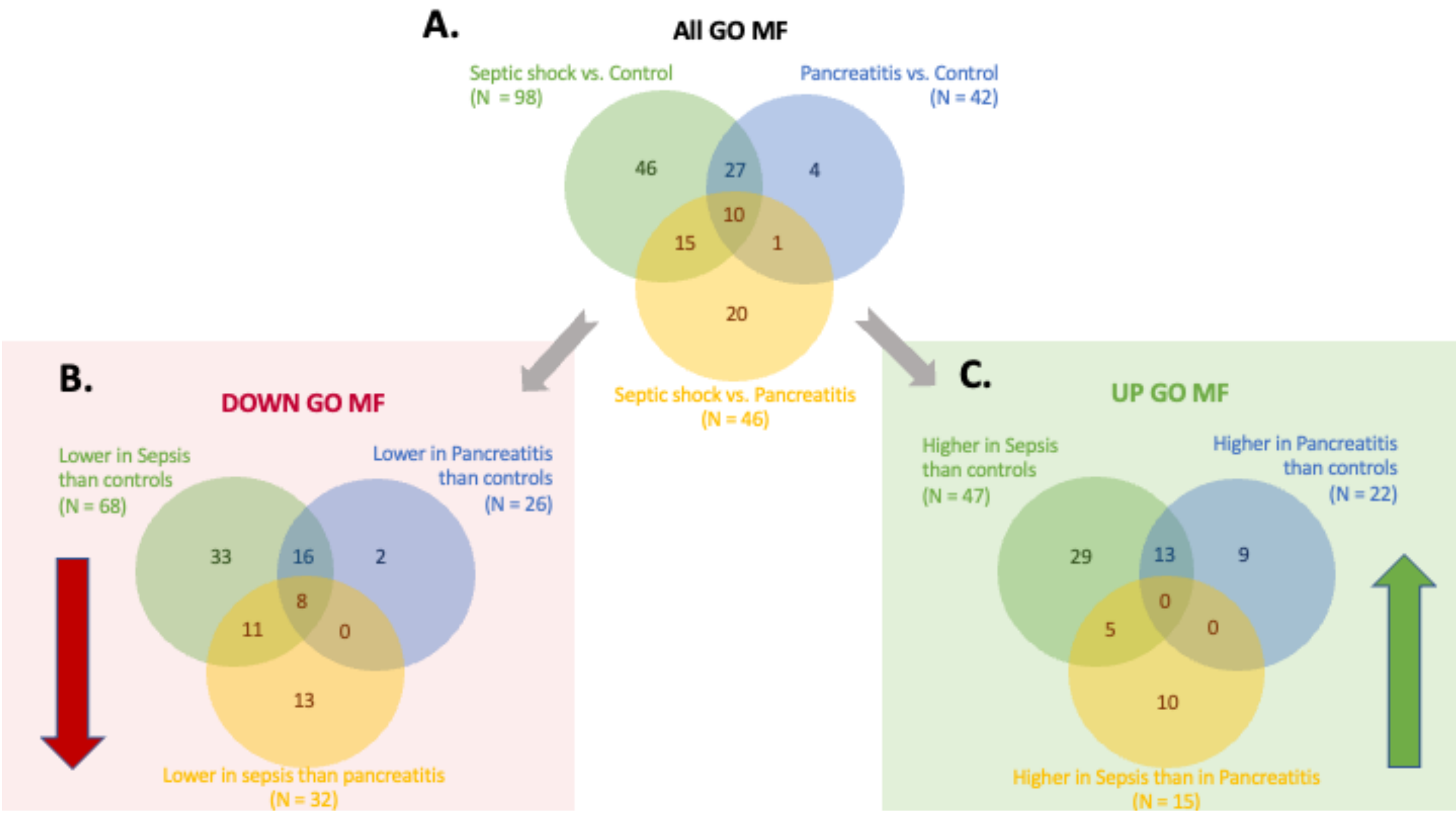

Figure S3. Venn diagrams showing the distribution of Gene Ontology (GO) domain Molecular Function (MF) in plain numbers for the comparisons Septic shock vs. Control (green circles), Pancreatitis vs. Control (blue circles) and Septic shock vs. Pancreatitis (yellow circles). Numbers in the overlapping areas representing terms significantly altered in multiple comparisons. **A.** Terms enriched from all significantly altered proteins. **B.** Terms enriched from significantly downregulated proteins. **C.** Terms enriched from significantly upregulated proteins.

Table S5a – Gene Ontology Sepsis vs Control Up

Description	Ontology	geneID	Protein Count	Adjusted P-value
**acute-phase response**	BP	CD163, CRP, F8, LBP, ORM1, SAA1, SAA2, SERPINA3	8	7,57E-07
**collagen metabolic process**	BP	COL1A1, COL1A2, COL5A1, COL6A1, CST3, CTSB, CTSL, MMP8, MMP9, VIM	10	1,16E-06
**regulation of inflammatory response**	BP	DDT, GRN, IL1RL1, LBP, MMP8, MMP9, PLA2G2A, PSMA1, PSMA6, PSMB4, S100A12, S100A8, S100A9, SAA1, SIRPA, TNC	16	4,66E-06
**extracellular matrix organization**	BP	ADAMTSL2, COL11A2, COL1A1, COL1A2, COL2A1, COL5A1, COL6A1, CST3, LAMB1, LAMC1, LCP1, MMP8, MMP9, NID1	14	5,12E-06
**extracellular structure organization**	BP	ADAMTSL2, COL11A2, COL1A1, COL1A2, COL2A1, COL5A1, COL6A1, CST3, LAMB1, LAMC1, LCP1, MMP8, MMP9, NID1	14	5,12E-06
**external encapsulating structure organization**	BP	ADAMTSL2, COL11A2, COL1A1, COL1A2, COL2A1, COL5A1, COL6A1, CST3, LAMB1, LAMC1, LCP1, MMP8, MMP9, NID1	14	5,12E-06
**regulation of integrin-mediated signaling pathway**	BP	CD177, LAMB1, LAMC1, NID1, TIMP1	5	3,67E-05
**response to lipopolysaccharide**	BP	B2M, COL6A1, IL18BP, LBP, MMP8, MMP9, MPO, MRC1,	13	4,72E-05

		S100A8, SIRPA, SOD2, VCAM1, VIM		
**acute inflammatory response**	BP	CD163, CRP, F8, LBP, ORM1, SAA1, SAA2, SERPINA3	8	6,73E-05
**response to molecule of bacterial origin**	BP	B2M, COL6A1, IL18BP, LBP, MMP8, MMP9, MPO, MRC1, S100A8, SIRPA, SOD2, VCAM1, VIM	13	7,30E-05
**positive regulation of inflammatory response**	BP	DDT, GRN, IL1RL1, LBP, MMP8, PLA2G2A, S100A12, S100A8, S100A9	9	9,74E-05
**regulation of peptidase activity**	BP	CAST, CST3, CTSD, GRN, ITIH3, MMP9, PSMA3, S100A8, S100A9, SERPINA3, SERPINB1, TIMP1	12	0,000175299
**respiratory burst involved in defense response**	BP	GRN, LBP, MPO, S100A9	4	0,000409484
**collagen fibril organization**	BP	COL11A2, COL1A1, COL1A2, COL2A1, COL5A1, COL6A1	6	0,000629281
**collagen catabolic process**	BP	CST3, CTSB, CTSL, MMP8, MMP9	5	0,000918627
**leukocyte cell-cell adhesion**	BP	B2M, CD177, FGL2, ICAM1, LRG1, S100A8, S100A9, SIRPA, SIRPB1, VCAM1, VSIG4, YWHAG	12	0,001169984
**protein folding**	BP	B2M, CALR, GRN, HSP90AA1, HSP90AB1, HSPA4, HSPA8, PDIA4, TRAP1	9	0,001169984
**endoderm development**	BP	COL5A1, COL6A1, LAMB1, LAMC1, MMP8, MMP9	6	0,001414478
**negative regulation of proteolysis**	BP	CAST, CST3, CTSZ, HSP90AB1, IL1R2, ITIH3, MMP9, SERPINA3, SERPINB1, TIMP1	10	0,001414478
**endodermal cell differentiation**	BP	COL5A1, COL6A1, LAMB1, MMP8, MMP9	5	0,001417831

**regulation of endopeptidase activity**	BP	CAST, CTSD, MMP9, PSMA3, S100A8, S100A9, SERPINA3, SERPINB1, TIMP1	9	0,001668323
**respiratory burst involved in inflammatory response**	BP	GRN, LBP, S100A9	3	0,002168082
**glucose 6-phosphate metabolic process**	BP	GPI, PGD, TALDO1, TKT	4	0,002179953
**response to reactive oxygen species**	BP	COL1A1, COL6A1, HSPA8, IL18BP, MPO, SIRPA, SOD2, TRAP1	8	0,002179953
**leukocyte migration**	BP	CALR, CD177, DDT, ICAM1, LBP, S100A12, S100A8, S100A9, SAA1, SIRPA, VCAM1	11	0,002303874
**endoderm formation**	BP	COL5A1, COL6A1, LAMB1, MMP8, MMP9	5	0,002303874
**regulation of macrophage activation**	BP	GRN, IL1RL1, LBP, MMP8, VSIG4	5	0,002303874
**regulation of basement membrane organization**	BP	LAMB1, LAMC1, NID1	3	0,002316807
**regulation of immune effector process**	BP	B2M, CD177, FGL2, GPI, GRN, HSPA8, ICAM1, LBP, S100A9, VSIG4, YWHAG	11	0,002316807
**defense response to bacterium**	BP	B2M, CRP, GRN, LBP, LCN2, MPO, PLA2G2A, S100A12, S100A8, S100A9	10	0,002522087
**extracellular matrix disassembly**	BP	CST3, LAMC1, LCP1, MMP8, MMP9	5	0,002684193
**positive regulation of integrin-mediated signaling pathway**	BP	LAMB1, LAMC1, NID1	3	0,002684193
**neutrophil migration**	BP	CD177, LBP, S100A12, S100A8, S100A9, SAA1	6	0,002684193
**regulation of nitric oxide biosynthetic process**	BP	ASL, HSP90AA1, HSP90AB1, SIRPA, SOD2	5	0,002684193
**regulation of nitric oxide metabolic process**	BP	ASL, HSP90AA1, HSP90AB1, SIRPA, SOD2	5	0,002938872

**integrin-mediated signaling pathway**	BP	CCN2, CD177, LAMB1, LAMC1, NID1, TIMP1	6	0,002938872
**respiratory system development**	BP	ADAMTSL2, BASP1, CCN2, CHI3L1, COL6A1, CTSZ, LTA4H, TNC	8	0,003260604
**antigen processing and presentation**	BP	B2M, CALR, CTSD, CTSL, FGL2, ICAM1	6	0,003720587
**basement membrane organization**	BP	COL6A1, LAMB1, LAMC1, NID1	4	0,003720587
**pyridine nucleotide metabolic process**	BP	ALDOA, COL6A1, GPI, PGD, PGK1, TALDO1, TKT	7	0,003720587
**nicotinamide nucleotide metabolic process**	BP	ALDOA, COL6A1, GPI, PGD, PGK1, TALDO1, TKT	7	0,003720587
**regulation of extracellular matrix organization**	BP	COL6A1, CST3, LAMB1, LAMC1, NID1	5	0,003771437
**respiratory burst**	BP	GRN, LBP, MPO, S100A9	4	0,004277686
**pyridine-containing compound metabolic process**	BP	ALDOA, COL6A1, GPI, PGD, PGK1, TALDO1, TKT	7	0,00429866
**reactive oxygen species metabolic process**	BP	CCN2, CD177, COL6A1, CRP, MPO, SIRPA, SOD2, TRAP1	8	0,004456658
**myeloid leukocyte migration**	BP	CD177, DDT, LBP, S100A12, S100A8, S100A9, SAA1, SIRPA	8	0,004486135
**positive regulation of nitric oxide biosynthetic process**	BP	ASL, HSP90AA1, HSP90AB1, SOD2	4	0,004749526
**myeloid leukocyte activation**	BP	CD177, GRN, IL1RL1, LBP, MMP8, PLA2G2A, S100A12, VSIG4	8	0,00481307
**nitric oxide biosynthetic process**	BP	ASL, HSP90AA1, HSP90AB1, SIRPA, SOD2	5	0,004899976
**positive regulation of nitric oxide metabolic process**	BP	ASL, HSP90AA1, HSP90AB1, SOD2	4	0,004899976
**astrocyte development**	BP	GRN, S100A8, S100A9, VIM	4	0,005260157
**formation of primary germ layer**	BP	COL5A1, COL6A1, GPI, LAMB1, MMP8, MMP9	6	0,005318066
**neutrophil chemotaxis**	BP	LBP, S100A12, S100A8, S100A9, SAA1	5	0,005777895

**negative regulation of peptidase activity**	BP	CAST, CST3, ITIH3, MMP9, SERPINA3, SERPINB1, TIMP1	7	0,00661418
**lung development**	BP	ADAMTSL2, CCN2, CHI3L1, COL6A1, CTSZ, LTA4H, TNC	7	0,00661418
**regulation of respiratory burst**	BP	GRN, LBP, S100A9	3	0,00661418
**nitric oxide metabolic process**	BP	ASL, HSP90AA1, HSP90AB1, SIRPA, SOD2	5	0,006671354
**respiratory tube development**	BP	ADAMTSL2, CCN2, CHI3L1, COL6A1, CTSZ, LTA4H, TNC	7	0,007112649
**reactive nitrogen species metabolic process**	BP	ASL, HSP90AA1, HSP90AB1, SIRPA, SOD2	5	0,007112649
**leukocyte migration involved in inflammatory response**	BP	LBP, S100A8, S100A9	3	0,007112649
**pentose-phosphate shunt**	BP	PGD, TALDO1, TKT	3	0,007112649
**chronic inflammatory response**	BP	S100A8, S100A9, VCAM1	3	0,007935065
**cartilage development**	BP	CCN2, CHI3L1, COL11A2, COL1A1, COL2A1, COL6A1, TIMP1	7	0,007935065
**cellular response to reactive oxygen species**	BP	HSPA8, IL18BP, MPO, SIRPA, SOD2, TRAP1	6	0,007935065
**response to zinc ion**	BP	LTA4H, S100A8, SOD2, VCAM1	4	0,007935065
**response to hydrogen peroxide**	BP	COL1A1, HSPA8, IL18BP, SIRPA, SOD2	5	0,009471128
**NADPH regeneration**	BP	PGD, TALDO1, TKT	3	0,009930045
**granulocyte migration**	BP	CD177, LBP, S100A12, S100A8, S100A9, SAA1	6	0,009961991
**connective tissue development**	BP	CCN2, CHI3L1, COL11A2, COL1A1, COL2A1, COL5A1, COL6A1, TIMP1	8	0,009961991
**chaperone-mediated protein complex assembly**	BP	HSP90AA1, HSP90AB1, HSPA4	3	0,010815285
**cellular response to lipopolysaccharide**	BP	B2M, LBP, MMP8, MMP9, MRC1, SIRPA, VIM	7	0,012135696

**production of molecular mediator involved in inflammatory response**	BP	GRN, IL1R2, LBP, S100A9, SIRPA	5	0,012427428
**proteasome-mediated ubiquitin-dependent protein catabolic process**	BP	HSP90AB1, PSMA1, PSMA3, PSMA4, PSMA5, PSMA6, PSMA7, PSMB2, PSMB3, PSMB4	10	0,013049328
**negative regulation of endopeptidase activity**	BP	CAST, MMP9, SERPINA3, SERPINB1, TIMP1	5	0,013163627
**embryo implantation**	BP	CALR, MMP9, SPP1, TIMP1	4	0,013537971
**protein refolding**	BP	B2M, HSP90AA1, HSPA8	3	0,01421002
**macrophage activation**	BP	GRN, IL1RL1, LBP, MMP8, VSIG4	5	0,014323321
**leukocyte chemotaxis**	BP	CALR, DDT, LBP, S100A12, S100A8, S100A9, SAA1	7	0,014993679
**cellular response to molecule of bacterial origin**	BP	B2M, LBP, MMP8, MMP9, MRC1, SIRPA, VIM	7	0,015549288
**defense response to fungus**	BP	MPO, S100A12, S100A8, S100A9	4	0,0170454
**negative regulation of protein processing**	BP	CAST, CTSZ, IL1R2	3	0,018044959
**positive regulation of macrophage activation**	BP	IL1RL1, LBP, MMP8	3	0,018044959
**cellular response to heat**	BP	HSP90AA1, HSP90AB1, HSPA8, YWHAE	4	0,018343168
**cell chemotaxis**	BP	CALR, DDT, LBP, S100A12, S100A8, S100A9, SAA1, VCAM1	8	0,018343168
**negative regulation of hydrolase activity**	BP	CAST, CST3, ITIH3, MMP9, SERPINA3, SERPINB1, TIMP1	7	0,018343168
**positive regulation of cell adhesion**	BP	B2M, CALR, LAMB1, LAMC1, NID1, SAA1, SIRPA, SIRPB1, VCAM1, YWHAG	10	0,018463635
**antigen processing and presentation of exogenous peptide antigen via MHC class II**	BP	B2M, CTSD, CTSL	3	0,01874889

**antigen processing and presentation of peptide antigen**	BP	B2M, CALR, CTSD, CTSL	4	0,019912519
**carbohydrate derivative catabolic process**	BP	ALDOA, CHI3L1, COL6A1, CST3, CTSL, GPI, PGK1	7	0,019912519
**negative regulation of protein maturation**	BP	CAST, CTSZ, IL1R2	3	0,019912519
**positive regulation of phagocytosis**	BP	CALR, HSPA8, SIRPA, SIRPB1	4	0,020705241
**monosaccharide metabolic process**	BP	ALDOA, GOT1, GPI, GSTO1, IGFBP4, PGD, PGK1	7	0,021269851
**positive regulation of reactive oxygen species metabolic process**	BP	CD177, CRP, SIRPA, SOD2	4	0,021320352
**cellular extravasation**	BP	CD177, ICAM1, SIRPA, VCAM1	4	0,021624455
**response to ethanol**	BP	HSPA8, S100A8, SOD2, TNC, VCAM1	5	0,021624455
**granulocyte chemotaxis**	BP	LBP, S100A12, S100A8, S100A9, SAA1	5	0,021624455
**response to metal ion**	BP	B2M, CALR, GPI, HSPA8, LTA4H, S100A8, SOD2, VCAM1	8	0,021624455
**maintenance of location**	BP	CALR, CRP, GSTO1, NPC2, PLIN3, S100A8, S100A9, YWHAE	8	0,021624455
**interleukin-1 production**	BP	IL1R2, ORM1, SAA1, SERPINB1, SIRPA	5	0,021624455
**regulation of interleukin-1 production**	BP	IL1R2, ORM1, SAA1, SERPINB1, SIRPA	5	0,021624455
**chaperone-mediated protein folding**	BP	HSP90AB1, HSPA8, PDIA4, TRAP1	4	0,021691265
**cellular response to biotic stimulus**	BP	B2M, LBP, MMP8, MMP9, MRC1, SIRPA, VIM	7	0,022333657
**superoxide metabolic process**	BP	CD177, CRP, MPO, SOD2	4	0,022333657
**antigen processing and presentation of peptide antigen via MHC class II**	BP	B2M, CTSD, CTSL	3	0,02244564

**negative regulation of response to external stimulus**	BP	DDT, FGL2, GRN, PSMA1, PSMB4, SAA1, SIRPA, SPP1, VSIG4	9	0,022770751
**response to fungus**	BP	MPO, S100A12, S100A8, S100A9	4	0,022770751
**gastrulation**	BP	COL5A1, COL6A1, GPI, LAMB1, MMP8, MMP9	6	0,024253817
**glial cell development**	BP	COL6A1, GRN, S100A8, S100A9, VIM	5	0,024253817
**antimicrobial humoral immune response mediated by antimicrobial peptide**	BP	B2M, REG1A, REG1B, S100A12, S100A9	5	0,024253817
**antigen processing and presentation of peptide or polysaccharide antigen via MHC class II**	BP	B2M, CTSD, CTSL	3	0,024976538
**response to peptide hormone**	BP	COL1A1, COL6A1, CTSD, GOT1, LTA4H, REG1A, REG1B, TIMP1, YWHAG	9	0,025006603
**cell-substrate adhesion**	BP	CALR, CCN2, COL1A1, LAMB1, LAMC1, NID1, VCAM1, VWF	8	0,025493205
**regulation of toll-like receptor signaling pathway**	BP	S100A8, S100A9, YWHAE	3	0,026267981
**negative regulation of immune response**	BP	FGL2, GRN, IL1RL1, PSMA1, PSMB4, VSIG4	6	0,026267981
**adhesion of symbiont to host cell**	BP	HSP90AB1, ICAM1	2	0,026581499
**positive regulation of muscle cell differentiation**	BP	LAMB1, LAMC1, NID1, SOD2	4	0,027388483
**ossification**	BP	CCN2, COL1A1, COL1A2, COL2A1, COL6A1, RRBP1, SPP1, TNC, VCAN	9	0,027624868
**endothelial cell migration**	BP	CALR, EPHB4, FSTL1, GPI, GRN, S100A12, S100A9	7	0,028005217
**positive regulation of leukocyte activation**	BP	B2M, IL1RL1, LBP, MMP8, SIRPA, SIRPB1, VCAM1, YWHAG	8	0,028493331

**response to axon injury**	BP	GRN, SOD2, SPP1, TNC	4	0,028874521
**antigen processing and presentation of exogenous peptide antigen**	BP	B2M, CTSD, CTSL	3	0,03025745
**regulation of neuroinflammatory response**	BP	GRN, MMP8, MMP9	3	0,03025745
**cellular response to UV-A**	BP	MMP9, TIMP1	2	0,03025745
**negative regulation of defense response**	BP	FGL2, GRN, PSMA1, PSMB4, SAA1, SIRPA, VSIG4	7	0,032351759
**chemotaxis**	BP	CALR, DDT, LBP, S100A12, S100A8, S100A9, SAA1, SEMA4B, VCAM1	9	0,03249319
**astrocyte differentiation**	BP	GRN, S100A8, S100A9, VIM	4	0,03249319
**taxis**	BP	CALR, DDT, LBP, S100A12, S100A8, S100A9, SAA1, SEMA4B, VCAM1	9	0,03310743
**response to mechanical stimulus**	BP	CHI3L1, COL1A1, COL6A1, GPI, MPO, TNC	6	0,033302001
**regulation of reactive oxygen species metabolic process**	BP	CD177, CRP, SIRPA, SOD2, TRAP1	5	0,033701983
**glyceraldehyde-3-phosphate metabolic process**	BP	TALDO1, TKT	2	0,033701983
**skin morphogenesis**	BP	COL1A1, COL1A2	2	0,033701983
**cellular response to thyroid hormone stimulus**	BP	CTSB, CTSL	2	0,033701983
**neutrophil activation**	BP	CD177, GRN, PLA2G2A	3	0,034162982
**response to vitamin**	BP	COL1A1, SOD2, SPP1, TNC	4	0,034439929
**positive regulation of cell activation**	BP	B2M, IL1RL1, LBP, MMP8, SIRPA, SIRPB1, VCAM1, YWHAG	8	0,034809022
**response to testosterone**	BP	CALR, GPI, SPP1	3	0,035125287
**superoxide anion generation**	BP	CD177, CRP, SOD2	3	0,035125287
**lung epithelium development**	BP	ADAMTSL2, COL6A1, LTA4H	3	0,035125287
**humoral immune response**	BP	B2M, GPI, REG1A, REG1B, S100A12, S100A9, VSIG4	7	0,035498025
**NADP metabolic process**	BP	PGD, TALDO1, TKT	3	0,035498025

**glycolytic process**	BP	ALDOA, COL6A1, GPI, PGK1	4	0,035498025
**monosaccharide biosynthetic process**	BP	GOT1, GPI, PGD, PGK1	4	0,035498025
**mononuclear cell migration**	BP	CALR, DDT, ICAM1, S100A12, SAA1, SIRPA	6	0,035498025
**fructose 6-phosphate metabolic process**	BP	GPI, TALDO1	2	0,035498025
**adhesion of symbiont to host**	BP	HSP90AB1, ICAM1	2	0,035498025
**leukocyte aggregation**	BP	S100A8, S100A9	2	0,035498025
**positive regulation of endocytosis**	BP	B2M, CALR, HSPA8, SIRPA, SIRPB1	5	0,035498025
**generation of precursor metabolites and energy**	BP	ALDOA, COL6A1, GPI, PGD, PGK1, SOD2, TALDO1, TKT, TRAP1	9	0,035498025
**ADP catabolic process**	BP	ALDOA, COL6A1, GPI, PGK1	4	0,037459797
**regulation of apoptotic signaling pathway**	BP	COL2A1, CTSL, ICAM1, MMP9, S100A8, S100A9, SOD2, TRAP1	8	0,037712419
**protein tetramerization**	BP	ALDOA, B2M, COL6A1, SOD2	4	0,037948091
**cellular oxidant detoxification**	BP	GSTO1, MPO, S100A9, SOD2	4	0,037948091
**response to oxidative stress**	BP	COL1A1, COL6A1, HSPA8, IL18BP, MPO, SIRPA, SOD2, TRAP1	8	0,037948091
**pyridine nucleotide catabolic process**	BP	ALDOA, COL6A1, GPI, PGK1	4	0,037948091
**T cell activation via T cell receptor contact with antigen bound to MHC molecule on antigen presenting cell**	BP	FGL2, ICAM1	2	0,037948091
**outer mitochondrial membrane organization**	BP	HSP90AA1, HSPA4	2	0,037948091
**protein insertion into mitochondrial outer membrane**	BP	HSP90AA1, HSPA4	2	0,037948091
**positive regulation of striated muscle contraction**	BP	GSTO1, HSP90AA1	2	0,037948091

**actin crosslink formation**	BP	LCP1, MARCKS	2	0,037948091
**response to UV-A**	BP	MMP9, TIMP1	2	0,037948091
**phagocytosis**	BP	CALR, CRP, HSPA8, LBP, SIRPA, SIRPB1	6	0,037948091
**purine nucleoside diphosphate catabolic process**	BP	ALDOA, COL6A1, GPI, PGK1	4	0,037948091
**purine ribonucleoside diphosphate catabolic process**	BP	ALDOA, COL6A1, GPI, PGK1	4	0,037948091
**muscle cell apoptotic process**	BP	COL6A1, HSP90AA1, HSPA8, SOD2	4	0,037948091
**regulation of phagocytosis**	BP	CALR, HSPA8, SIRPA, SIRPB1	4	0,038995289
**regulation of cysteine-type endopeptidase activity**	BP	CAST, CTSD, MMP9, S100A8, S100A9	5	0,038995289
**antigen processing and presentation of exogenous antigen**	BP	B2M, CTSD, CTSL	3	0,039237856
**response to nutrient**	BP	COL1A1, SOD2, SPP1, TNC, VCAM1	5	0,039517214
**pyridine-containing compound catabolic process**	BP	ALDOA, COL6A1, GPI, PGK1	4	0,039517214
**positive regulation of response to biotic stimulus**	BP	GRN, HSP90AA1, HSPA8, LBP, S100A8, S100A9, YWHAE, YWHAG	8	0,039746496
**ribonucleoside diphosphate catabolic process**	BP	ALDOA, COL6A1, GPI, PGK1	4	0,039746496
**ADP metabolic process**	BP	ALDOA, COL6A1, GPI, PGK1	4	0,039746496
**response to cadmium ion**	BP	GPI, HSPA8, SOD2	3	0,039746496
**protein nitrosylation**	BP	S100A8, S100A9	2	0,039746496
**peptidyl-cysteine S-nitrosylation**	BP	S100A8, S100A9	2	0,039746496
**chaperone-mediated autophagy**	BP	HSP90AA1, HSPA8	2	0,039746496
**collagen biosynthetic process**	BP	COL1A1, COL5A1, VIM	3	0,04115296
**granulocyte activation**	BP	CD177, GRN, PLA2G2A	3	0,04115296
**response to heat**	BP	HSP90AA1, HSP90AB1, HSPA8, YWHAE	4	0,041354636

**cellular response to oxidative stress**	BP	HSPA8, IL18BP, MPO, SIRPA, SOD2, TRAP1	6	0,043281215
**nucleoside diphosphate catabolic process**	BP	ALDOA, COL6A1, GPI, PGK1	4	0,043281215
**positive regulation of tumor necrosis factor production**	BP	DDT, LBP, MMP8, ORM1	4	0,043281215
**low-density lipoprotein particle remodeling**	BP	MPO, PLA2G2A	2	0,043281215
**regulation of receptor binding**	BP	B2M, MMP9	2	0,043281215
**axon regeneration**	BP	GRN, SPP1, TNC	3	0,044059946
**lung morphogenesis**	BP	COL6A1, CTSZ, TNC	3	0,046152521
**carbohydrate catabolic process**	BP	ALDOA, COL6A1, GPI, PGD, PGK1	5	0,047284739
**opsonization**	BP	CRP, LBP	2	0,047284739
**negative regulation of macrophage activation**	BP	GRN, VSIG4	2	0,047284739
**positive regulation of tumor necrosis factor superfamily cytokine production**	BP	DDT, LBP, MMP8, ORM1	4	0,047284739
**response to amyloid-beta**	BP	ICAM1, MMP9, VCAM1	3	0,047284739
**purine nucleotide metabolic process**	BP	ALDOA, COL6A1, GPI, HSPA8, PGD, PGK1, TALDO1, TKT	8	0,049841848
**osteoblast differentiation**	BP	COL1A1, COL6A1, RRBP1, SPP1, TNC, VCAN	6	0,049841848
**collagen-containing extracellular matrix**	CC	CALR, CCN2, COL11A2, COL1A1, COL1A2, COL2A1, COL5A1, COL6A1, CTSB, CTSD, CTSL, CTSZ, FGL2, HSP90AA1, ICAM1, LAMB1, LAMC1, LGALS3BP, LTBP2, MMP8, MMP9, NID1, ORM1, PSAP, S100A8, S100A9, SERPINA3, SERPINB1, TIMP1, TNC, VCAN, VWF	32	9,46E-25

**secretory granule lumen**	CC	ALDOA, B2M, CHI3L1, CTSD, CTSZ, F8, GPI, GRN, HSP90AA1, HSP90AB1, HSPA8, ITIH3, LCN2, LGALS3BP, LRG1, MMP8, MPO, NPC2, ORM1, PSMA5, S100A12, S100A8, S100A9, SERPINA3, SERPINB1, TIMP1, VWF	27	3,45E-22
**cytoplasmic vesicle lumen**	CC	ALDOA, B2M, CHI3L1, CTSD, CTSZ, F8, GPI, GRN, HSP90AA1, HSP90AB1, HSPA8, ITIH3, LCN2, LGALS3BP, LRG1, MMP8, MPO, NPC2, ORM1, PSMA5, S100A12, S100A8, S100A9, SERPINA3, SERPINB1, TIMP1, VWF	27	3,45E-22
**vesicle lumen**	CC	ALDOA, B2M, CHI3L1, CTSD, CTSZ, F8, GPI, GRN, HSP90AA1, HSP90AB1, HSPA8, ITIH3, LCN2, LGALS3BP, LRG1, MMP8, MPO, NPC2, ORM1, PSMA5, S100A12, S100A8, S100A9, SERPINA3, SERPINB1, TIMP1, VWF	27	3,45E-22
**endoplasmic reticulum lumen**	CC	B2M, CALR, COL11A2, COL1A1, COL1A2, COL2A1, COL5A1, COL6A1, CST3, CTSZ, F8, FSTL1, GANAB, GOLM1, IGFBP4, LAMB1, LAMC1, NUCB1, PDIA4, PRKCSH, SPP1, TIMP1, TNC, VCAN	24	5,87E-19
**proteasome core complex**	CC	PSMA1, PSMA3, PSMA4, PSMA5, PSMA6, PSMA7, PSMB2, PSMB3, PSMB4	9	2,49E-14

**ficolin-1-rich granule lumen**	CC	ALDOA, CST3, CTSB, CTSD, CTSZ, FGL2, GPI, HSP90AA1, HSP90AB1, HSPA8, LRG1, LTA4H, MMP9, PSMA5	14	3,05E-13
**ficolin-1-rich granule**	CC	ALDOA, CST3, CTSB, CTSD, CTSZ, FGL2, GPI, HSP90AA1, HSP90AB1, HSPA8, LRG1, LTA4H, MMP9, PSMA5, SIRPA	15	4,01E-12
**tertiary granule lumen**	CC	ALDOA, B2M, CST3, CTSD, LRG1, LTA4H, MMP8, MMP9, ORM1	9	5,33E-10
**proteasome complex**	CC	PSMA1, PSMA3, PSMA4, PSMA5, PSMA6, PSMA7, PSMB2, PSMB3, PSMB4	9	1,99E-09
**specific granule lumen**	CC	B2M, CHI3L1, CTSD, CTSZ, LCN2, LRG1, MMP8, ORM1	8	4,20E-08
**tertiary granule**	CC	ALDOA, B2M, CD177, CST3, CTSD, LRG1, LTA4H, MMP8, MMP9, ORM1, SIRPA	11	4,20E-08
**fibrillar collagen trimer**	CC	COL11A2, COL1A1, COL1A2, COL2A1, COL5A1	5	6,33E-08
**banded collagen fibril**	CC	COL11A2, COL1A1, COL1A2, COL2A1, COL5A1	5	6,33E-08
**endopeptidase complex**	CC	PSMA1, PSMA3, PSMA4, PSMA5, PSMA6, PSMA7, PSMB2, PSMB3, PSMB4	9	6,47E-08
**vacuolar lumen**	CC	CTSB, CTSD, CTSL, GRN, HSP90AA1, HSPA8, MPO, NPC2, PSAP, SERPINA3, VCAN	11	7,46E-08
**peptidase complex**	CC	PSMA1, PSMA3, PSMA4, PSMA5, PSMA6, PSMA7, PSMB2, PSMB3, PSMB4	9	6,21E-07
**basement membrane**	CC	COL2A1, COL5A1, COL6A1, LAMB1, LAMC1, NID1, TIMP1, TNC	8	6,21E-07

**lysosomal lumen**	CC	CTSB, CTSD, CTSL, HSP90AA1, HSPA8, NPC2, PSAP, VCAN	8	9,67E-07
**complex of collagen trimers**	CC	COL11A2, COL1A1, COL1A2, COL2A1, COL5A1	5	1,09E-06
**endocytic vesicle lumen**	CC	CALR, CTSL, HSP90AA1, MPO, SAA1	5	1,71E-06
**melanosome**	CC	CTSB, CTSD, GANAB, HSP90AA1, HSP90AB1, HSPA8, PDIA4, YWHAE	8	2,26E-06
**pigment granule**	CC	CTSB, CTSD, GANAB, HSP90AA1, HSP90AB1, HSPA8, PDIA4, YWHAE	8	2,26E-06
**specific granule**	CC	B2M, CD177, CHI3L1, CTSD, CTSZ, LCN2, LRG1, MMP8, ORM1	9	2,89E-06
**platelet alpha granule lumen**	CC	ALDOA, F8, ORM1, SERPINA3, TIMP1, VWF	6	1,83E-05
**protein complex involved in cell-matrix adhesion**	CC	LAMB1, LAMC1, NID1, TNC	4	1,83E-05
**collagen trimer**	CC	COL11A2, COL1A1, COL1A2, COL2A1, COL5A1, COL6A1	6	7,53E-05
**platelet alpha granule**	CC	ALDOA, F8, ORM1, SERPINA3, TIMP1, VWF	6	0,000100624
**proteasome core complex, beta-subunit complex**	CC	PSMB2, PSMB3, PSMB4	3	0,000210441
**focal adhesion**	CC	B2M, CALR, HSPA8, ICAM1, IL1RL1, LCP1, MARCKS, TNC, VIM, YWHAE, YWHAG	11	0,000210441
**cell-substrate junction**	CC	B2M, CALR, HSPA8, ICAM1, IL1RL1, LCP1, MARCKS, TNC, VIM, YWHAE, YWHAG	11	0,00025183
**endosome lumen**	CC	B2M, CTSB, CTSD, CTSL	4	0,000420189
**protein complex involved in cell adhesion**	CC	LAMB1, LAMC1, NID1, TNC	4	0,002146759

**endocytic vesicle**	CC	B2M, CALR, CD163, CTSL, HSP90AA1, MPO, SAA1, VIM	8	0,006087241
**perisynaptic extracellular matrix**	CC	TNC, VCAN	2	0,010477486
**azurophil granule lumen**	CC	GRN, MPO, NPC2, SERPINA3	4	0,010744609
**primary lysosome**	CC	GRN, MPO, NPC2, PSAP, SERPINA3	5	0,010821846
**azurophil granule**	CC	GRN, MPO, NPC2, PSAP, SERPINA3	5	0,010821846
**synapse-associated extracellular matrix**	CC	TNC, VCAN	2	0,011241772
**dendrite terminus**	CC	HSP90AA1, HSP90AB1	2	0,012905764
**platelet dense granule lumen**	CC	ITIH3, LGALS3BP	2	0,014286821
**cytolytic granule**	CC	CALR, SERPINB1	2	0,014286821
**endoplasmic reticulum protein-containing complex**	CC	B2M, CALR, GANAB, PRKCSH	4	0,021635957
**platelet dense granule**	CC	ITIH3, LGALS3BP	2	0,030060211
**nuclear matrix**	CC	BASP1, LMNB1, PSMA6, VIM	4	0,030060211
**endoplasmic reticulum-Golgi intermediate compartment**	CC	CALR, CTSZ, F8, NUCB1	4	0,034446994
**external side of plasma membrane**	CC	B2M, CALR, CD163, CTSB, ICAM1, IL1RL1, VCAM1	7	0,039110243
**blood microparticle**	CC	HSPA8, LGALS3BP, ORM1, SERPINA3	4	0,041233789
**phagocytic vesicle**	CC	B2M, CALR, MPO, VIM	4	0,041360625
**high-density lipoprotein particle**	CC	SAA1, SAA2	2	0,044123642
**endolysosome**	CC	CTSB, CTSL	2	0,045678155
**endoplasmic reticulum-Golgi intermediate compartment membrane**	CC	CALR, CTSZ, F8	3	0,045678155
**nuclear periphery**	CC	BASP1, LMNB1, PSMA6, VIM	4	0,04796847

**extracellular matrix structural constituent**	MF	CHI3L1, COL11A2, COL1A1, COL1A2, COL2A1, COL5A1, COL6A1, FGL2, LAMB1, LAMC1, LTBP2, NID1, TNC, VCAN, VWF	15	1,99E-11
**platelet-derived growth factor binding**	MF	COL1A1, COL1A2, COL2A1, COL5A1, COL6A1	5	4,45E-07
**integrin binding**	MF	CALR, CCN2, CD177, COL5A1, ICAM1, LCP1, SPP1, TNC, VCAM1, VWF	10	4,37E-06
**proteoglycan binding**	MF	COL2A1, COL5A1, CTSB, CTSL, NID1, TNC	6	5,71E-06
**extracellular matrix structural constituent conferring tensile strength**	MF	COL11A2, COL1A1, COL1A2, COL2A1, COL5A1, COL6A1	6	1,54E-05
**MHC class II protein complex binding**	MF	B2M, HSP90AA1, HSP90AB1, HSPA8, YWHAE	5	2,40E-05
**carbohydrate binding**	MF	ALDOA, CALR, CHI3L1, GALNT2, GANAB, GPI, MRC1, REG1A, REG1B, TALDO1, VCAN	11	4,00E-05
**growth factor binding**	MF	CCN2, COL1A1, COL1A2, COL2A1, COL5A1, COL6A1, IGFBP4, LTBP2	8	4,59E-05
**glycosaminoglycan binding**	MF	CCN2, COL11A2, COL5A1, FSTL1, LTBP2, MPO, REG1A, REG1B, SAA1, VCAN	10	7,28E-05
**MHC protein complex binding**	MF	B2M, HSP90AA1, HSP90AB1, HSPA8, YWHAE	5	7,42E-05
**collagen binding**	MF	COL6A1, CTSB, CTSL, MMP9, NID1, VWF	6	9,16E-05
**ATP-dependent protein folding chaperone**	MF	HSP90AA1, HSP90AB1, HSPA4, HSPA8, TRAP1	5	9,20E-05
**protein folding chaperone**	MF	CALR, HSP90AA1, HSP90AB1, HSPA4, HSPA8, TRAP1	6	0,000126419

**RAGE receptor binding**	MF	S100A12, S100A8, S100A9	3	0,000660987
**scaffold protein binding**	MF	CD163, HSP90AA1, PSAP, VIM, YWHAE	5	0,0011722
**heparin binding**	MF	CCN2, COL11A2, COL5A1, FSTL1, LTBP2, MPO, SAA1	7	0,001628295
**protease binding**	MF	CD177, COL1A1, COL1A2, CST3, PSAP, VWF	6	0,002801831
**opsonin binding**	MF	CALR, CRP, VSIG4	3	0,003957337
**sulfur compound binding**	MF	CCN2, COL11A2, COL5A1, FSTL1, LTBP2, MPO, SAA1, TKT	8	0,003957337
**molecular sequestering activity**	MF	CALR, LCN2, YWHAE, YWHAG	4	0,00427553
**complement binding**	MF	CALR, CRP, VSIG4	3	0,006500552
**endopeptidase inhibitor activity**	MF	CAST, CST3, ITIH3, SERPINA3, SERPINB1, TIMP1	6	0,006608907
**peptidase regulator activity**	MF	CAST, CST3, CTSL, ITIH3, SERPINA3, SERPINB1, TIMP1	7	0,006608907
**extracellular matrix binding**	MF	ADAMTSL2, LTBP2, NID1, SPP1	4	0,007249274
**peptidase inhibitor activity**	MF	CAST, CST3, ITIH3, SERPINA3, SERPINB1, TIMP1	6	0,007559764
**unfolded protein binding**	MF	CALR, HSP90AA1, HSP90AB1, HSPA8, TRAP1	5	0,009036921
**endopeptidase regulator activity**	MF	CAST, CST3, ITIH3, SERPINA3, SERPINB1, TIMP1	6	0,010130214
**monosaccharide binding**	MF	ALDOA, GPI, MRC1, TALDO1	4	0,010130214
**calcium-dependent protein binding**	MF	CD177, S100A12, S100A8, S100A9	4	0,012480842
**cysteine-type endopeptidase activity**	MF	CTSB, CTSD, CTSL, CTSZ	4	0,015478668
**disulfide oxidoreductase activity**	MF	GSTO1, PDIA4, PGK1	3	0,015478668

**antioxidant activity**	MF	GSTO1, MPO, S100A9, SOD2	4	0,015478668
**lipopolysaccharide binding**	MF	LBP, PSMA1, PSMB4	3	0,015882203
**ubiquitin protein ligase binding**	MF	CALR, GPI, HSP90AA1, HSP90AB1, HSPA8, PSMA3, YWHAE	7	0,018657237
**Toll-like receptor binding**	MF	S100A8, S100A9	2	0,020924335
**ubiquitin-like protein ligase binding**	MF	CALR, GPI, HSP90AA1, HSP90AB1, HSPA8, PSMA3, YWHAE	7	0,025176116
**protein kinase C binding**	MF	MARCKS, PRKCSH, YWHAG	3	0,027253845
**carbon-carbon lyase activity**	MF	ALDOA, DDT, GOT1	3	0,027253845
**intramolecular oxidoreductase activity**	MF	DDT, GPI, PDIA4	3	0,028025955
**cadherin binding**	MF	ALDOA, ARHGAP1, CAST, HSP90AB1, HSPA8, PLIN3, YWHAE	7	0,02814708
**oxidoreductase activity, acting on a sulfur group of donors**	MF	GSTO1, PDIA4, PGK1	3	0,029232093
**long-chain fatty acid binding**	MF	S100A8, S100A9	2	0,029232093
**oligosaccharide binding**	MF	REG1A, REG1B	2	0,029232093
**insulin-like growth factor binding**	MF	CCN2, IGFBP4	2	0,034846567
**peptidoglycan binding**	MF	REG1A, REG1B	2	0,034846567
**DNA polymerase binding**	MF	HSP90AA1, HSP90AB1	2	0,041157181
**cargo receptor activity**	MF	CD163, LGALS3BP, MRC1	3	0,041157181

Table S5a. Showing enriched gene ontologies from upregulated DEPs in sepsis compared to control. BP=biological processes, CC=cellular compartments, MF=molecular functions. Ontologies classified as significantly altered if exhibiting an adjusted p-value < 0.05.

Table S5b – Gene Ontology Sepsis vs Control Down

Description	Ontology	geneID	Protein Count	Adjusted P-value
**blood coagulation**	BP	ADAMTS13, APOH, CPB2, F10, F11, F12, F13A1, F2, FAP, HGFAC, HRG, ITGB3, KLKB1, KNG1, MYH9, PLEK, PLG, PROC, PROS1, PROZ, PTPRJ, SERPINC1, SERPIND1, SERPINF2, TUBB1	25	4,82E-21
**hemostasis**	BP	ADAMTS13, APOH, CPB2, F10, F11, F12, F13A1, F2, FAP, HGFAC, HRG, ITGB3, KLKB1, KNG1, MYH9, PLEK, PLG, PROC, PROS1, PROZ, PTPRJ, SERPINC1, SERPIND1, SERPINF2, TUBB1	25	4,82E-21

**coagulation**	BP	ADAMTS13, APOH, CPB2, F10, F11, F12, F13A1, F2, FAP, HGFAC, HRG, ITGB3, KLKB1, KNG1, MYH9, PLEK, PLG, PROC, PROS1, PROZ, PTPRJ, SERPINC1, SERPIND1, SERPINF2, TUBB1	25	4,82E-21
**wound healing**	BP	ADAMTS13, APOH, CPB2, ENG, F10, F11, F12, F13A1, F2, FAP, HGFAC, HRG, ITGB1, ITGB3, KLKB1, KNG1, MCAM, MYH9, PLEK, PLG, PROC, PROS1, PROZ, PTPRJ, SERPINC1, SERPIND1, SERPINF2, TUBB1	28	4,67E-18
**regulation of body fluid levels**	BP	ADAMTS13, APOH, CPB2, F10, F11, F12, F13A1, F2, FAP, HGFAC, HRG, ITGB3, KLKB1, KNG1, MYH9, PLEK, PLG, PROC, PROS1, PROZ, PTPRJ, SERPINC1, SERPIND1, SERPINF2, TUBB1, UMOD	26	1,53E-17

**complement activation**	BP	C1QA, C1QB, C1RL, C3, C5, C6, C8A, C8B, C8G, CFH, CFI, CLU, COLEC10, FCN3, MASP1	15	2,40E-17
**regulation of fibrinolysis**	BP	APOH, CPB2, F11, F12, F2, FAP, HRG, KLKB1, PLG, SERPINF2	10	6,56E-16
**negative regulation of blood coagulation**	BP	APOH, CPB2, F11, F12, F2, FAP, HRG, KLKB1, KNG1, PLG, PROC, PROS1, SERPINF2	13	6,81E-16
**negative regulation of hemostasis**	BP	APOH, CPB2, F11, F12, F2, FAP, HRG, KLKB1, KNG1, PLG, PROC, PROS1, SERPINF2	13	7,34E-16
**fibrinolysis**	BP	APOH, CPB2, F11, F12, F2, FAP, HRG, KLKB1, PLG, PROS1, SERPINF2	11	7,34E-16
**regulation of blood coagulation**	BP	APOH, CPB2, F11, F12, F2, FAP, HRG, KLKB1, KNG1, PLG, PROC, PROS1, SERPINC1, SERPINF2	14	1,07E-15
**negative regulation of coagulation**	BP	APOH, CPB2, F11, F12, F2, FAP, HRG, KLKB1, KNG1, PLG, PROC, PROS1, SERPINF2	13	1,39E-15
**regulation of hemostasis**	BP	APOH, CPB2, F11, F12, F2, FAP, HRG, KLKB1, KNG1, PLG, PROC, PROS1, SERPINC1, SERPINF2	14	1,40E-15
**regulation of coagulation**	BP	APOH, CPB2, F11, F12, F2, FAP, HRG, KLKB1, KNG1, PLG, PROC, PROS1, SERPINC1, SERPINF2	14	3,61E-15
**complement activation, classical pathway**	BP	C1QA, C1QB, C1RL, C3, C5, C6, C8A, C8B, C8G, CFI, CLU	11	1,49E-13
**negative regulation of wound healing**	BP	APOH, CPB2, F11, F12, F2, FAP, HRG, KLKB1, KNG1,	13	1,84E-13

		PLG, PROC, PROS1, SERPINF2		
**regulation of wound healing**	BP	APOH, CPB2, F11, F12, F2, FAP, HRG, ITGB1, KLKB1, KNG1, PLG, PROC, PROS1, SERPINC1, SERPINF2	15	9,29E-13
**protein processing**	BP	ACE, ADAMTS13, APOH, BCHE, C1RL, CLEC3B, CPB2, DPP4, ENPEP, F11, F12, GSN, HGFAC, KLKB1, MASP1, MYH9, PLG, SERPINF2	18	1,29E-12
**humoral immune response mediated by circulating immunoglobulin**	BP	C1QA, C1QB, C1RL, C3, C5, C6, C8A, C8B, C8G, CFI, CLU	11	3,81E-12
**negative regulation of response to wounding**	BP	APOH, CPB2, F11, F12, F2, FAP, HRG, KLKB1, KNG1, PLG, PROC, PROS1, SERPINF2	13	4,84E-12
**regulation of response to wounding**	BP	APOH, CPB2, F11, F12, F2, FAP, HRG, ITGB1, KLKB1, KNG1, PLG, PROC, PROS1, SERPINC1, SERPINF2	15	3,46E-11
**humoral immune response**	BP	C1QA, C1QB, C1RL, C3, C5, C6, C8A, C8B, C8G, CFH, CFI, CLU, COLEC10, F2, FCN3, HRG, KNG1, MASP1	18	5,63E-11
**zymogen activation**	BP	APOH, C1RL, CLEC3B, CPB2, F11, F12, HGFAC, KLKB1, MASP1, SERPINF2	10	1,94E-10

**negative regulation of peptidase activity**	BP	ECM1, FETUB, ITIH1, ITIH2, ITIH4, KNG1, PI16, SERPINA4, SERPINA5, SERPINA6, SERPINA7, SERPINC1, SERPIND1, SERPINF2	14	1,69E-09
**negative regulation of hydrolase activity**	BP	APOC1, ECM1, FETUB, ITIH1, ITIH2, ITIH4, KNG1, PI16, SERPINA4, SERPINA5, SERPINA6, SERPINA7, SERPINC1, SERPIND1, SERPINF2	15	3,45E-09
**negative regulation of proteolysis**	BP	CPB2, ECM1, F2, FETUB, ITIH1, ITIH2, ITIH4, KNG1, PI16, SERPINA4, SERPINA5, SERPINA6, SERPINA7, SERPINC1, SERPIND1, SERPINF2	16	4,25E-09
**negative regulation of fibrinolysis**	BP	APOH, CPB2, F2, HRG, PLG, SERPINF2	6	6,28E-09
**positive regulation of blood coagulation**	BP	APOH, CPB2, F12, F2, HRG, PLG, SERPINF2	7	1,59E-08
**plasminogen activation**	BP	APOH, CLEC3B, CPB2, F11, F12, KLKB1, SERPINF2	7	1,59E-08
**positive regulation of hemostasis**	BP	APOH, CPB2, F12, F2, HRG, PLG, SERPINF2	7	1,59E-08
**positive regulation of coagulation**	BP	APOH, CPB2, F12, F2, HRG, PLG, SERPINF2	7	4,46E-08
**complement activation, alternative pathway**	BP	C3, C5, C8A, C8B, C8G, CFH	6	5,59E-08

**negative regulation of catalytic activity**	BP	ADIPOQ, APOC1, ECM1, ENG, FETUB, ITIH1, ITIH2, ITIH4, KNG1, PI16, PTPRJ, SERPINA4, SERPINA5, SERPINA6, SERPINA7, SERPINC1, SERPIND1, SERPINF2	18	6,15E-08
**lipid transport**	BP	ACE, ADIPOQ, APOA4, APOB, APOC1, APOC4, APOD, APOF, APOH, APOM, CETP, CLU, ITGB3, LCAT, PON1, RBP4, SERPINA5	17	1,83E-07
**regulation of peptidase activity**	BP	ECM1, FETUB, GSN, ITIH1, ITIH2, ITIH4, KNG1, PI16, SERPINA4, SERPINA5, SERPINA6, SERPINA7, SERPINC1, SERPIND1, SERPINF2	15	2,00E-07
**immunoglobulin mediated immune response**	BP	C1QA, C1QB, C1RL, C3, C5, C6, C8A, C8B, C8G, CFI, CLU, IGHV3-7	12	3,63E-07
**leukocyte mediated immunity**	BP	ACE, C1QA, C1QB, C1RL, C3, C5, C6, C8A, C8B, C8G, CFI, CLU, DBH, F2, IGHV3-7, KIT, LAMP1	17	3,84E-07
**positive regulation of wound healing**	BP	APOH, CPB2, F12, F2, HRG, ITGB1, PLG, SERPINF2	8	3,84E-07
**B cell mediated immunity**	BP	C1QA, C1QB, C1RL, C3, C5, C6, C8A, C8B, C8G, CFI, CLU, IGHV3-7	12	3,93E-07

**negative regulation of response to external stimulus**	BP	ADIPOQ, APOH, C5, CPB2, DPP4, F11, F12, F2, FAP, HRG, KLKB1, KNG1, PLG, PROC, PROS1, SERPINF2	16	5,60E-07
**positive regulation of response to wounding**	BP	APOH, CPB2, F12, F2, HRG, ITGB1, PLG, SERPINF2	8	1,85E-06
**protein-lipid complex remodeling**	BP	APOA4, APOB, APOC1, APOM, CETP, LCAT	6	2,30E-06
**plasma lipoprotein particle remodeling**	BP	APOA4, APOB, APOC1, APOM, CETP, LCAT	6	2,30E-06
**high-density lipoprotein particle remodeling**	BP	APOA4, APOC1, APOM, CETP, LCAT	5	2,32E-06
**cholesterol transport**	BP	ADIPOQ, APOA4, APOB, APOC1, APOM, CETP, CLU, LCAT, PON1	9	3,13E-06
**protein-containing complex remodeling**	BP	APOA4, APOB, APOC1, APOM, CETP, LCAT	6	3,13E-06
**regulation of plasma lipoprotein particle levels**	BP	ADIPOQ, APOA4, APOB, APOC1, APOM, CETP, GPLD1, LCAT	8	4,04E-06
**reverse cholesterol transport**	BP	APOA4, APOM, CETP, CLU, LCAT	5	5,15E-06
**protein activation cascade**	BP	APOH, F12, F13A1, ITGB3, KLKB1	5	5,15E-06
**sterol transport**	BP	ADIPOQ, APOA4, APOB, APOC1, APOM, CETP, CLU, LCAT, PON1	9	5,26E-06
**regulation of protein processing**	BP	CLEC3B, CPB2, F12, GSN, KLKB1, MYH9, SERPINF2	7	7,48E-06
**phagocytosis**	BP	ADIPOQ, AHSG, BIN2, C3, COLEC10, FCN3, GSN, ITGB1, ITGB3, MYH9, PTPRJ	11	1,06E-05
**regulation of protein maturation**	BP	CLEC3B, CPB2, F12, GSN, KLKB1, MYH9, SERPINF2	7	1,31E-05
**cholesterol efflux**	BP	ADIPOQ, APOA4, APOB, APOC1, APOM, CETP, PON1	7	1,41E-05
**positive regulation of protein processing**	BP	CLEC3B, F12, GSN, KLKB1, MYH9	5	1,53E-05

**plasma lipoprotein particle assembly**	BP	APOA4, APOB, APOC1, APOM, LCAT	5	2,23E-05
**positive regulation of protein maturation**	BP	CLEC3B, F12, GSN, KLKB1, MYH9	5	2,23E-05
**plasma lipoprotein particle organization**	BP	APOA4, APOB, APOC1, APOM, CETP, LCAT	6	2,25E-05
**cell killing**	BP	C3, C5, C6, C8A, C8B, C8G, CFH, F2, HRG, KNG1, LAMP1	11	2,51E-05
**lymphocyte mediated immunity**	BP	C1QA, C1QB, C1RL, C3, C5, C6, C8A, C8B, C8G, CFI, CLU, IGHV3-7, LAMP1	13	2,57E-05
**protein-lipid complex organization**	BP	APOA4, APOB, APOC1, APOM, CETP, LCAT	6	3,05E-05
**killing of cells of another organism**	BP	C5, C6, C8A, C8B, C8G, F2, HRG, KNG1	8	3,31E-05
**disruption of cell in another organism**	BP	C5, C6, C8A, C8B, C8G, F2, HRG, KNG1	8	3,31E-05
**protein-lipid complex assembly**	BP	APOA4, APOB, APOC1, APOM, LCAT	5	3,45E-05
**disruption of anatomical structure in another organism**	BP	C5, C6, C8A, C8B, C8G, F2, HRG, KNG1	8	3,84E-05
**leukocyte migration**	BP	APOD, C5, DBH, DPP4, ECM1, ITGB1, ITGB3, KIT, MYH9, PLG, PTPRJ, SELL, UMOD	13	3,89E-05
**synapse pruning**	BP	C1QA, C1QB, C3, ITGB1	4	5,74E-05
**organic hydroxy compound transport**	BP	ADIPOQ, APOA4, APOB, APOC1, APOM, CETP, CLU, ITGB3, LCAT, PON1, RBP4	11	5,85E-05
**blood coagulation, fibrin clot formation**	BP	APOH, F12, F13A1, ITGB3	4	7,39E-05
**regulation of lipid localization**	BP	ADIPOQ, APOA4, APOB, APOC1, APOC4, C3, CETP, ITGB3, PON1	9	7,42E-05
**platelet activation**	BP	ADAMTS13, F2, HRG, ITGB3, MYH9, PLEK, PTPRJ, TUBB1	8	7,74E-05

**lipoprotein metabolic process**	BP	APOA4, APOB, APOC1, APOD, APOM, ITGB3, LCAT	7	9,43E-05
**regulation of plasminogen activation**	BP	CLEC3B, CPB2, F12, SERPINF2	4	0,000116369
**triglyceride metabolic process**	BP	APOA4, APOB, APOC1, APOH, C3, CETP, GPLD1	7	0,000134488
**biological process involved in symbiotic interaction**	BP	DPP4, F2, FCN3, GSN, HRG, ITGB1, ITGB3, LAMP1, NCAM1, PGLYRP2, PLG	11	0,000135842
**negative regulation of endopeptidase activity**	BP	FETUB, SERPINA4, SERPINA5, SERPINA6, SERPINA7, SERPIND1, SERPINF2	7	0,000139235
**acute inflammatory response**	BP	AHSG, C3, F12, F2, ITIH4, KLKB1, SERPINF2	7	0,000146032
**adaptive immune response based on somatic recombination of immune receptors built from immunoglobulin superfamily domains**	BP	C1QA, C1QB, C1RL, C3, C5, C6, C8A, C8B, C8G, CFI, CLU, IGHV3-7	12	0,000178398
**chemotaxis**	BP	BIN2, C5, CDH13, COLEC10, DPP4, ENG, GPNMB, HRG, ITGB3, KIT, MEGF8, PTPRJ, SERPIND1	13	0,000178843
**taxis**	BP	BIN2, C5, CDH13, COLEC10, DPP4, ENG, GPNMB, HRG, ITGB3, KIT, MEGF8, PTPRJ, SERPIND1	13	0,000184826
**phosphatidylcholine metabolic process**	BP	APOA4, APOC1, CETP, GPLD1, LCAT, PON1	6	0,000210505
**regulation of collagen metabolic process**	BP	ENG, F2, FAP, ITGB1, SERPINF2	5	0,000214547
**endothelial cell migration**	BP	APOH, CDH13, DPP4, FAP, GPLD1, HRG, ITGB1, ITGB3, MYH9, PLG	10	0,000286496

**alcohol metabolic process**	BP	APOA4, APOB, APOC1, APOF, CETP, DBH, LCAT, MINPP1, PLEK, PON1, RBP4	11	0,000375104
**regulation of endocytosis**	BP	ADIPOQ, AHSG, APOC1, C3, CDH13, CLU, COLEC10, FCN3, ITGB3, PTPRJ	10	0,000500791
**acylglycerol metabolic process**	BP	APOA4, APOB, APOC1, APOH, C3, CETP, GPLD1	7	0,00050176
**neutral lipid metabolic process**	BP	APOA4, APOB, APOC1, APOH, C3, CETP, GPLD1	7	0,000520293
**plasma lipoprotein particle clearance**	BP	ADIPOQ, APOB, APOC1, APOM, GPLD1	5	0,000542212
**tissue remodeling**	BP	ACE, DBH, GPNMB, HRG, ITGB3, PLG, SPP2, UMOD	8	0,000573908
**cell junction disassembly**	BP	C1QA, C1QB, C3, ITGB1	4	0,000602465
**cholesterol metabolic process**	BP	APOA4, APOB, APOC1, APOF, CETP, LCAT, PON1	7	0,000857588
**complement activation, lectin pathway**	BP	COLEC10, FCN3, MASP1	3	0,000862241
**biological process involved in interaction with host**	BP	DPP4, FCN3, GSN, ITGB1, ITGB3, LAMP1, NCAM1, PGLYRP2	8	0,000879381
**regulation of phagocytosis**	BP	ADIPOQ, AHSG, C3, COLEC10, FCN3, PTPRJ	6	0,000910195
**steroid metabolic process**	BP	APOA4, APOB, APOC1, APOF, CETP, GC, KIT, LCAT, PON1, SERPINA6	10	0,000918327
**extracellular matrix organization**	BP	ADAMTS13, CPB2, DPP4, ENG, FAP, ITGB1, ITGB3, LUM, PLG, SERPINF2	10	0,001029936
**extracellular structure organization**	BP	ADAMTS13, CPB2, DPP4, ENG, FAP, ITGB1, ITGB3, LUM, PLG, SERPINF2	10	0,001044875
**external encapsulating structure organization**	BP	ADAMTS13, CPB2, DPP4, ENG, FAP, ITGB1, ITGB3, LUM, PLG, SERPINF2	10	0,00104793
**triglyceride-rich lipoprotein particle remodeling**	BP	APOA4, CETP, LCAT	3	0,00104793

**very-low-density lipoprotein particle remodeling**	BP	APOA4, CETP, LCAT	3	0,00104793
**regulation of macrophage derived foam cell differentiation**	BP	ADIPOQ, APOB, CETP, ITGB3	4	0,00104793
**sterol metabolic process**	BP	APOA4, APOB, APOC1, APOF, CETP, LCAT, PON1	7	0,001184937
**symbiont entry into host cell**	BP	DPP4, FCN3, GSN, ITGB1, ITGB3, LAMP1, NCAM1	7	0,001184937
**secondary alcohol metabolic process**	BP	APOA4, APOB, APOC1, APOF, CETP, LCAT, PON1	7	0,001184937
**peptide hormone processing**	BP	ACE, BCHE, DPP4, ENPEP	4	0,001274087
**negative regulation of platelet-derived growth factor receptor signaling pathway**	BP	ADIPOQ, APOD, PTPRJ	3	0,001274087
**glycosaminoglycan metabolic process**	BP	B3GNT2, B4GAT1, ITIH1, ITIH2, ITIH4, PGLYRP2	6	0,001274087
**collagen metabolic process**	BP	ENG, F2, FAP, ITGB1, PCOLCE, SERPINF2	6	0,001274087
**symbiont entry into host**	BP	DPP4, FCN3, GSN, ITGB1, ITGB3, LAMP1, NCAM1	7	0,001375164
**positive regulation of lipid localization**	BP	ADIPOQ, APOB, APOC4, C3, CETP, PON1	6	0,001382105
**negative regulation of macrophage derived foam cell differentiation**	BP	ADIPOQ, CETP, ITGB3	3	0,001541699
**regulation of lipoprotein lipase activity**	BP	APOA4, APOC1, APOH	3	0,001541699
**macrophage derived foam cell differentiation**	BP	ADIPOQ, APOB, CETP, ITGB3	4	0,001650291
**positive regulation of phagocytosis**	BP	AHSG, C3, COLEC10, FCN3, PTPRJ	5	0,001655908
**foam cell differentiation**	BP	ADIPOQ, APOB, CETP, ITGB3	4	0,001782928
**signaling receptor ligand precursor processing**	BP	ACE, BCHE, DPP4, ENPEP	4	0,001782928

**regulation of smooth muscle cell proliferation**	BP	ADIPOQ, APOD, CDH13, DBH, IGFBP3, ITGB3, SERPINF2	7	0,001858138
**aminoglycan metabolic process**	BP	B3GNT2, B4GAT1, ITIH1, ITIH2, ITIH4, PGLYRP2	6	0,001971539
**regulation of sterol transport**	BP	ADIPOQ, APOA4, APOC1, CETP, PON1	5	0,002029514
**regulation of cholesterol transport**	BP	ADIPOQ, APOA4, APOC1, CETP, PON1	5	0,002029514
**smooth muscle cell proliferation**	BP	ADIPOQ, APOD, CDH13, DBH, IGFBP3, ITGB3, SERPINF2	7	0,002070571
**glycerolipid metabolic process**	BP	APOA4, APOB, APOC1, APOH, C3, CETP, GPLD1, LCAT, PLEK, PON1	10	0,002326457
**regulation of endopeptidase activity**	BP	FETUB, GSN, SERPINA4, SERPINA5, SERPINA6, SERPINA7, SERPIND1, SERPINF2	8	0,002604577
**muscle cell proliferation**	BP	ADIPOQ, APOD, CDH13, DBH, IGFBP3, ITGB3, RBP4, SERPINF2	8	0,002869266
**renal system process**	BP	ADIPOQ, GSN, MCAM, PON3, SERPINF2, UMOD	6	0,002869266
**phagocytosis, engulfment**	BP	BIN2, C3, GSN, MYH9	4	0,003692887
**acute-phase response**	BP	AHSG, F2, ITIH4, SERPINF2	4	0,003692887
**peptide catabolic process**	BP	ACE, ADAMTS13, ENPEP	3	0,00411332
**mucopolysaccharide metabolic process**	BP	B3GNT2, B4GAT1, ITIH1, ITIH2, ITIH4	5	0,004152156
**regulation of lipid metabolic process**	BP	ADIPOQ, APOA4, APOB, APOC1, APOD, C3, F2, GPLD1, KIT	9	0,004549668
**collagen biosynthetic process**	BP	ENG, F2, PCOLCE, SERPINF2	4	0,004888424
**regulation of platelet activation**	BP	F2, HRG, PLEK, PTPRJ	4	0,005614253
**regulation of lipid storage**	BP	APOB, APOC4, C3, ITGB3	4	0,005980926

**regulation of complement activation**	BP	C3, CFH, MASP1	3	0,006022946
**positive regulation of cell-matrix adhesion**	BP	CDH13, HRG, ITGB3, PTPRJ	4	0,006315866
**regulation of lipid transport**	BP	ADIPOQ, APOA4, APOC1, CETP, ITGB3, PON1	6	0,006498407
**plasma membrane invagination**	BP	BIN2, C3, GSN, MYH9	4	0,006606292
**regulation of systemic arterial blood pressure by renin-angiotensin**	BP	ACE, ENPEP, SERPINF2	3	0,006606292
**regulation of platelet-derived growth factor receptor signaling pathway**	BP	ADIPOQ, APOD, PTPRJ	3	0,006606292
**positive regulation of endocytosis**	BP	AHSG, C3, CLU, COLEC10, FCN3, PTPRJ	6	0,006986141
**cellular component disassembly**	BP	C1QA, C1QB, C3, DPP4, ENG, FAP, GSN, ITGB1, PLEK, PLG	10	0,007002618
**platelet formation**	BP	MYH9, PTPRJ, TUBB1	3	0,007285194
**platelet-derived growth factor receptor signaling pathway**	BP	ADIPOQ, APOD, ITGB3, PTPRJ	4	0,007285194
**cholesterol homeostasis**	BP	APOA4, APOB, APOM, CETP, LCAT	5	0,007538265
**sterol homeostasis**	BP	APOA4, APOB, APOM, CETP, LCAT	5	0,007815379
**negative regulation of cell migration**	BP	ADIPOQ, APOD, APOH, C5, DPP4, ENG, HRG, IGFBP3, PTPRJ	9	0,008788311
**positive regulation of lipid storage**	BP	APOB, APOC4, C3	3	0,008788311
**positive regulation of collagen biosynthetic process**	BP	ENG, F2, SERPINF2	3	0,008788311
**platelet morphogenesis**	BP	MYH9, PTPRJ, TUBB1	3	0,008788311
**integrin-mediated signaling pathway**	BP	ADAMTS13, ITGB1, ITGB3, MYH9, PLEK	5	0,008933708

**axon guidance**	BP	B3GNT2, B4GAT1, CHL1, CNTN1, MEGF8, NCAM1, PTPRJ	7	0,009212752
**membrane invagination**	BP	BIN2, C3, GSN, MYH9	4	0,009322439
**neuron projection guidance**	BP	B3GNT2, B4GAT1, CHL1, CNTN1, MEGF8, NCAM1, PTPRJ	7	0,009322439
**positive regulation of collagen metabolic process**	BP	ENG, F2, SERPINF2	3	0,009419914
**triglyceride catabolic process**	BP	APOA4, APOB, GPLD1	3	0,009419914
**extracellular matrix disassembly**	BP	DPP4, ENG, FAP, PLG	4	0,009658739
**cell-matrix adhesion**	BP	ADAMTS13, APOD, CDH13, HRG, ITGB1, ITGB3, PTPRJ	7	0,01002745
**peptide metabolic process**	BP	ACE, ADAMTS13, ENPEP, PGLYRP2	4	0,010040736
**glycerolipid catabolic process**	BP	APOA4, APOB, APOC1, GPLD1	4	0,010040736
**epithelial cell proliferation**	BP	APOH, CDH13, CPB2, DBH, ECM1, ENG, FAP, IGFBP3, ITGB3, KIT	10	0,010495728
**negative regulation of cell motility**	BP	ADIPOQ, APOD, APOH, C5, DPP4, ENG, HRG, IGFBP3, PTPRJ	9	0,01053319
**regulation of endothelial cell proliferation**	BP	APOH, CDH13, DBH, ECM1, ENG, ITGB3	6	0,011258728
**lipid homeostasis**	BP	APOA4, APOB, APOC4, APOM, CETP, LCAT	6	0,011518686
**regulation of immune effector process**	BP	C3, CFH, COLEC10, FCN3, KIT, LAMP1, MASP1, PGLYRP2, RBP4	9	0,01274839
**lamellipodium assembly**	BP	CDH13, HRG, ITGB1, KIT	4	0,013347948
**positive chemotaxis**	BP	CDH13, COLEC10, GPNMB, PTPRJ	4	0,013347948
**regulation of cell-matrix adhesion**	BP	APOD, CDH13, HRG, ITGB3, PTPRJ	5	0,013724167

**negative regulation of locomotion**	BP	ADIPOQ, APOD, APOH, C5, DPP4, ENG, HRG, IGFBP3, PTPRJ	9	0,013777051
**heart trabecula morphogenesis**	BP	ADGRG6, ENG, RBP4	3	0,014072617
**regulation of extracellular matrix organization**	BP	CPB2, DPP4, FAP, ITGB3	4	0,014441662
**regulation of blood pressure**	BP	ACE, ADIPOQ, ENG, ENPEP, SERPINF2, UMOD	6	0,014523253
**regulation of receptor-mediated endocytosis**	BP	ADIPOQ, APOC1, C3, CLU, ITGB3	5	0,014815773
**hyaluronan metabolic process**	BP	ITIH1, ITIH2, ITIH4	3	0,015010831
**positive regulation of phosphatidylinositol 3-kinase/protein kinase B signal transduction**	BP	ENG, F2, ITGB1, KIT, PROS1, PTPRJ	6	0,015062279
**glucose homeostasis**	BP	ACE, ADIPOQ, CPB2, DBH, GPLD1, PTPRJ, RBP4	7	0,015639027
**carbohydrate homeostasis**	BP	ACE, ADIPOQ, CPB2, DBH, GPLD1, PTPRJ, RBP4	7	0,01589113
**regulation of lipase activity**	BP	APOA4, APOC1, APOH	3	0,015976006
**platelet aggregation**	BP	ITGB3, MYH9, PLEK, TUBB1	4	0,015995753
**regulation of small molecule metabolic process**	BP	ADIPOQ, APOA4, APOB, APOC1, GPLD1, IGFBP3, KIT, PLEK	8	0,016811184
**positive regulation of sterol transport**	BP	ADIPOQ, CETP, PON1	3	0,016967763
**positive regulation of cholesterol transport**	BP	ADIPOQ, CETP, PON1	3	0,016967763
**negative regulation of receptor-mediated endocytosis**	BP	ADIPOQ, APOC1, ITGB3	3	0,018282189
**receptor-mediated endocytosis**	BP	ADIPOQ, APOC1, C3, CLU, DPP4, ITGB1, ITGB3	7	0,018462705
**endothelial cell proliferation**	BP	APOH, CDH13, DBH, ECM1, ENG, ITGB3	6	0,018462705
**regulation of systemic arterial blood pressure by hormone**	BP	ACE, ENPEP, SERPINF2	3	0,019032327

**renal absorption**	BP	ADIPOQ, GSN, UMOD	3	0,019032327
**regulation of angiotensin levels in blood**	BP	ACE, ENPEP	2	0,019032327
**positive regulation of phosphatase activity**	BP	GPLD1, PLEK	2	0,019032327
**peripheral nervous system axon regeneration**	BP	APOA4, APOD	2	0,019032327
**renal protein absorption**	BP	ADIPOQ, GSN	2	0,019032327
**regulation of angiogenesis**	BP	APOH, C3, ECM1, ENG, GPNMB, HRG, ITGB1, ITGB3	8	0,019508259
**cell-substrate adhesion**	BP	ADAMTS13, APOD, CDH13, HRG, ITGB1, ITGB3, PLG, PTPRJ	8	0,019747094
**detection of biotic stimulus**	BP	FAP, PGLYRP2, PTPRJ	3	0,019892595
**neutral lipid catabolic process**	BP	APOA4, APOB, GPLD1	3	0,019892595
**acylglycerol catabolic process**	BP	APOA4, APOB, GPLD1	3	0,019892595
**response to xenobiotic stimulus**	BP	ACE, ADIPOQ, BCHE, CPB2, ENG, GPLD1, ITGB3, PON3, UMOD	9	0,02061089
**regulation of collagen biosynthetic process**	BP	ENG, F2, SERPINF2	3	0,021086006
**positive regulation of G protein-coupled receptor signaling pathway**	BP	C3, F2, ITGB3	3	0,021086006
**regulation of vasculature development**	BP	APOH, C3, ECM1, ENG, GPNMB, HRG, ITGB1, ITGB3	8	0,021685228
**positive regulation of dephosphorylation**	BP	GPLD1, PLEK	2	0,021685228
**cytolysis by host of symbiont cells**	BP	F2, HRG	2	0,021685228
**complement-dependent cytotoxicity**	BP	C3, CFH	2	0,021685228
**regulation of opsonization**	BP	COLEC10, FCN3	2	0,021685228
**axonogenesis**	BP	B3GNT2, B4GAT1, CHL1, CNTN1, ITGB1, MEGF8, NCAM1, PTPRJ, RAB10	9	0,0223107

**regulation of mononuclear cell migration**	BP	APOD, C5, ECM1, ITGB3, PTPRJ	5	0,022896831
**regulation of chemotaxis**	BP	C5, CDH13, DPP4, HRG, MEGF8, PTPRJ	6	0,023128285
**cell adhesion mediated by integrin**	BP	DPP4, HRG, ITGB1, ITGB3	4	0,023464228
**glycerophospholipid metabolic process**	BP	APOA4, APOC1, CETP, GPLD1, LCAT, PLEK, PON1	7	0,023751554
**epiboly**	BP	ITGB1, ITGB3, MEGF8	3	0,024622682
**very-low-density lipoprotein particle assembly**	BP	APOB, APOC1	2	0,024845921
**thyroid hormone transport**	BP	SERPINA7, TUBB1	2	0,024845921
**lamellipodium organization**	BP	CDH13, HRG, ITGB1, KIT	4	0,024845921
**regulation of cell-substrate adhesion**	BP	APOD, CDH13, HRG, ITGB3, PLG, PTPRJ	6	0,026229613
**regulation of smooth muscle cell migration**	BP	ACE, ADIPOQ, IGFBP3, ITGB3	4	0,026597642
**regulation of tube diameter**	BP	ACE, DBH, ITGB1, KNG1, SERPINF2	5	0,026597642
**blood vessel diameter maintenance**	BP	ACE, DBH, ITGB1, KNG1, SERPINF2	5	0,026597642
**regulation of systemic arterial blood pressure mediated by a chemical signal**	BP	ACE, ENPEP, SERPINF2	3	0,02665914
**apoptotic cell clearance**	BP	C3, FCN3, ITGB3	3	0,02665914
**acylglycerol homeostasis**	BP	APOA4, APOC4, CETP	3	0,02665914
**cellular response to lipoprotein particle stimulus**	BP	APOB, CDH13, ITGB1	3	0,02665914
**regulation of tube size**	BP	ACE, DBH, ITGB1, KNG1, SERPINF2	5	0,026733608
**cell recognition**	BP	COLEC10, CRTAC1, FCN3, FETUB, MEGF8	5	0,027283322
**positive regulation of platelet activation**	BP	PLEK, PTPRJ	2	0,027283322
**regulation of triglyceride catabolic process**	BP	APOA4, GPLD1	2	0,027283322

**high-density lipoprotein particle assembly**	BP	APOM, LCAT	2	0,027283322
**extracellular matrix constituent secretion**	BP	CPB2, ENG	2	0,027283322
**positive regulation of vascular associated smooth muscle cell differentiation**	BP	ENG, KIT	2	0,027283322
**regulation of triglyceride metabolic process**	BP	APOA4, C3, GPLD1	3	0,027396918
**cell-substrate junction assembly**	BP	APOD, HRG, ITGB3, PTPRJ	4	0,027930664
**lipid storage**	BP	APOB, APOC4, C3, ITGB3	4	0,028800719
**mononuclear cell migration**	BP	APOD, C5, ECM1, ITGB3, PLG, PTPRJ	6	0,028800719
**regulation of systemic arterial blood pressure**	BP	ACE, ENG, ENPEP, SERPINF2	4	0,029700857
**regulation of acute inflammatory response**	BP	C3, F12, KLKB1	3	0,030019323
**regulation of humoral immune response**	BP	C3, CFH, MASP1	3	0,030019323
**trabecula morphogenesis**	BP	ADGRG6, ENG, RBP4	3	0,030019323
**negative regulation of cell adhesion**	BP	ADIPOQ, APOD, CDH13, GPNMB, HRG, KNG1, PLG	7	0,030111945
**regulation of peptidyl-tyrosine phosphorylation**	BP	ADIPOQ, HRG, ITGB3, KIT, PTPRJ	5	0,030111945
**positive regulation of systemic arterial blood pressure**	BP	ACE, ENG	2	0,030111945
**phospholipid efflux**	BP	APOA4, APOC1	2	0,030111945
**regulation of leukocyte migration**	BP	APOD, C5, DPP4, ECM1, ITGB3, PTPRJ	6	0,030749281
**smooth muscle cell migration**	BP	ACE, ADIPOQ, IGFBP3, ITGB3	4	0,030749281
**regulation of cell adhesion mediated by integrin**	BP	DPP4, HRG, ITGB3	3	0,030915166
**homotypic cell-cell adhesion**	BP	ITGB3, MYH9, PLEK, TUBB1	4	0,031606272
**cell-cell adhesion mediated by cadherin**	BP	CDH13, PLG, SERPINF2	3	0,032342493

**regulation of T cell migration**	BP	APOD, ECM1, ITGB3	3	0,032342493
**cellular response to peptide hormone stimulus**	BP	ACE, ADIPOQ, AHSG, GPLD1, ITGB3, PTPRJ, RAB10	7	0,032684622
**cell-substrate junction organization**	BP	APOD, HRG, ITGB3, PTPRJ	4	0,032820991
**establishment of T cell polarity**	BP	GSN, MYH9	2	0,032820991
**complement receptor mediated signaling pathway**	BP	C3, GPLD1	2	0,032820991
**positive regulation of fibroblast migration**	BP	ITGB1, ITGB3	2	0,032820991
**neuron remodeling**	BP	C1QA, C3	2	0,032820991
**heart trabecula formation**	BP	ADGRG6, RBP4	2	0,032820991
**viral life cycle**	BP	DPP4, FCN3, GSN, ITGB1, ITGB3, LAMP1, NCAM1	7	0,032878331
**visual learning**	BP	DBH, ITGB1, KIT	3	0,033000142
**positive regulation of proteolysis**	BP	CLEC3B, CLU, F12, GPLD1, GSN, KLKB1, MYH9	7	0,033738333
**blood vessel remodeling**	BP	ACE, DBH, HRG	3	0,034459202
**negative regulation of chemotaxis**	BP	C5, DPP4, HRG	3	0,034459202
**cell chemotaxis**	BP	BIN2, C5, DPP4, ENG, HRG, KIT, PTPRJ	7	0,035057099
**establishment of lymphocyte polarity**	BP	GSN, MYH9	2	0,035934028
**renal sodium ion transport**	BP	PON3, UMOD	2	0,035934028
**keratan sulfate biosynthetic process**	BP	B3GNT2, B4GAT1	2	0,035934028
**low-density lipoprotein particle remodeling**	BP	APOB, CETP	2	0,035934028
**plasma membrane organization**	BP	BIN2, CLU, GSN, MYH9, SERPINA5	5	0,035934028
**regulation of cholesterol efflux**	BP	ADIPOQ, CETP, PON1	3	0,037318875
**opsonization**	BP	COLEC10, FCN3	2	0,039655621

**regulation of lipoprotein metabolic process**	BP	APOD, ITGB3	2	0,039655621
**actin filament severing**	BP	GSN, MYH9	2	0,039655621
**lactone metabolic process**	BP	PON1, PON3	2	0,039655621
**negative regulation of leukocyte migration**	BP	APOD, C5, DPP4	3	0,042636726
**blood vessel endothelial cell migration**	BP	GPLD1, HRG, ITGB1, MYH9, PLG	5	0,042991479
**muscle cell migration**	BP	ACE, ADIPOQ, IGFBP3, ITGB3	4	0,042991479
**regulation of systemic arterial blood pressure by circulatory renin-angiotensin**	BP	ACE, ENPEP	2	0,042991479
**regulation of extracellular matrix disassembly**	BP	DPP4, FAP	2	0,042991479
**cell-cell adhesion mediated by integrin**	BP	DPP4, ITGB1	2	0,042991479
**high-density lipoprotein particle clearance**	BP	APOM, GPLD1	2	0,042991479
**keratan sulfate metabolic process**	BP	B3GNT2, B4GAT1	2	0,042991479
**regulation of plasma membrane organization**	BP	GSN, MYH9	2	0,042991479
**myeloid leukocyte mediated immunity**	BP	ACE, C3, F2, KIT	4	0,045185995
**visual behavior**	BP	DBH, ITGB1, KIT	3	0,045272165
**positive regulation of transforming growth factor beta production**	BP	LUM, SERPINF2	2	0,047345609
**positive regulation of peptidyl-tyrosine phosphorylation**	BP	ADIPOQ, ITGB3, KIT, PTPRJ	4	0,047467341
**glial cell differentiation**	BP	ADGRG6, C1QA, CLU, CNTN1, F2, PTPRJ	6	0,047593988
**positive regulation of angiogenesis**	BP	C3, ECM1, ENG, ITGB1, ITGB3	5	0,048375671

**blood microparticle**	CC	A1BG, AFM, AHSG, APOA4, BCHE, C1QB, C3, C8A, C8G, CFH, CLU, ENG, F13A1, F2, FCN3, GC, GSN, HRG, IGHV3-7, ITIH1, ITIH2, ITIH4, KNG1, PLG, PON1, PROS1, SERPINC1, SERPINF2	28	8,75E-33
**collagen-containing extracellular matrix**	CC	A1BG, ADIPOQ, AHSG, APOA4, APOH, AZGP1, C1QA, C1QB, CDH13, CLEC3B, CLU, ECM1, F12, F13A1, F2, FCN3, HRG, ITIH1, ITIH2, ITIH4, KNG1, LUM, NCAM1, PCOLCE, PLG, SERPINA5, SERPINC1, SERPINF2, SPP2	29	1,07E-20
**plasma lipoprotein particle**	CC	APOA4, APOB, APOC1, APOC4, APOF, APOH, APOM, CETP, CLU, LCAT, PCYOX1, PON1	12	8,99E-17
**lipoprotein particle**	CC	APOA4, APOB, APOC1, APOC4, APOF, APOH, APOM, CETP, CLU, LCAT, PCYOX1, PON1	12	8,99E-17
**protein-lipid complex**	CC	APOA4, APOB, APOC1, APOC4, APOF, APOH, APOM, CETP, CLU, LCAT, PCYOX1, PON1	12	2,14E-16

**secretory granule lumen**	CC	A1BG, AHSG, APOH, BIN2, C3, CLEC3B, CLU, CRISP3, DBH, ECM1, F13A1, GSN, HRG, ITIH4, KNG1, PLG, PROS1, SELENOP, SERPINA4, SERPINF2, SPP2, TTR	22	7,65E-16
**cytoplasmic vesicle lumen**	CC	A1BG, AHSG, APOH, BIN2, C3, CLEC3B, CLU, CRISP3, DBH, ECM1, F13A1, GSN, HRG, ITIH4, KNG1, PLG, PROS1, SELENOP, SERPINA4, SERPINF2, SPP2, TTR	22	8,46E-16
**vesicle lumen**	CC	A1BG, AHSG, APOH, BIN2, C3, CLEC3B, CLU, CRISP3, DBH, ECM1, F13A1, GSN, HRG, ITIH4, KNG1, PLG, PROS1, SELENOP, SERPINA4, SERPINF2, SPP2, TTR	22	8,46E-16
**high-density lipoprotein particle**	CC	APOA4, APOC1, APOC4, APOF, APOH, APOM, CETP, CLU, LCAT, PON1	10	6,51E-15

**endoplasmic reticulum lumen**	CC	ADAMTS13, AHSG, APOA4, APOB, BCHE, C3, CLU, F10, F2, IGFBP3, ITIH2, KNG1, MINPP1, PROC, PROZ, SERPINC1, SERPIND1, SPP2	18	8,69E-12
**platelet dense granule lumen**	CC	APOH, CLEC3B, ECM1, ITIH4, SELENOP, SERPINA4, SPP2	7	1,36E-11
**platelet alpha granule**	CC	A1BG, AHSG, CLU, F13A1, HRG, ITGB3, KNG1, PLG, PROS1, SERPINA5, SERPINF2	11	1,09E-10
**very-low-density lipoprotein particle**	CC	APOA4, APOB, APOC1, APOC4, APOH, APOM, PCYOX1	7	3,27E-10
**triglyceride-rich plasma lipoprotein particle**	CC	APOA4, APOB, APOC1, APOC4, APOH, APOM, PCYOX1	7	3,27E-10
**platelet dense granule**	CC	APOH, CLEC3B, ECM1, ITIH4, SELENOP, SERPINA4, SPP2	7	3,27E-10
**external side of plasma membrane**	CC	ACE, AZGP1, CDH13, COLEC10, ENG, ENPEP, F10, F2, FCN3, ITGB1, ITGB3, KIT, LAMP1, MCAM, NCAM1, PLG, SELL, SERPINA5	18	4,15E-10
**platelet alpha granule lumen**	CC	A1BG, AHSG, CLU, F13A1, HRG, KNG1, PLG, PROS1, SERPINF2	9	2,61E-09
**pore complex**	CC	C5, C6, C8A, C8B, C8G	5	4,70E-06

**chylomicron**	CC	APOA4, APOB, APOC1, APOH	4	8,97E-06
**low-density lipoprotein particle**	CC	APOA4, APOB, APOF, APOM	4	1,61E-05
**Golgi lumen**	CC	F10, F2, LUM, PROC, PROS1, PROZ, UMOD	7	3,23E-05
**collagen trimer**	CC	ADIPOQ, C1QA, C1QB, COLEC10, FCN3, LUM	6	0,000116854
**serine-type endopeptidase complex**	CC	CFH, COLEC10, FCN3	3	0,00169465
**focal adhesion**	CC	CDH13, DPP4, ENG, FAP, GSN, ITGB1, ITGB3, MCAM, MYH9, RAB10	10	0,001899637
**serine-type peptidase complex**	CC	CFH, COLEC10, FCN3	3	0,002112675
**cell-substrate junction**	CC	CDH13, DPP4, ENG, FAP, GSN, ITGB1, ITGB3, MCAM, MYH9, RAB10	10	0,002112675
**lamellipodium membrane**	CC	DPP4, FAP, ITGB3	3	0,00226476
**ruffle membrane**	CC	FAP, ITGB1, ITGB3, PLEK, PTPRJ	5	0,00255434
**leading edge membrane**	CC	DPP4, FAP, ITGB1, ITGB3, PLEK, PTPRJ	6	0,005532896
**ruffle**	CC	FAP, ITGB1, ITGB3, MYH9, PLEK, PTPRJ	6	0,005823916
**cell projection membrane**	CC	ACE, DPP4, FAP, ITGB1, ITGB3, PLEK, PTPRJ, UMOD	8	0,009033618
**secretory granule membrane**	CC	DBH, ITGB3, LAMP1, PTPRJ, RAB10, SELL, SERPINA5	7	0,019522125
**chromaffin granule**	CC	CLU, DBH	2	0,019522125
**brush border**	CC	ACE, ENPEP, MYH9, MYL12B	4	0,024857873
**vacuolar lumen**	CC	APOB, C3, GC, LUM, TTR	5	0,024857873
**cell leading edge**	CC	DPP4, FAP, GSN, ITGB1, ITGB3, MYH9, PLEK, PTPRJ	8	0,024857873
**melanosome**	CC	GPNMB, ITGB1, ITGB3, LAMP1	4	0,024857873
**pigment granule**	CC	GPNMB, ITGB1, ITGB3, LAMP1	4	0,024857873

**lamellipodium**	CC	DPP4, FAP, GSN, ITGB1, ITGB3	5	0,038781448
**peptidase complex**	CC	CFH, COLEC10, FAP, FCN3	4	0,038781448
**peptidase inhibitor activity**	MF	AHSG, C3, C5, FETUB, HRG, ITIH1, ITIH2, ITIH4, KNG1, PI16, PROS1, SERPINA4, SERPINA5, SERPINA6, SERPINA7, SERPINC1, SERPIND1, SERPINF2, SPP2	19	4,55E-16
**endopeptidase inhibitor activity**	MF	AHSG, C3, C5, FETUB, HRG, ITIH1, ITIH2, ITIH4, KNG1, PROS1, SERPINA4, SERPINA5, SERPINA6, SERPINA7, SERPINC1, SERPIND1, SERPINF2, SPP2	18	2,04E-15
**peptidase regulator activity**	MF	AHSG, C3, C5, FETUB, HRG, ITIH1, ITIH2, ITIH4, KNG1, PCOLCE, PI16, PROS1, SERPINA4, SERPINA5, SERPINA6, SERPINA7, SERPINC1, SERPIND1, SERPINF2, SPP2	20	2,04E-15

**endopeptidase regulator activity**	MF	AHSG, C3, C5, FETUB, HRG, ITIH1, ITIH2, ITIH4, KNG1, PROS1, SERPINA4, SERPINA5, SERPINA6, SERPINA7, SERPINC1, SERPIND1, SERPINF2, SPP2	18	9,21E-15
**glycosaminoglycan binding**	MF	APOB, APOH, CFH, CLEC3B, ENG, F11, F2, GPNMB, HABP2, HRG, ITIH1, ITIH2, KNG1, PCOLCE, PGLYRP2, SELL, SERPINA5, SERPINC1, SERPIND1	19	4,98E-14
**serine-type peptidase activity**	MF	ACE, C1RL, CFI, DPP4, F10, F11, F12, F2, FAP, HABP2, HGFAC, KLKB1, MASP1, PLG, PROC, PROZ	16	3,50E-12
**serine hydrolase activity**	MF	ACE, C1RL, CFI, DPP4, F10, F11, F12, F2, FAP, HABP2, HGFAC, KLKB1, MASP1, PLG, PROC, PROZ	16	4,12E-12
**serine-type endopeptidase activity**	MF	C1RL, CFI, DPP4, F10, F11, F12, F2, FAP, HABP2, HGFAC, KLKB1, MASP1, PLG, PROC, PROZ	15	1,12E-11

**enzyme inhibitor activity**	MF	AHSG, APOC1, C3, C5, FETUB, HRG, ITIH1, ITIH2, ITIH4, KNG1, PI16, PROS1, SERPINA4, SERPINA5, SERPINA6, SERPINA7, SERPINC1, SERPIND1, SERPINF2, SPP2	20	2,77E-11
**heparin binding**	MF	APOB, APOH, CFH, CLEC3B, F11, F2, GPNMB, HRG, KNG1, PCOLCE, SELL, SERPINA5, SERPINC1, SERPIND1	14	1,26E-10
**serine-type endopeptidase inhibitor activity**	MF	HRG, ITIH1, ITIH2, ITIH4, SERPINA4, SERPINA5, SERPINA6, SERPINA7, SERPINC1, SERPIND1, SERPINF2	11	9,01E-10
**protease binding**	MF	CHL1, DPP4, ECM1, FAP, ITGB1, ITGB3, KIT, PLG, SELL, SERPINA5, SERPINC1, SERPINF2	12	1,12E-09
**endopeptidase activity**	MF	ACE, ADAMTS13, ADGRG6, C1RL, CFI, DPP4, F10, F11, F12, F2, FAP, HABP2, HGFAC, KLKB1, MASP1, PLG, PROC, PROZ	18	1,30E-09
**sulfur compound binding**	MF	APOB, APOH, CFH, CLEC3B, F11, F2, GPNMB, HRG, KNG1, PCOLCE, SELL, SERPINA5, SERPINC1, SERPIND1	14	3,72E-08

**exopeptidase activity**	MF	ACE, CNDP1, CPB2, DPP4, ENPEP, F11, FAP	7	6,39E-05
**lipid transporter activity**	MF	APOA4, APOB, APOC4, APOD, APOF, APOM, CETP, RBP4	8	0,000257283
**phosphatidylcholine binding**	MF	APOA4, APOC1, CETP, SERPINA5	4	0,000527699
**steroid binding**	MF	APOD, APOF, CETP, GC, SERPINA6, SHBG	6	0,000847889
**carbohydrate binding**	MF	ATRN, CLEC3B, CNTN1, COLEC10, DBH, ENG, FCN3, ITIH1, SELL	9	0,000893233
**cysteine-type endopeptidase inhibitor activity**	MF	AHSG, FETUB, HRG, KNG1	4	0,000936657
**quaternary ammonium group binding**	MF	APOA4, APOC1, CETP, SERPINA5	4	0,001357626
**virus receptor activity**	MF	DPP4, ITGB1, ITGB3, LAMP1, NCAM1	5	0,001586869
**exogenous protein binding**	MF	DPP4, ITGB1, ITGB3, LAMP1, NCAM1	5	0,001611061
**insulin-like growth factor binding**	MF	IGFALS, IGFBP3, ITGB3	3	0,002288939
**alcohol binding**	MF	APOD, APOF, C8G, CETP, RBP4	5	0,002654511
**phospholipid binding**	MF	APOA4, APOB, APOC1, APOH, APOM, BIN2, CETP, F10, GSN, PON1, SERPINA5	11	0,002654511
**cholesterol transfer activity**	MF	APOA4, APOB, CETP	3	0,004154158
**integrin binding**	MF	ADAMTS13, FAP, GPNMB, ITGB1, ITGB3, MYH9	6	0,004798583
**complement binding**	MF	C8A, C8G, CFH	3	0,004906186
**coreceptor activity**	MF	ENG, IL1RAP, ITGB1, ITGB3	4	0,004906186
**metalloexopeptidase activity**	MF	ACE, CNDP1, CPB2, ENPEP	4	0,006776003
**fibronectin binding**	MF	IGFBP3, ITGB1, ITGB3	3	0,006841758
**collagen binding**	MF	ADGRG6, ITGB1, LUM, PCOLCE	4	0,007096925
**sterol transfer activity**	MF	APOA4, APOB, CETP	3	0,007096925

**growth factor binding**	MF	ENG, IGFALS, IGFBP3, ITGB3, KIT	5	0,010535385
**proteoglycan binding**	MF	CFH, GPNMB, HRG	3	0,011248671
**dipeptidyl-peptidase activity**	MF	DPP4, FAP	2	0,011248671
**retinoid binding**	MF	C8G, RBP4, SERPINA5	3	0,011248671
**isoprenoid binding**	MF	C8G, RBP4, SERPINA5	3	0,011248671
**aminopeptidase activity**	MF	DPP4, ENPEP, F11	3	0,012728906
**serine-type exopeptidase activity**	MF	ACE, F11	2	0,015352877
**vitamin binding**	MF	AFM, C8G, DBH, GC, RBP4	5	0,015368238
**insulin-like growth factor I binding**	MF	IGFBP3, ITGB3	2	0,017229929
**carboxypeptidase activity**	MF	ACE, CNDP1, CPB2	3	0,018399365
**platelet-derived growth factor receptor binding**	MF	ITGB3, PTPRJ	2	0,019129988
**retinol binding**	MF	C8G, RBP4	2	0,023956799
**misfolded protein binding**	MF	CLU, F12	2	0,023956799
**cholesterol binding**	MF	APOD, APOF, CETP	3	0,024997163
**dipeptidase activity**	MF	ACE, CNDP1	2	0,025936973
**apolipoprotein binding**	MF	LCAT, PLG	2	0,028479333
**metallopeptidase activity**	MF	ACE, ADAMTS13, CNDP1, CPB2, ENPEP	5	0,030481332
**heparan sulfate proteoglycan binding**	MF	CFH, HRG	2	0,030481332
**carboxylic acid binding**	MF	ADIPOQ, APOC1, DBH, SELL, SERPINA5	5	0,03100213
**copper ion binding**	MF	AOC3, APOA4, DBH	3	0,034159933
**lipid transfer activity**	MF	APOA4, APOB, CETP	3	0,034868013
**carboxylic ester hydrolase activity**	MF	BCHE, GPLD1, LCAT, PON1, PON3	5	0,034868013
**extracellular matrix binding**	MF	ADGRG6, ITGB1, ITGB3	3	0,035277724
**sialic acid binding**	MF	ADIPOQ, SELL	2	0,035960396
**organic acid binding**	MF	ADIPOQ, APOC1, DBH, SELL, SERPINA5	5	0,035960396
**sterol binding**	MF	APOD, APOF, CETP	3	0,037936866

**cell adhesion mediator activity**	MF	CNTN1, ITGB1, RAB10	3	0,038836224
**hyaluronic acid binding**	MF	ITIH1, ITIH2	2	0,040432947
**monosaccharide binding**	MF	COLEC10, DBH, ENG	3	0,045031029
**immunoglobulin binding**	MF	HRG, UMOD	2	0,045031029
**low-density lipoprotein particle receptor binding**	MF	APOB, CLU	2	0,045031029
**myosin binding**	MF	GSN, MYL12B, RAB10	3	0,045031029
**cytokine binding**	MF	ENG, ITGB1, ITGB3, KIT	4	0,047600121
**mitogen-activated protein kinase binding**	MF	ACE, PTPRJ	2	0,049677292

Table S5b. Showing enriched gene ontologies from downregulated DEPs in sepsis compared to control. BP=biological processes, CC=cellular compartments, MF=molecular functions. Ontologies classified as significantly altered if exhibiting an adjusted p-value < 0.05.

<u>Table S6a – Gene Ontology Pancreatitis vs Control Up</u>

Description	Ontology	geneID	Protein Count	Adjusted P-value
**acute-phase response**	BP	CRP, F8, LBP, ORM1, ORM2, SAA1, SAA2, SERPINA3	8	2,20E-10
**acute inflammatory response**	BP	CRP, F8, LBP, ORM1, ORM2, SAA1, SAA2, SERPINA3	8	1,07E-07
**regulation of inflammatory response**	BP	IL1RL1, LBP, MMP8, MMP9, PSMA1, PSMA6, S100A12, S100A8, S100A9, SAA1	10	2,50E-05
**collagen metabolic process**	BP	COL6A1, CTSB, MMP8, MMP9, PRSS2, VIM	6	6,76E-05
**respiratory burst involved in inflammatory response**	BP	LBP, PRDX2, S100A9	3	0,000316302
**neutrophil chemotaxis**	BP	LBP, S100A12, S100A8, S100A9, SAA1	5	0,000316398
**positive regulation of inflammatory response**	BP	IL1RL1, LBP, MMP8, S100A12, S100A8, S100A9	6	0,000355593
**collagen catabolic process**	BP	CTSB, MMP8, MMP9, PRSS2	4	0,000523259
**neutrophil migration**	BP	LBP, S100A12, S100A8, S100A9, SAA1	5	0,000687732
**respiratory burst involved in defense response**	BP	LBP, PRDX2, S100A9	3	0,000885594
**regulation of macrophage activation**	BP	IL1RL1, LBP, MMP8, VSIG4	4	0,00113625
**granulocyte chemotaxis**	BP	LBP, S100A12, S100A8, S100A9, SAA1	5	0,00113625
**leukocyte migration involved in inflammatory response**	BP	LBP, S100A8, S100A9	3	0,00113625
**extracellular matrix disassembly**	BP	LCP1, MMP8, MMP9, PRSS2	4	0,001311467
**response to lipopolysaccharide**	BP	COL6A1, LBP, MMP8, MMP9, PRDX2, S100A8, VIM	7	0,001412143
**regulation of endopeptidase activity**	BP	CTSD, MMP9, S100A8, S100A9, SERPINA3, TIMP1	6	0,0017707
**response to molecule of bacterial origin**	BP	COL6A1, LBP, MMP8, MMP9, PRDX2, S100A8, VIM	7	0,001801649

**granulocyte migration**	BP	LBP, S100A12, S100A8, S100A9, SAA1	5	0,002041955
**positive regulation of macrophage activation**	BP	IL1RL1, LBP, MMP8	3	0,00272215
**respiratory burst**	BP	LBP, PRDX2, S100A9	3	0,006102135
**positive regulation of tumor necrosis factor production**	BP	LBP, MMP8, ORM1, ORM2	4	0,006102135
**macrophage activation**	BP	IL1RL1, LBP, MMP8, VSIG4	4	0,006102135
**positive regulation of tumor necrosis factor superfamily cytokine production**	BP	LBP, MMP8, ORM1, ORM2	4	0,006102135
**extracellular matrix organization**	BP	COL6A1, LCP1, MMP8, MMP9, NID1, PRSS2	6	0,006102135
**extracellular structure organization**	BP	COL6A1, LCP1, MMP8, MMP9, NID1, PRSS2	6	0,006102135
**external encapsulating structure organization**	BP	COL6A1, LCP1, MMP8, MMP9, NID1, PRSS2	6	0,006102135
**astrocyte development**	BP	S100A8, S100A9, VIM	3	0,006102135
**regulation of peptidase activity**	BP	CTSD, MMP9, S100A8, S100A9, SERPINA3, TIMP1	6	0,006357859
**positive regulation of NF-kappaB transcription factor activity**	BP	PSMA6, S100A12, S100A8, S100A9	4	0,007327278
**cellular response to lipopolysaccharide**	BP	LBP, MMP8, MMP9, PRDX2, VIM	5	0,007327278
**endodermal cell differentiation**	BP	COL6A1, MMP8, MMP9	3	0,008678051
**leukocyte chemotaxis**	BP	LBP, S100A12, S100A8, S100A9, SAA1	5	0,008678051
**cellular response to molecule of bacterial origin**	BP	LBP, MMP8, MMP9, PRDX2, VIM	5	0,008678051
**myeloid leukocyte migration**	BP	LBP, S100A12, S100A8, S100A9, SAA1	5	0,008678051
**cellular response to UV-A**	BP	MMP9, TIMP1	2	0,008815081
**myeloid leukocyte activation**	BP	IL1RL1, LBP, MMP8, S100A12, VSIG4	5	0,008815081
**glial cell development**	BP	COL6A1, S100A8, S100A9, VIM	4	0,008815081

**glyceraldehyde-3-phosphate metabolic process**	BP	TALDO1, TKT	2	0,009841091
**regulation of cysteine-type endopeptidase activity involved in apoptotic process**	BP	CTSD, MMP9, S100A8, S100A9	4	0,010389922
**endoderm formation**	BP	COL6A1, MMP8, MMP9	3	0,010762747
**leukocyte aggregation**	BP	S100A8, S100A9	2	0,010762747
**cellular response to biotic stimulus**	BP	LBP, MMP8, MMP9, PRDX2, VIM	5	0,011190162
**response to UV-A**	BP	MMP9, TIMP1	2	0,011954045
**response to UV**	BP	CAT, COL6A1, MMP9, TIMP1	4	0,012599783
**defense response to fungus**	BP	S100A12, S100A8, S100A9	3	0,012599783
**protein nitrosylation**	BP	S100A8, S100A9	2	0,012599783
**peptidyl-cysteine S-nitrosylation**	BP	S100A8, S100A9	2	0,012599783
**wound healing**	BP	F8, LRG1, PRDX2, SAA1, TIMP1, VWF	6	0,014001442
**response to peptide hormone**	BP	CAT, COL6A1, CTSD, LTA4H, REG1B, TIMP1	6	0,014041784
**regulation of cysteine-type endopeptidase activity**	BP	CTSD, MMP9, S100A8, S100A9	4	0,014159409
**opsonization**	BP	CRP, LBP	2	0,014638727
**positive regulation of interleukin-1 production**	BP	ORM1, ORM2, SAA1	3	0,014638727
**pyridine nucleotide metabolic process**	BP	BPGM, COL6A1, TALDO1, TKT	4	0,014638727
**nicotinamide nucleotide metabolic process**	BP	BPGM, COL6A1, TALDO1, TKT	4	0,014638727
**humoral immune response**	BP	PRSS2, REG1B, S100A12, S100A9, VSIG4	5	0,015758538
**response to fungus**	BP	S100A12, S100A8, S100A9	3	0,015758538
**pyridine-containing compound metabolic process**	BP	BPGM, COL6A1, TALDO1, TKT	4	0,015758538
**regulation of respiratory burst**	BP	LBP, S100A9	2	0,016525685

**antimicrobial humoral response**	BP	PRSS2, REG1B, S100A12, S100A9	4	0,017431923
**pentose-phosphate shunt**	BP	TALDO1, TKT	2	0,017431923
**keratinocyte migration**	BP	LRG1, MMP9	2	0,017431923
**tumor necrosis factor production**	BP	LBP, MMP8, ORM1, ORM2	4	0,01746395
**regulation of tumor necrosis factor production**	BP	LBP, MMP8, ORM1, ORM2	4	0,01746395
**cell chemotaxis**	BP	LBP, S100A12, S100A8, S100A9, SAA1	5	0,017514797
**chronic inflammatory response**	BP	S100A8, S100A9	2	0,017514797
**regulation of integrin-mediated signaling pathway**	BP	NID1, TIMP1	2	0,017514797
**neuroinflammatory response**	BP	BPGM, MMP8, MMP9	3	0,017514797
**tumor necrosis factor superfamily cytokine production**	BP	LBP, MMP8, ORM1, ORM2	4	0,017514797
**regulation of tumor necrosis factor superfamily cytokine production**	BP	LBP, MMP8, ORM1, ORM2	4	0,017514797
**endoderm development**	BP	COL6A1, MMP8, MMP9	3	0,017514797
**lung development**	BP	CHI3L1, COL6A1, CTSZ, LTA4H	4	0,018763666
**astrocyte differentiation**	BP	S100A8, S100A9, VIM	3	0,018777935
**defense response to bacterium**	BP	CRP, LBP, S100A12, S100A8, S100A9	5	0,018909315
**NADPH regeneration**	BP	TALDO1, TKT	2	0,019045902
**respiratory tube development**	BP	CHI3L1, COL6A1, CTSZ, LTA4H	4	0,019119036
**positive regulation of DNA-binding transcription factor activity**	BP	PSMA6, S100A12, S100A8, S100A9	4	0,02100647
**positive regulation of cysteine-type endopeptidase activity involved in apoptotic process**	BP	CTSD, S100A8, S100A9	3	0,02279305

**cellular oxidant detoxification**	BP	CAT, PRDX2, S100A9	3	0,02279305
**interleukin-8 production**	BP	CHI3L1, CRP, LBP	3	0,02605707
**regulation of interleukin-8 production**	BP	CHI3L1, CRP, LBP	3	0,02605707
**respiratory system development**	BP	CHI3L1, COL6A1, CTSZ, LTA4H	4	0,02605707
**production of molecular mediator involved in inflammatory response**	BP	LBP, PRDX2, S100A9	3	0,02605707
**phagocytosis, recognition**	BP	CRP, LBP	2	0,02605707
**glucose 6-phosphate metabolic process**	BP	TALDO1, TKT	2	0,02605707
**lung cell differentiation**	BP	COL6A1, LTA4H	2	0,02605707
**lung epithelial cell differentiation**	BP	COL6A1, LTA4H	2	0,02605707
**negative regulation of endopeptidase activity**	BP	MMP9, SERPINA3, TIMP1	3	0,026893648
**blood coagulation**	BP	F8, PRDX2, SAA1, VWF	4	0,027851317
**hydrogen peroxide catabolic process**	BP	CAT, PRDX2	2	0,028749052
**positive regulation of cysteine-type endopeptidase activity**	BP	CTSD, S100A8, S100A9	3	0,029527817
**hemostasis**	BP	F8, PRDX2, SAA1, VWF	4	0,029629878
**coagulation**	BP	F8, PRDX2, SAA1, VWF	4	0,029629878
**reactive oxygen species metabolic process**	BP	CAT, COL6A1, CRP, PRDX2	4	0,029751586
**leukocyte migration**	BP	LBP, S100A12, S100A8, S100A9, SAA1	5	0,030360385
**regulation of cell growth**	BP	CDHR2, IGFBP4, PRSS2, S100A8, S100A9	5	0,031339863
**leukotriene metabolic process**	BP	CPA1, LTA4H	2	0,032007142
**peptidyl-cysteine modification**	BP	S100A8, S100A9	2	0,035159683
**cellular detoxification**	BP	CAT, PRDX2, S100A9	3	0,035159683
**carbohydrate derivative catabolic process**	BP	BPGM, CHI3L1, COL6A1, MAN1A1	4	0,035159683

**intracellular zinc ion homeostasis**	BP	S100A8, S100A9	2	0,036309905
**leukocyte cell-cell adhesion**	BP	LRG1, PRDX2, S100A8, S100A9, VSIG4	5	0,036653577
**regulation of toll-like receptor signaling pathway**	BP	S100A8, S100A9	2	0,036653577
**basement membrane organization**	BP	COL6A1, NID1	2	0,036653577
**interleukin-1 production**	BP	ORM1, ORM2, SAA1	3	0,036653577
**regulation of interleukin-1 production**	BP	ORM1, ORM2, SAA1	3	0,036653577
**glial cell differentiation**	BP	COL6A1, S100A8, S100A9, VIM	4	0,03710416
**formation of primary germ layer**	BP	COL6A1, MMP8, MMP9	3	0,037291479
**detection of biotic stimulus**	BP	CDHR2, LBP	2	0,037291479
**response to toxic substance**	BP	CAT, COL6A1, PRDX2, S100A9	4	0,037543505
**cellular response to toxic substance**	BP	CAT, PRDX2, S100A9	3	0,03888695
**antimicrobial humoral immune response mediated by antimicrobial peptide**	BP	REG1B, S100A12, S100A9	3	0,039339585
**regulation of neuroinflammatory response**	BP	MMP8, MMP9	2	0,039442012
**positive regulation of endopeptidase activity**	BP	CTSD, S100A8, S100A9	3	0,039442012
**cellular component disassembly**	BP	COL6A1, LCP1, MMP8, MMP9, PRSS2	5	0,039626804
**lung epithelium development**	BP	COL6A1, LTA4H	2	0,046360476
**positive regulation of peptidase activity**	BP	CTSD, S100A8, S100A9	3	0,046743102
**chemotaxis**	BP	LBP, S100A12, S100A8, S100A9, SAA1	5	0,047163041
**NADP metabolic process**	BP	TALDO1, TKT	2	0,047163041
**taxis**	BP	LBP, S100A12, S100A8, S100A9, SAA1	5	0,047206373

**secretory granule lumen**	CC	CAT, CHI3L1, CTSD, CTSZ, F8, LRG1, MMP8, ORM1, ORM2, PRSS2, PSMB7, S100A12, S100A8, S100A9, SERPINA3, TIMP1, VWF	17	1,42E-17
**cytoplasmic vesicle lumen**	CC	CAT, CHI3L1, CTSD, CTSZ, F8, LRG1, MMP8, ORM1, ORM2, PRSS2, PSMB7, S100A12, S100A8, S100A9, SERPINA3, TIMP1, VWF	17	1,42E-17
**vesicle lumen**	CC	CAT, CHI3L1, CTSD, CTSZ, F8, LRG1, MMP8, ORM1, ORM2, PRSS2, PSMB7, S100A12, S100A8, S100A9, SERPINA3, TIMP1, VWF	17	1,42E-17
**collagen-containing extracellular matrix**	CC	COL6A1, CTSB, CTSD, CTSZ, MMP8, MMP9, NID1, ORM1, ORM2, S100A8, S100A9, SERPINA3, TIMP1, VWF	14	1,32E-11
**specific granule lumen**	CC	CHI3L1, CTSD, CTSZ, LRG1, MMP8, ORM1, ORM2	7	2,11E-09
**ficolin-1-rich granule lumen**	CC	CAT, CTSB, CTSD, CTSZ, LRG1, LTA4H, MMP9, PSMB7	8	6,93E-09
**tertiary granule lumen**	CC	CTSD, LRG1, LTA4H, MMP8, MMP9, ORM1	6	4,47E-08
**ficolin-1-rich granule**	CC	CAT, CTSB, CTSD, CTSZ, LRG1, LTA4H, MMP9, PSMB7	8	1,17E-07
**platelet alpha granule lumen**	CC	F8, ORM1, ORM2, SERPINA3, TIMP1, VWF	6	1,17E-07
**platelet alpha granule**	CC	F8, ORM1, ORM2, SERPINA3, TIMP1, VWF	6	6,76E-07
**specific granule**	CC	CHI3L1, CTSD, CTSZ, LRG1, MMP8, ORM1, ORM2	7	7,67E-07
**tertiary granule**	CC	CTSD, LRG1, LTA4H, MMP8, MMP9, ORM1	6	1,84E-05

**endoplasmic reticulum lumen**	CC	APOA5, COL6A1, CTSZ, F8, IGFBP4, NUCB1, TIMP1	7	5,97E-05
**proteasome core complex**	CC	PSMA1, PSMA6, PSMB7	3	9,98E-05
**high-density lipoprotein particle**	CC	APOA5, SAA1, SAA2	3	0,000236369
**vacuolar lumen**	CC	CTSB, CTSD, ORM2, PRSS2, SERPINA3	5	0,000373655
**plasma lipoprotein particle**	CC	APOA5, SAA1, SAA2	3	0,000514866
**lipoprotein particle**	CC	APOA5, SAA1, SAA2	3	0,000514866
**protein-lipid complex**	CC	APOA5, SAA1, SAA2	3	0,00061723
**endoplasmic reticulum-Golgi intermediate compartment**	CC	CTSZ, F8, MAN1A1, NUCB1	4	0,001441161
**proteasome complex**	CC	PSMA1, PSMA6, PSMB7	3	0,002265608
**azurophil granule lumen**	CC	ORM2, PRSS2, SERPINA3	3	0,005960351
**basement membrane**	CC	COL6A1, NID1, TIMP1	3	0,005960351
**endopeptidase complex**	CC	PSMA1, PSMA6, PSMB7	3	0,006851705
**peptidase complex**	CC	PSMA1, PSMA6, PSMB7	3	0,014381388
**endosome lumen**	CC	CTSB, CTSD	2	0,014659162
**blood microparticle**	CC	ORM1, ORM2, SERPINA3	3	0,017538691
**peroxisome**	CC	CAT, TKT, VIM	3	0,017538691
**microbody**	CC	CAT, TKT, VIM	3	0,017538691
**primary lysosome**	CC	ORM2, PRSS2, SERPINA3	3	0,019374906
**azurophil granule**	CC	ORM2, PRSS2, SERPINA3	3	0,019374906
**collagen binding**	MF	COL6A1, CTSB, MMP9, NID1, VWF	5	0,000100477
**RAGE receptor binding**	MF	S100A12, S100A8, S100A9	3	0,000158884
**endopeptidase activity**	MF	CTSB, CTSD, CTSZ, MMP8, MMP9, PRSS2, PSMA6, PSMB7	8	0,000260913
**metallopeptidase activity**	MF	CPA1, LTA4H, MMP8, MMP9, PRSS2	5	0,00309335
**Toll-like receptor binding**	MF	S100A8, S100A9	2	0,012748451
**extracellular matrix structural constituent**	MF	CHI3L1, COL6A1, NID1, VWF	4	0,015752396
**long-chain fatty acid binding**	MF	S100A8, S100A9	2	0,015752396

**calcium-dependent protein binding**	MF	S100A12, S100A8, S100A9	3	0,015752396
**cysteine-type endopeptidase activity**	MF	CTSB, CTSD, CTSZ	3	0,015752396
**opsonin binding**	MF	CRP, VSIG4	2	0,015752396
**antioxidant activity**	MF	CAT, PRDX2, S100A9	3	0,015752396
**complement binding**	MF	CRP, VSIG4	2	0,020019344
**low-density lipoprotein particle receptor binding**	MF	APOA5, CRP	2	0,020019344
**exopeptidase activity**	MF	CPA1, CTSZ, LTA4H	3	0,02077495
**metalloendopeptidase activity**	MF	MMP8, MMP9, PRSS2	3	0,023091608
**lipoprotein particle receptor binding**	MF	APOA5, CRP	2	0,023091608
**proteoglycan binding**	MF	CTSB, NID1	2	0,030837826
**quaternary ammonium group binding**	MF	APOA5, TKT	2	0,033760771
**lipopolysaccharide binding**	MF	LBP, PSMA1	2	0,033760771
**carbohydrate binding**	MF	CHI3L1, GALNT2, REG1B, TALDO1	4	0,03430252
**carboxypeptidase activity**	MF	CPA1, CTSZ	2	0,038080168
**fatty acid binding**	MF	S100A8, S100A9	2	0,038080168

Table S6a. Showing enriched gene ontologies from upregulated DEPs in pancreatitis compared to control. BP=biological processes, CC=cellular compartments, MF=molecular functions. Ontologies classified as significantly altered if exhibiting an adjusted p-value < 0.05.

<u>Table S6b – Gene Ontology Pancreatitis vs Control Down</u>

Description	Ontology	geneID	Protein Count	Adjusted P-value
**regulation of fibrinolysis**	BP	F12, FAP, HRG, KLKB1	4	3,44E-05
**negative regulation of peptidase activity**	BP	ECM1, FETUB, ITIH1, ITIH2, SERPINA4, SERPINA5, SERPIND1	7	3,78E-05
**positive regulation of protein processing**	BP	CLEC3B, F12, GSN, KLKB1	4	3,78E-05
**fibrinolysis**	BP	F12, FAP, HRG, KLKB1	4	3,78E-05
**positive regulation of protein maturation**	BP	CLEC3B, F12, GSN, KLKB1	4	3,78E-05
**regulation of peptidase activity**	BP	ECM1, FETUB, GSN, ITIH1, ITIH2, SERPINA4, SERPINA5, SERPIND1	8	3,78E-05
**blood coagulation**	BP	F12, F13A1, FAP, HGFAC, HRG, KLKB1, SERPIND1	7	3,78E-05
**hemostasis**	BP	F12, F13A1, FAP, HGFAC, HRG, KLKB1, SERPIND1	7	3,78E-05
**coagulation**	BP	F12, F13A1, FAP, HGFAC, HRG, KLKB1, SERPIND1	7	3,78E-05
**negative regulation of hydrolase activity**	BP	ECM1, FETUB, ITIH1, ITIH2, SERPINA4, SERPINA5, SERPIND1	7	4,60E-05
**negative regulation of proteolysis**	BP	ECM1, FETUB, ITIH1, ITIH2, SERPINA4, SERPINA5, SERPIND1	7	0,000143413
**negative regulation of blood coagulation**	BP	F12, FAP, HRG, KLKB1	4	0,000206271
**negative regulation of hemostasis**	BP	F12, FAP, HRG, KLKB1	4	0,000206321
**negative regulation of coagulation**	BP	F12, FAP, HRG, KLKB1	4	0,000241442
**zymogen activation**	BP	CLEC3B, F12, HGFAC, KLKB1	4	0,000300638
**protein processing**	BP	ACE, CLEC3B, F12, GSN, HGFAC, KLKB1	6	0,000396342

**protein activation cascade**	BP	F12, F13A1, KLKB1	3	0,000396342
**regulation of body fluid levels**	BP	F12, F13A1, FAP, HGFAC, HRG, KLKB1, SERPIND1	7	0,000396342
**regulation of protein processing**	BP	CLEC3B, F12, GSN, KLKB1	4	0,000423453
**regulation of blood coagulation**	BP	F12, FAP, HRG, KLKB1	4	0,000452466
**regulation of hemostasis**	BP	F12, FAP, HRG, KLKB1	4	0,000482977
**regulation of protein maturation**	BP	CLEC3B, F12, GSN, KLKB1	4	0,000515007
**regulation of coagulation**	BP	F12, FAP, HRG, KLKB1	4	0,000578248
**negative regulation of wound healing**	BP	F12, FAP, HRG, KLKB1	4	0,000614404
**plasminogen activation**	BP	CLEC3B, F12, KLKB1	3	0,000724751
**wound healing**	BP	F12, F13A1, FAP, HGFAC, HRG, KLKB1, SERPIND1	7	0,00073923
**negative regulation of catalytic activity**	BP	ECM1, FETUB, ITIH1, ITIH2, SERPINA4, SERPINA5, SERPIND1	7	0,001268936
**negative regulation of response to wounding**	BP	F12, FAP, HRG, KLKB1	4	0,001459976
**negative regulation of endopeptidase activity**	BP	FETUB, SERPINA4, SERPINA5, SERPIND1	4	0,001899481
**regulation of endopeptidase activity**	BP	FETUB, GSN, SERPINA4, SERPINA5, SERPIND1	5	0,003541566
**regulation of wound healing**	BP	F12, FAP, HRG, KLKB1	4	0,00468999
**peripheral nervous system axon regeneration**	BP	APOA4, APOD	2	0,00483189
**regulation of triglyceride catabolic process**	BP	APOA4, GPLD1	2	0,0080916
**positive regulation of proteolysis**	BP	CLEC3B, F12, GPLD1, GSN, KLKB1	5	0,010943162
**regulation of response to wounding**	BP	F12, FAP, HRG, KLKB1	4	0,011072057
**blood coagulation, fibrin clot formation**	BP	F12, F13A1	2	0,011369264

**regulation of plasminogen activation**	BP	CLEC3B, F12	2	0,014069441
**negative regulation of bone mineralization**	BP	AHSG, ECM1	2	0,018733702
**positive regulation of triglyceride metabolic process**	BP	APOA4, GPLD1	2	0,020054061
**regulation of cell-substrate adhesion**	BP	ABI3BP, APOD, CDH13, HRG	4	0,021192554
**positive regulation of blood coagulation**	BP	F12, HRG	2	0,027818066
**positive regulation of hemostasis**	BP	F12, HRG	2	0,027818066
**acute inflammatory response**	BP	AHSG, F12, KLKB1	3	0,027818066
**glycosaminoglycan metabolic process**	BP	ITIH1, ITIH2, PGLYRP2	3	0,027903666
**triglyceride catabolic process**	BP	APOA4, GPLD1	2	0,02823175
**negative regulation of biomineral tissue development**	BP	AHSG, ECM1	2	0,031704796
**positive regulation of coagulation**	BP	F12, HRG	2	0,033129603
**aminoglycan metabolic process**	BP	ITIH1, ITIH2, PGLYRP2	3	0,033548386
**ossification**	BP	AHSG, CLEC3B, ECM1, GPLD1, IGFBP3	5	0,034523308
**regulation of cell-matrix adhesion**	BP	APOD, CDH13, HRG	3	0,034523308
**positive regulation of cell-substrate adhesion**	BP	ABI3BP, CDH13, HRG	3	0,034625612
**bone mineralization**	BP	AHSG, CLEC3B, ECM1	3	0,034660388
**hyaluronan metabolic process**	BP	ITIH1, ITIH2	2	0,034660388
**negative regulation of cell junction assembly**	BP	ACE, APOD	2	0,034660388
**endothelial cell migration**	BP	CDH13, FAP, GPLD1, HRG	4	0,038931915
**detection of biotic stimulus**	BP	FAP, PGLYRP2	2	0,042363751
**neutral lipid catabolic process**	BP	APOA4, GPLD1	2	0,042363751
**acylglycerol catabolic process**	BP	APOA4, GPLD1	2	0,042363751

**negative regulation of ossification**	BP	AHSG, ECM1	2	0,045972955
**negative regulation of cell adhesion**	BP	APOD, CDH13, GPNMB, HRG	4	0,049347306
**collagen-containing extracellular matrix**	CC	ABI3BP, AHSG, APOA4, CDH13, CLEC3B, ECM1, F12, F13A1, HRG, ITIH1, ITIH2, NCAM1, SERPINA5	13	3,45E-11
**blood microparticle**	CC	AFM, AHSG, APOA4, F13A1, GSN, HRG, ITIH1, ITIH2	8	5,37E-09
**secretory granule lumen**	CC	ADA2, AHSG, CLEC3B, CRISP3, ECM1, F13A1, GSN, HRG, SELENOP, SERPINA4	10	5,37E-09
**cytoplasmic vesicle lumen**	CC	ADA2, AHSG, CLEC3B, CRISP3, ECM1, F13A1, GSN, HRG, SELENOP, SERPINA4	10	5,37E-09
**vesicle lumen**	CC	ADA2, AHSG, CLEC3B, CRISP3, ECM1, F13A1, GSN, HRG, SELENOP, SERPINA4	10	5,37E-09
**platelet dense granule lumen**	CC	CLEC3B, ECM1, SELENOP, SERPINA4	4	1,41E-07
**platelet dense granule**	CC	CLEC3B, ECM1, SELENOP, SERPINA4	4	7,18E-07
**platelet alpha granule**	CC	AHSG, F13A1, HRG, SERPINA5	4	0,000255803
**platelet alpha granule lumen**	CC	AHSG, F13A1, HRG	3	0,002314778
**endoplasmic reticulum lumen**	CC	AHSG, APOA4, IGFBP3, ITIH2, SERPIND1	5	0,002314778
**external side of plasma membrane**	CC	ACE, CDH13, NCAM1, SELL, SERPINA5	5	0,00684829
**rough endoplasmic reticulum**	CC	F12, HGFAC	2	0,037104849
**glycosaminoglycan binding**	MF	ADA2, CLEC3B, GPNMB, HRG, ITIH1, ITIH2, PGLYRP2, SELL, SERPINA5, SERPIND1	10	3,69E-09
**endopeptidase inhibitor activity**	MF	AHSG, FETUB, HRG, ITIH1, ITIH2, SERPINA4, SERPINA5, SERPIND1	8	6,01E-08

**peptidase inhibitor activity**	MF	AHSG, FETUB, HRG, ITIH1, ITIH2, SERPINA4, SERPINA5, SERPIND1	8	6,01E-08
**endopeptidase regulator activity**	MF	AHSG, FETUB, HRG, ITIH1, ITIH2, SERPINA4, SERPINA5, SERPIND1	8	7,92E-08
**peptidase regulator activity**	MF	AHSG, FETUB, HRG, ITIH1, ITIH2, SERPINA4, SERPINA5, SERPIND1	8	3,50E-07
**serine-type endopeptidase inhibitor activity**	MF	HRG, ITIH1, ITIH2, SERPINA4, SERPINA5, SERPIND1	6	7,57E-07
**heparin binding**	MF	ADA2, CLEC3B, GPNMB, HRG, SELL, SERPINA5, SERPIND1	7	7,57E-07
**sulfur compound binding**	MF	ADA2, CLEC3B, GPNMB, HRG, SELL, SERPINA5, SERPIND1	7	1,38E-05
**enzyme inhibitor activity**	MF	AHSG, FETUB, HRG, ITIH1, ITIH2, SERPINA4, SERPINA5, SERPIND1	8	1,38E-05
**protease binding**	MF	CHL1, ECM1, FAP, SELL, SERPINA5	5	7,72E-05
**serine-type peptidase activity**	MF	ACE, F12, FAP, HGFAC, KLKB1	5	0,000399724
**serine hydrolase activity**	MF	ACE, F12, FAP, HGFAC, KLKB1	5	0,000403684
**cysteine-type endopeptidase inhibitor activity**	MF	AHSG, FETUB, HRG	3	0,000424322
**proteoglycan binding**	MF	ADA2, GPNMB, HRG	3	0,000428196
**carbohydrate binding**	MF	ATRN, CLEC3B, CNTN1, ITIH1, SELL	5	0,001610289
**serine-type endopeptidase activity**	MF	F12, FAP, HGFAC, KLKB1	4	0,002900575
**dipeptidase activity**	MF	ACE, CNDP1	2	0,003396334
**hyaluronic acid binding**	MF	ITIH1, ITIH2	2	0,006399086
**exopeptidase activity**	MF	ACE, CNDP1, FAP	3	0,006399086

**endopeptidase activity**	MF	ACE, F12, FAP, HGFAC, KLKB1	5	0,006399086
**phosphatidylcholine binding**	MF	APOA4, SERPINA5	2	0,008653098
**hydrolase activity, acting on carbon-nitrogen bonds**	MF	ADA2, BTD, PGLYRP2	3	0,012312174
**quaternary ammonium group binding**	MF	APOA4, SERPINA5	2	0,013991902
**carboxypeptidase activity**	MF	ACE, CNDP1	2	0,018422734
**metalloexopeptidase activity**	MF	ACE, CNDP1	2	0,035288508
**hydrolase activity, acting on carbon-nitrogen bonds, in linear amides**	MF	BTD, PGLYRP2	2	0,038984086

Table S6b. Showing enriched gene ontologies from downregulated DEPs in pancreatitis compared to control. BP=biological processes, CC=cellular compartments, MF=molecular functions. Ontologies classified as significantly altered if exhibiting an adjusted p-value < 0.05.

Table S7a – Gene Ontology Sepsis vs Pancreatitis Up

Description	Ontology	geneID	Protein Count	Adjusted P-value
**secretory granule lumen**	CC	COTL1, HSP90AA1, SERPINB1	3	0,002834878
**cytoplasmic vesicle lumen**	CC	COTL1, HSP90AA1, SERPINB1	3	0,002834878
**vesicle lumen**	CC	COTL1, HSP90AA1, SERPINB1	3	0,002834878
**collagen-containing extracellular matrix**	CC	HSP90AA1, PSAP, SERPINB1	3	0,00464278
**lysosomal lumen**	CC	HSP90AA1, PSAP	2	0,005239032
**melanosome**	CC	GANAB, HSP90AA1	2	0,005239032
**pigment granule**	CC	GANAB, HSP90AA1	2	0,005239032
**ficolin-1-rich granule lumen**	CC	COTL1, HSP90AA1	2	0,005612712
**site of polarized growth**	CC	COTL1, HSP90AA1	2	0,009017718
**vacuolar lumen**	CC	HSP90AA1, PSAP	2	0,009017718
**ficolin-1-rich granule**	CC	COTL1, HSP90AA1	2	0,009017718
**dendrite terminus**	CC	HSP90AA1	1	0,019981997
**cytolytic granule**	CC	SERPINB1	1	0,019981997
**sperm plasma membrane**	CC	HSP90AA1	1	0,019981997
**cell cortex**	CC	COTL1, MARCKS	2	0,019981997
**female germ cell nucleus**	CC	MARCKS	1	0,021050864
**endocytic vesicle**	CC	CD163, HSP90AA1	2	0,021050864
**endocytic vesicle lumen**	CC	HSP90AA1	1	0,025458436
**axonal growth cone**	CC	HSP90AA1	1	0,031425866
**myelin sheath**	CC	HSP90AA1	1	0,047638326
**scaffold protein binding**	MF	CD163, HSP90AA1, PSAP	3	8,13E-05
**actin filament binding**	MF	COTL1, MARCKS	2	0,042244076
**glycosphingolipid binding**	MF	PSAP	1	0,042244076
**glucosidase activity**	MF	GANAB	1	0,042244076
**ceramide binding**	MF	PSAP	1	0,042244076
**DNA polymerase binding**	MF	HSP90AA1	1	0,042244076
**scavenger receptor activity**	MF	CD163	1	0,042244076

**MHC class II protein complex binding**	MF	HSP90AA1	1	0,042244076
**sphingolipid binding**	MF	PSAP	1	0,042244076
**disordered domain specific binding**	MF	HSP90AA1	1	0,042244076
**actin binding**	MF	COTL1, MARCKS	2	0,042244076
**glycolipid binding**	MF	PSAP	1	0,042244076
**MHC protein complex binding**	MF	HSP90AA1	1	0,049687986
**ATP-dependent protein folding chaperone**	MF	HSP90AA1	1	0,049855891
**tau protein binding**	MF	HSP90AA1	1	0,049998069

Table S7a. Showing enriched gene ontologies from upregulated DEPs in sepsis compared to pancreatitis. BP=biological processes, CC=cellular compartments, MF=molecular functions. Ontologies classified as significantly altered if exhibiting an adjusted p-value < 0.05.

Table S7b – Gene Ontology Sepsis vs Pancreatitis Down

Description	Ontology	geneID	Protein Count	Adjusted P-value
**blood coagulation**	BP	APOE, APOH, F2, F9, PLG, PRDX2, PROC, PTPRJ	8	3,56E-08
**regulation of blood coagulation**	BP	APOE, APOH, F2, PLG, PRDX2, PROC	6	3,56E-08
**regulation of hemostasis**	BP	APOE, APOH, F2, PLG, PRDX2, PROC	6	3,56E-08
**hemostasis**	BP	APOE, APOH, F2, F9, PLG, PRDX2, PROC, PTPRJ	8	3,56E-08
**coagulation**	BP	APOE, APOH, F2, F9, PLG, PRDX2, PROC, PTPRJ	8	3,56E-08
**regulation of coagulation**	BP	APOE, APOH, F2, PLG, PRDX2, PROC	6	4,29E-08
**regulation of lipase activity**	BP	APOA5, APOC2, APOC3, APOH, SNCA	5	6,28E-08
**triglyceride-rich lipoprotein particle remodeling**	BP	APOA5, APOC2, APOC3, APOE	4	7,92E-08
**very-low-density lipoprotein particle remodeling**	BP	APOA5, APOC2, APOC3, APOE	4	7,92E-08
**wound healing**	BP	APOE, APOH, F2, F9, LRG1, PLG, PRDX2, PROC, PTPRJ	9	7,92E-08
**phospholipid efflux**	BP	APOA5, APOC2, APOC3, APOE	4	1,12E-07
**regulation of lipoprotein lipase activity**	BP	APOA5, APOC2, APOC3, APOH	4	1,12E-07
**triglyceride homeostasis**	BP	APOA5, APOC2, APOC3, APOC4, APOE	5	1,12E-07
**acylglycerol homeostasis**	BP	APOA5, APOC2, APOC3, APOC4, APOE	5	1,17E-07
**negative regulation of blood coagulation**	BP	APOE, APOH, F2, PLG, PROC	5	1,89E-07
**negative regulation of hemostasis**	BP	APOE, APOH, F2, PLG, PROC	5	1,96E-07
**negative regulation of coagulation**	BP	APOE, APOH, F2, PLG, PROC	5	2,48E-07

**regulation of body fluid levels**	BP	APOE, APOH, F2, F9, PLG, PRDX2, PROC, PTPRJ	8	3,67E-07
**neutral lipid metabolic process**	BP	APOA5, APOC2, APOC3, APOE, APOH, SNCA	6	4,67E-07
**regulation of wound healing**	BP	APOE, APOH, F2, PLG, PRDX2, PROC	6	5,28E-07
**positive regulation of blood coagulation**	BP	APOH, F2, PLG, PRDX2	4	1,14E-06
**positive regulation of hemostasis**	BP	APOH, F2, PLG, PRDX2	4	1,14E-06
**negative regulation of wound healing**	BP	APOE, APOH, F2, PLG, PROC	5	1,20E-06
**positive regulation of coagulation**	BP	APOH, F2, PLG, PRDX2	4	1,87E-06
**regulation of response to wounding**	BP	APOE, APOH, F2, PLG, PRDX2, PROC	6	1,96E-06
**protein-lipid complex remodeling**	BP	APOA5, APOC2, APOC3, APOE	4	2,16E-06
**plasma lipoprotein particle remodeling**	BP	APOA5, APOC2, APOC3, APOE	4	2,16E-06
**protein-containing complex remodeling**	BP	APOA5, APOC2, APOC3, APOE	4	2,67E-06
**negative regulation of response to wounding**	BP	APOE, APOH, F2, PLG, PROC	5	3,52E-06
**triglyceride metabolic process**	BP	APOA5, APOC2, APOC3, APOE, APOH	5	4,76E-06
**regulation of triglyceride metabolic process**	BP	APOA5, APOC2, APOC3, APOE	4	7,44E-06
**plasma lipoprotein particle organization**	BP	APOA5, APOC2, APOC3, APOE	4	1,01E-05
**protein-lipid complex organization**	BP	APOA5, APOC2, APOC3, APOE	4	1,18E-05
**regulation of triglyceride catabolic process**	BP	APOA5, APOC2, APOC3	3	1,18E-05
**negative regulation of fibrinolysis**	BP	APOH, F2, PLG	3	1,18E-05
**acylglycerol metabolic process**	BP	APOA5, APOC2, APOC3, APOE, APOH	5	1,21E-05

**lipid transport**	BP	APOA5, APOC2, APOC3, APOC4, APOE, APOF, APOH	7	1,68E-05
**positive regulation of wound healing**	BP	APOH, F2, PLG, PRDX2	4	2,64E-05
**regulation of endocytosis**	BP	APOA5, APOC2, APOC3, APOE, PTPRJ, SNCA	6	2,83E-05
**high-density lipoprotein particle clearance**	BP	APOC2, APOC3, APOE	3	2,87E-05
**regulation of fibrinolysis**	BP	APOH, F2, PLG	3	2,87E-05
**cholesterol efflux**	BP	APOA5, APOC2, APOC3, APOE	4	3,79E-05
**reverse cholesterol transport**	BP	APOC2, APOC3, APOE	3	3,82E-05
**lipid homeostasis**	BP	APOA5, APOC2, APOC3, APOC4, APOE	5	4,11E-05
**regulation of lipid metabolic process**	BP	APOA5, APOC2, APOC3, APOE, F2, SNCA	6	4,75E-05
**positive regulation of lipase activity**	BP	APOA5, APOC2, APOH	3	4,81E-05
**positive regulation of response to wounding**	BP	APOH, F2, PLG, PRDX2	4	4,87E-05
**regulation of plasma lipoprotein particle levels**	BP	APOA5, APOC2, APOC3, APOE	4	7,61E-05
**fibrinolysis**	BP	APOH, F2, PLG	3	8,55E-05
**triglyceride catabolic process**	BP	APOA5, APOC2, APOC3	3	9,38E-05
**lipoprotein metabolic process**	BP	APOA5, APOC2, APOC3, APOE	4	0,000112904
**negative regulation of sterol transport**	BP	APOC2, APOC3, APOE	3	0,000119674
**negative regulation of cholesterol transport**	BP	APOC2, APOC3, APOE	3	0,000119674
**phospholipid transport**	BP	APOA5, APOC2, APOC3, APOE	4	0,000119674
**cholesterol homeostasis**	BP	APOA5, APOC2, APOC3, APOE	4	0,000122013
**sterol homeostasis**	BP	APOA5, APOC2, APOC3, APOE	4	0,000124393

**regulation of fatty acid metabolic process**	BP	APOA5, APOC2, APOC3, SNCA	4	0,000126815
**negative regulation of response to external stimulus**	BP	APOE, APOH, F2, PLG, PRDX2, PROC	6	0,000145974
**cholesterol transport**	BP	APOA5, APOC2, APOC3, APOE	4	0,000205198
**neutral lipid catabolic process**	BP	APOA5, APOC2, APOC3	3	0,000212664
**acylglycerol catabolic process**	BP	APOA5, APOC2, APOC3	3	0,000212664
**receptor-mediated endocytosis**	BP	APOA5, APOC2, APOC3, APOE, SNCA	5	0,000230445
**sterol transport**	BP	APOA5, APOC2, APOC3, APOE	4	0,000250046
**positive regulation of lipid metabolic process**	BP	APOA5, APOC2, APOE, F2	4	0,000250046
**organic hydroxy compound transport**	BP	APOA5, APOC2, APOC3, APOE, SNCA	5	0,000293547
**negative regulation of lipid transport**	BP	APOC2, APOC3, APOE	3	0,000393282
**regulation of cellular ketone metabolic process**	BP	APOA5, APOC2, APOC3, SNCA	4	0,000420711
**positive regulation of small molecule metabolic process**	BP	APOA5, APOC2, APOE, SNCA	4	0,000420711
**positive regulation of endocytosis**	BP	APOA5, APOE, PTPRJ, SNCA	4	0,000482101
**regulation of platelet activation**	BP	APOE, F2, PTPRJ	3	0,000490341
**regulation of fatty acid biosynthetic process**	BP	APOA5, APOC2, APOC3	3	0,000490341
**plasma lipoprotein particle clearance**	BP	APOC2, APOC3, APOE	3	0,000511043
**regulation of small molecule metabolic process**	BP	APOA5, APOC2, APOC3, APOE, SNCA	5	0,000605865
**organophosphate ester transport**	BP	APOA5, APOC2, APOC3, APOE	4	0,000625279
**glycerolipid catabolic process**	BP	APOA5, APOC2, APOC3	3	0,000841788
**regulation of lipid localization**	BP	APOC2, APOC3, APOC4, APOE	4	0,000841788

**regulation of lipid catabolic process**	BP	APOA5, APOC2, APOC3	3	0,000864743
**negative regulation of lipid localization**	BP	APOC2, APOC3, APOE	3	0,000892386
**glycerolipid metabolic process**	BP	APOA5, APOC2, APOC3, APOE, APOH	5	0,00090488
**regulation of lipid biosynthetic process**	BP	APOA5, APOC2, APOC3, APOE	4	0,000940264
**regulation of sterol transport**	BP	APOC2, APOC3, APOE	3	0,001230659
**regulation of cholesterol transport**	BP	APOC2, APOC3, APOE	3	0,001230659
**negative regulation of platelet-derived growth factor receptor signaling pathway**	BP	PTPRJ, SNCA	2	0,00129087
**positive regulation of cellular catabolic process**	BP	APOA5, APOC2, APOE, SNCA	4	0,001805733
**cellular ketone metabolic process**	BP	APOA5, APOC2, APOC3, SNCA	4	0,001843612
**positive regulation of lipid biosynthetic process**	BP	APOA5, APOC2, APOE	3	0,002070105
**Cdc42 protein signal transduction**	BP	APOC3, APOE	2	0,002070105
**high-density lipoprotein particle remodeling**	BP	APOC3, APOE	2	0,002070105
**negative regulation of triglyceride metabolic process**	BP	APOC3, APOE	2	0,002070105
**regulation of cellular catabolic process**	BP	APOA5, APOC2, APOC3, APOE, SNCA	5	0,002070105
**regulation of hydrogen peroxide metabolic process**	BP	PRDX2, SNCA	2	0,002227947
**regulation of phospholipase activity**	BP	APOC2, SNCA	2	0,002227947
**negative regulation of platelet activation**	BP	APOE, F2	2	0,002227947
**lipoprotein catabolic process**	BP	APOC2, APOE	2	0,002227947
**negative regulation of vascular permeability**	BP	APOE, PTPRJ	2	0,002733767

**negative regulation of lipoprotein particle clearance**	BP	APOC2, APOC3	2	0,00298796
**positive regulation of triglyceride metabolic process**	BP	APOA5, APOC2	2	0,003250589
**positive regulation of fatty acid biosynthetic process**	BP	APOA5, APOC2	2	0,003521368
**negative regulation of lipid metabolic process**	BP	APOC2, APOC3, APOE	3	0,00360675
**regulation of platelet-derived growth factor receptor signaling pathway**	BP	PTPRJ, SNCA	2	0,003762021
**regulation of receptor-mediated endocytosis**	BP	APOA5, APOC2, APOC3	3	0,004487768
**biological process involved in interaction with symbiont**	BP	APOE, F2, PLG	3	0,004546162
**plasma lipoprotein particle assembly**	BP	APOC3, APOE	2	0,004635192
**positive regulation of lipid catabolic process**	BP	APOA5, APOC2	2	0,004940279
**positive regulation of hydrolase activity**	BP	APOA5, APOC2, APOH, SNCA	4	0,005324636
**protein-lipid complex assembly**	BP	APOC3, APOE	2	0,005570151
**platelet activation**	BP	APOE, F2, PTPRJ	3	0,005619671
**hydrogen peroxide catabolic process**	BP	PRDX2, SNCA	2	0,005839935
**maintenance of location**	BP	APOC4, APOE, F2, SNCA	4	0,005993878
**cholesterol metabolic process**	BP	APOA5, APOE, APOF	3	0,006279263
**lipid catabolic process**	BP	APOA5, APOC2, APOC3, APOE	4	0,006279263
**regulation of reactive oxygen species metabolic process**	BP	F2, PRDX2, SNCA	3	0,006809545
**alcohol metabolic process**	BP	APOA5, APOE, APOF, SNCA	4	0,006947232
**negative regulation of protein kinase activity**	BP	APOE, PTPRJ, SNCA	3	0,006947232
**regulation of lipid transport**	BP	APOC2, APOC3, APOE	3	0,007016593

**regulation of lipoprotein particle clearance**	BP	APOC2, APOC3	2	0,007226732
**sterol metabolic process**	BP	APOA5, APOE, APOF	3	0,007226732
**secondary alcohol metabolic process**	BP	APOA5, APOE, APOF	3	0,007226732
**negative regulation of receptor-mediated endocytosis**	BP	APOC2, APOC3	2	0,00755924
**fatty acid biosynthetic process**	BP	APOA5, APOC2, APOC3	3	0,007911344
**negative regulation of kinase activity**	BP	APOE, PTPRJ, SNCA	3	0,007984376
**positive regulation of fatty acid metabolic process**	BP	APOA5, APOC2	2	0,008617313
**negative regulation of MAP kinase activity**	BP	APOE, PTPRJ	2	0,008979275
**fatty acid metabolic process**	BP	APOA5, APOC2, APOC3, SNCA	4	0,009583614
**regulation of endothelial cell proliferation**	BP	APOE, APOH, LRG1	3	0,009609342
**positive regulation of release of sequestered calcium ion into cytosol**	BP	F2, SNCA	2	0,009639192
**amyloid fibril formation**	BP	APOE, SNCA	2	0,010469981
**negative regulation of transferase activity**	BP	APOE, PTPRJ, SNCA	3	0,011141386
**regulation of vascular permeability**	BP	APOE, PTPRJ	2	0,012709793
**endothelial cell proliferation**	BP	APOE, APOH, LRG1	3	0,01307686
**regulation of kinase activity**	BP	APOE, F2, PTPRJ, SNCA	4	0,01450296
**hydrogen peroxide metabolic process**	BP	PRDX2, SNCA	2	0,01500454
**negative regulation of transport**	BP	APOC2, APOC3, APOE, SNCA	4	0,015096626
**negative regulation of cell activation**	BP	APOE, F2, PRDX2	3	0,015980547
**zymogen activation**	BP	APOH, F9	2	0,016176745

**regulation of alcohol biosynthetic process**	BP	APOE, SNCA	2	0,016176745
**platelet-derived growth factor receptor signaling pathway**	BP	PTPRJ, SNCA	2	0,016428575
**maintenance of location in cell**	BP	APOE, F2, SNCA	3	0,016428575
**monocarboxylic acid biosynthetic process**	BP	APOA5, APOC2, APOC3	3	0,016428575
**negative regulation of catalytic activity**	BP	APOC3, APOE, PTPRJ, SNCA	4	0,016588536
**regulation of neuronal synaptic plasticity**	BP	APOE, SNCA	2	0,0166841
**regulation of endothelial cell migration**	BP	APOE, APOH, PLG	3	0,018428098
**negative regulation of protein phosphorylation**	BP	APOE, PTPRJ, SNCA	3	0,018520961
**cellular lipid catabolic process**	BP	APOA5, APOC2, APOC3	3	0,018837699
**reactive oxygen species metabolic process**	BP	F2, PRDX2, SNCA	3	0,019612239
**protein processing**	BP	APOH, F9, PLG	3	0,020402727
**negative regulation of lipid biosynthetic process**	BP	APOC3, APOE	2	0,020481009
**negative regulation of phosphorylation**	BP	APOE, PTPRJ, SNCA	3	0,022524172
**receptor metabolic process**	BP	APOE, SNCA	2	0,023565846
**positive regulation of reactive oxygen species metabolic process**	BP	F2, SNCA	2	0,023565846
**regulation of protein serine/threonine kinase activity**	BP	APOE, PTPRJ, SNCA	3	0,023565846
**negative regulation of endocytosis**	BP	APOC2, APOC3	2	0,02464243
**negative regulation of endothelial cell proliferation**	BP	APOE, APOH	2	0,026410129
**tissue regeneration**	BP	APOA5, PLG	2	0,028223218

**regulation of release of sequestered calcium ion into cytosol**	BP	F2, SNCA	2	0,028529345
**negative regulation of protein serine/threonine kinase activity**	BP	APOE, PTPRJ	2	0,028529345
**regulation of amide metabolic process**	BP	APOE, SNCA	2	0,028835058
**regulation of Rho protein signal transduction**	BP	APOC3, APOE	2	0,028835058
**endothelial cell migration**	BP	APOE, APOH, PLG	3	0,028900767
**positive regulation of calcium ion transmembrane transport**	BP	F2, SNCA	2	0,029140358
**neuroinflammatory response**	BP	BPGM, SNCA	2	0,029628132
**intracellular calcium ion homeostasis**	BP	APOE, F2, SNCA	3	0,033099507
**positive regulation of transferase activity**	BP	APOE, F2, SNCA	3	0,033099507
**negative regulation of phosphorus metabolic process**	BP	APOE, PTPRJ, SNCA	3	0,033300184
**negative regulation of phosphate metabolic process**	BP	APOE, PTPRJ, SNCA	3	0,033300184
**lipid storage**	BP	APOC4, APOE	2	0,03632503
**cellular oxidant detoxification**	BP	APOE, PRDX2	2	0,037567174
**biological process involved in symbiotic interaction**	BP	APOE, F2, PLG	3	0,037673502
**negative regulation of endothelial cell migration**	BP	APOE, APOH	2	0,037853719
**calcium ion homeostasis**	BP	APOE, F2, SNCA	3	0,039511883
**carboxylic acid biosynthetic process**	BP	APOA5, APOC2, APOC3	3	0,039943935
**steroid metabolic process**	BP	APOA5, APOE, APOF	3	0,040477789
**organic acid biosynthetic process**	BP	APOA5, APOC2, APOC3	3	0,040477789
**production of molecular mediator involved in inflammatory response**	BP	F2, PRDX2	2	0,041915558

**negative regulation of protein modification process**	BP	APOE, PTPRJ, SNCA	3	0,042712561
**long-term synaptic potentiation**	BP	APOE, SNCA	2	0,043715032
**positive regulation of lipid localization**	BP	APOC4, APOE	2	0,045786459
**plasma lipoprotein particle**	CC	APOA5, APOC2, APOC3, APOC4, APOE, APOF, APOH, PCYOX1	8	6,41E-16
**lipoprotein particle**	CC	APOA5, APOC2, APOC3, APOC4, APOE, APOF, APOH, PCYOX1	8	6,41E-16
**protein-lipid complex**	CC	APOA5, APOC2, APOC3, APOC4, APOE, APOF, APOH, PCYOX1	8	8,50E-16
**very-low-density lipoprotein particle**	CC	APOA5, APOC2, APOC3, APOC4, APOE, APOH, PCYOX1	7	1,04E-15
**triglyceride-rich plasma lipoprotein particle**	CC	APOA5, APOC2, APOC3, APOC4, APOE, APOH, PCYOX1	7	1,04E-15
**high-density lipoprotein particle**	CC	APOA5, APOC2, APOC3, APOC4, APOE, APOF, APOH	7	6,57E-15
**chylomicron**	CC	APOA5, APOC2, APOC3, APOE, APOH	5	1,20E-11
**low-density lipoprotein particle**	CC	APOC2, APOE, APOF	3	4,33E-06
**collagen-containing extracellular matrix**	CC	APOC3, APOE, APOH, F2, F9, PLG	6	3,94E-05
**blood microparticle**	CC	AFM, APOE, F2, PLG	4	0,000113378
**endoplasmic reticulum lumen**	CC	APOA5, APOE, F2, F9, PROC	5	0,000118359
**Golgi lumen**	CC	F2, F9, PROC	3	0,001145663
**early endosome**	CC	APOA5, APOC2, APOC3, APOE	4	0,006206798
**platelet alpha granule**	CC	PLG, SNCA	2	0,021097672
**secretory granule lumen**	CC	APOH, LRG1, PLG	3	0,021207717
**cytoplasmic vesicle lumen**	CC	APOH, LRG1, PLG	3	0,021207717

**vesicle lumen**	CC	APOH, LRG1, PLG	3	0,021207717
**specific granule**	CC	LRG1, PTPRJ	2	0,048687102
**serine-type endopeptidase activity**	MF	F2, F9, HABP2, PLG, PROC	5	6,94E-05
**serine-type peptidase activity**	MF	F2, F9, HABP2, PLG, PROC	5	6,94E-05
**lipase activator activity**	MF	APOA5, APOC2, APOH	3	6,94E-05
**serine hydrolase activity**	MF	F2, F9, HABP2, PLG, PROC	5	6,94E-05
**lipoprotein particle receptor binding**	MF	APOA5, APOC3, APOE	3	0,000117455
**glycosaminoglycan binding**	MF	APOA5, APOE, APOH, F2, HABP2	5	0,000130113
**heparin binding**	MF	APOA5, APOE, APOH, F2	4	0,000598357
**lipid transporter activity**	MF	APOA5, APOC4, APOE, APOF	4	0,000598357
**endopeptidase activity**	MF	F2, F9, HABP2, PLG, PROC	5	0,000933536
**lipase inhibitor activity**	MF	APOC2, APOC3	2	0,001485397
**phospholipid binding**	MF	APOA5, APOC3, APOE, APOH, SNCA	5	0,001957969
**sulfur compound binding**	MF	APOA5, APOE, APOH, F2	4	0,002194067
**cholesterol transfer activity**	MF	APOA5, APOE	2	0,002613098
**low-density lipoprotein particle receptor binding**	MF	APOA5, APOE	2	0,00285317
**sterol transfer activity**	MF	APOA5, APOE	2	0,003796558
**tau protein binding**	MF	APOE, SNCA	2	0,006853181
**cholesterol binding**	MF	APOC3, APOF	2	0,010141958
**lipid transfer activity**	MF	APOA5, APOE	2	0,013398801
**sterol binding**	MF	APOC3, APOF	2	0,014302606
**antioxidant activity**	MF	APOE, PRDX2	2	0,021524238
**alcohol binding**	MF	APOC3, APOF	2	0,02288578
**steroid binding**	MF	APOC3, APOF	2	0,030454775
**protein-folding chaperone binding**	MF	PLG, SNCA	2	0,040670992
**mannosyl-oligosaccharide mannosidase activity**	MF	MAN1A1	1	0,040670992

**intramolecular phosphotransferase activity**	MF	BPGM	1	0,040670992
**delta-catenin binding**	MF	PTPRJ	1	0,040670992
**enzyme inhibitor activity**	MF	APOC2, APOC3, SNCA	3	0,040670992
**type II transforming growth factor beta receptor binding**	MF	LRG1	1	0,040670992
**oxidoreductase activity, acting on a sulfur group of donors, oxygen as acceptor**	MF	PCYOX1	1	0,040670992
**type I transforming growth factor beta receptor binding**	MF	LRG1	1	0,040670992
**gamma-catenin binding**	MF	PTPRJ	1	0,042913092
**platelet-derived growth factor receptor binding**	MF	PTPRJ	1	0,048446482

Table S7b. Showing enriched gene ontologies from downregulated DEPs in sepsis compared to pancreatitis. BP=biological processes, CC=cellular compartments, MF=molecular functions. Ontologies classified as significantly altered if exhibiting an adjusted p-value < 0.05.

Figure S4 – KEGG pathways

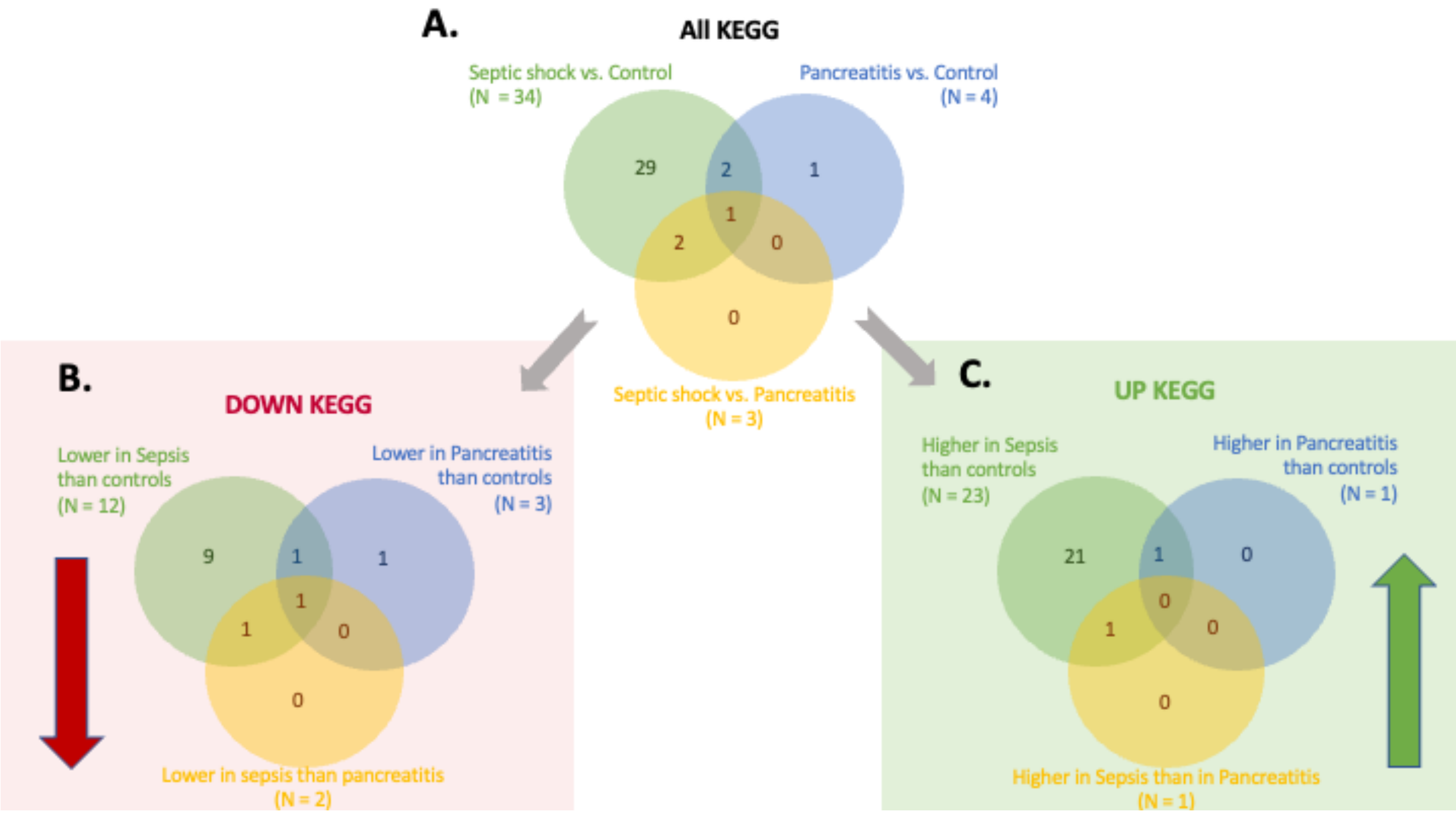

Figure S4. Venn diagrams showing the distribution of KEGG pathways in plain numbers for the comparisons Septic shock vs. Control (green circles), Pancreatitis vs. Control (blue circles) and Septic shock vs. Pancreatitis (yellow circles). Numbers in the overlapping areas representing terms significantly altered in multiple comparisons. **A.** Terms enriched from all significantly altered proteins. **B.** Terms enriched from significantly downregulated proteins. **C.** Terms enriched from significantly upregulated proteins.

Table S8a – KEGG Sepsis vs Control Up

Description	ID	geneID	Protein count	Adjusted p-value
**PI3K-Akt signaling pathway**	hsa04151	COL1A1/COL1A2/COL2A1/COL6A1/HSP90AA1/HSP90AB1/LAMB1/LAMC1/SPP1/TNC/VWF/YWHAE/YWHAG	13	0,00048542
**Prion disease**	hsa05020	HSPA8/LAMC1/PSMA1/PSMA3/PSMA4/PSMA5/PSMA6/PSMA7/PSMB2/PSMB3/PSMB4	11	0,00070699
**Parkinson disease**	hsa05012	PSMA1/PSMA3/PSMA4/PSMA5/PSMA6/PSMA7/PSMB2/PSMB3/PSMB4/TRAP1	10	0,00183748
**Huntington disease**	hsa05016	PSMA1/PSMA3/PSMA4/PSMA5/PSMA6/PSMA7/PSMB2/PSMB3/PSMB4/SOD2	10	0,00429124
**Proteasome**	hsa03050	PSMA1/PSMA3/PSMA4/PSMA5/PSMA6/PSMA7/PSMB2/PSMB3/PSMB4	9	3,667E-08
**ECM-receptor interaction**	hsa04512	COL1A1/COL1A2/COL2A1/COL6A1/LAMB1/LAMC1/SPP1/TNC/VWF	9	7,6768E-06
**Spinocerebellar ataxia**	hsa05017	PSMA1/PSMA3/PSMA4/PSMA5/PSMA6/PSMA7/PSMB2/PSMB3/PSMB4	9	0,00018219
**Focal adhesion**	hsa04510	COL1A1/COL1A2/COL2A1/COL6A1/LAMB1/LAMC1/SPP1/TNC/VWF	9	0,00126365
**Lipid and atherosclerosis**	hsa05417	HSP90AA1/HSP90AB1/HSPA4/HSPA8/ICAM1/LBP/MMP9/SOD2/VCAM1	9	0,00183748
**Cytoskeleton in muscle cells**	hsa04820	COL11A2/COL1A1/COL1A2/COL5A1/COL6A1/LMNB1/NID1/VCAN/VIM	9	0,00238044
**Human papillomavirus infection**	hsa05165	COL1A1/COL1A2/COL2A1/COL6A1/LAMB1/LAMC1/SPP1/TNC/VWF	9	0,02209993
**Amyotrophic lateral sclerosis**	hsa05014	PSMA1/PSMA3/PSMA4/PSMA5/PSMA6/PSMA7/PSMB2/PSMB3/PSMB4	9	0,04258769
**Antigen processing and presentation**	hsa04612	B2M/CALR/CTSB/CTSL/HSP90AA1/HSP90AB1/HSPA4/HSPA8	8	3,2655E-05
**Fluid shear stress and atherosclerosis**	hsa05418	CTSL/GSTO1/HSP90AA1/HSP90AB1/ICAM1/IL1R2/MMP9/VCAM1	8	0,00070699
**Protein processing in endoplasmic reticulum**	hsa04141	CALR/GANAB/HSP90AA1/HSP90AB1/HSPA8/PDIA4/PRKCSH/RRBP1	8	0,00183748

**Biosynthesis of amino acids**	hsa01230	ACY1/ALDOA/ASL/GOT1/PGK1/TALDO1/TKT	7	0,00018219
**Carbon metabolism**	hsa01200	ALDOA/GOT1/GPI/PGD/PGK1/TALDO1/TKT	7	0,00126365
**IL-17 signaling pathway**	hsa04657	HSP90AA1/HSP90AB1/LCN2/MMP9/S100A8/S100A9	6	0,00217537
**Protein digestion and absorption**	hsa04974	COL11A2/COL1A1/COL1A2/COL2A1/COL5A1/COL6A1	6	0,003303
**Lysosome**	hsa04142	CTSB/CTSD/CTSL/CTSZ/NPC2/PSAP	6	0,00974493
**Pentose phosphate pathway**	hsa00030	ALDOA/GPI/PGD/TALDO1/TKT	5	0,00019503
**Amoebiasis**	hsa05146	COL1A1/COL1A2/IL1R2/LAMB1/LAMC1	5	0,01708147
**Arginine biosynthesis**	hsa00220	ACY1/ASL/GOT1	3	0,00864517

Table S8a. Showing enriched KEGG pathways from upregulated DEPs in sepsis compared to control. Pathways classified as significantly altered if exhibiting an adjusted p-value < 0.05.

Table S8b – KEGG Sepsis vs Control Down

Description	ID	geneID	Protein count	Adjusted p-value
**Complement and coagulation cascades**	hsa04610	C1QA/C1QB/C3/C5/C6/C8A/C8B/C8G/CFH/CFI/CLU/CPB2/F10/F11/F12/F13A1/F2/KLKB1/KNG1/MASP1/PLG/PROC/PROS1/SERPINA5/SERPINC1/SERPIND1/SERPINF2	27	5,9863E-36
**Regulation of actin cytoskeleton**	hsa04810	C5/C6/C8A/C8B/C8G/F2/GSN/ITGB1/ITGB3/KNG1/MYH9/MYL12B	12	8,0335E-06
**Coronavirus disease - COVID-19**	hsa05171	ACE/C1QA/C1QB/C3/C5/C6/C8A/C8B/C8G/F13A1/F2/MASP1	12	8,0335E-06
**Prion disease**	hsa05020	C1QA/C1QB/C5/C6/C8A/C8B/C8G/NCAM1/TUBB1	9	0,00422142
**Staphylococcus aureus infection**	hsa05150	C1QA/C1QB/C3/C5/CFH/CFI/MASP1/PLG	8	2,87E-05
**Systemic lupus erythematosus**	hsa05322	C1QA/C1QB/C3/C5/C6/C8A/C8B/C8G	8	0,00025455
**Cholesterol metabolism**	hsa04979	APOA4/APOB/APOC1/APOH/CETP/LCAT	6	5,2704E-05
**Pertussis**	hsa05133	C1QA/C1QB/C3/C5/ITGB1	5	0,00461874
**Chagas disease**	hsa05142	ACE/C1QA/C1QB/C3/KNG1	5	0,0132211
**Platelet activation**	hsa04611	ADAMTS13/F2/ITGB1/ITGB3/MYL12B	5	0,02928658
**Alcoholic liver disease**	hsa04936	ADIPOQ/C1QA/C1QB/C3/C5	5	0,04757178
**Vitamin digestion and absorption**	hsa04977	APOA4/APOB/BTD	3	0,01296529

Table S8b. Showing enriched KEGG pathways from downregulated DEPs in sepsis compared to control. Pathways classified as significantly altered if exhibiting an adjusted p-value < 0.05.

Table S9a – KEGG Pancreatitis vs Control Up

Description	ID	geneID	Protein count	Adjusted p-value
**Proteasome**	hsa03050	PSMA1/PSMA6/PSMB7	3	0,03415958

Table S9a. Showing enriched KEGG pathways from upregulated DEPs in pancreatitis compared to control. Pathways classified as significantly altered if exhibiting an adjusted p-value < 0.05.

Table S9b – KEGG Pancreatitis vs Control Down

Description	ID	geneID	Protein count	Adjusted p-value
**Complement and coagulation cascades**	hsa04610	F12/F13A1/KLKB1/SERPINA5/SERPIND1	5	9,29575E-06
**Cell adhesion molecules**	hsa04514	CNTN1/NCAM1/SELL	3	0,024458582
**Vitamin digestion and absorption**	hsa04977	APOA4/BTD	2	0,013063128

Table S9b. Showing enriched KEGG pathways from downregulated DEPs in pancreatitis compared to control. Pathways classified as significantly altered if exhibiting an adjusted p-value < 0.05.

Table S10a – KEGG Sepsis vs Pancreatitis Up

Description	ID	geneID	Protein count	Adjusted p-value
**Protein processing in endoplasmic reticulum**	hsa04141	GANAB/HSP90AA1	2	0,04109991

Table S10a. Showing enriched KEGG pathways from upregulated DEPs in sepsis compared to pancreatitis. Pathways classified as significantly altered if exhibiting an adjusted p-value < 0.05.

Table S10b – KEGG Sepsis vs Pancreatitis Down

Description	ID	geneID	Protein count	Adjusted p-value
**Complement and coagulation cascades**	hsa04610	C6/F2/F9/PLG/PROC	5	8,95146E-06
**Cholesterol metabolism**	hsa04979	APOC2/APOC3/APOE/APOH	4	2,18387E-05

Table S10b. Showing enriched KEGG pathways from upregulated DEPs in sepsis compared to pancreatitis. Pathways classified as significantly altered if exhibiting an adjusted p-value < 0.05.
